# A voltage-dependent chloride channel fine-tunes photosynthesis in plants

**DOI:** 10.1038/ncomms11654

**Published:** 2016-05-24

**Authors:** Andrei Herdean, Enrico Teardo, Anders K. Nilsson, Bernard E. Pfeil, Oskar N. Johansson, Renáta Ünnep, Gergely Nagy, Ottó Zsiros, Somnath Dana, Katalin Solymosi, Győző Garab, Ildikó Szabó, Cornelia Spetea, Björn Lundin

**Affiliations:** 1 Department of Biological and Environmental Sciences, University of Gothenburg, Gothenburg 40530, Sweden; 2 Department of Biology, University of Padova, Padova 35121, Italy; 3 Laboratory for Neutron Scattering and Imaging, Paul Scherrer Institute, Villigen 5232, Switzerland; 4 Institute for Solid State Physics and Optics, Wigner Research Centre for Physics, Hungarian Academy of Sciences, Budapest 1121, Hungary; 5 Institute of Plant Biology, Biological Research Center, Hungarian Academy of Sciences, Szeged 6701, Hungary; 6 Department of Plant Anatomy, Eötvös Loránd University, Budapest 1117, Hungary; 7 CNR Neuroscience Institute, Padova 35121, Italy

## Abstract

In natural habitats, plants frequently experience rapid changes in the intensity of sunlight. To cope with these changes and maximize growth, plants adjust photosynthetic light utilization in electron transport and photoprotective mechanisms. This involves a proton motive force (PMF) across the thylakoid membrane, postulated to be affected by unknown anion (Cl^−^) channels. Here we report that a bestrophin-like protein from *Arabidopsis thaliana* functions as a voltage-dependent Cl^−^ channel in electrophysiological experiments. AtVCCN1 localizes to the thylakoid membrane, and fine-tunes PMF by anion influx into the lumen during illumination, adjusting electron transport and the photoprotective mechanisms. The activity of AtVCCN1 accelerates the activation of photoprotective mechanisms on sudden shifts to high light. Our results reveal that AtVCCN1, a member of a conserved anion channel family, acts as an early component in the rapid adjustment of photosynthesis in variable light environments.

## Abstract (web summary)

Plants have evolved to maximize energy capture while protecting their photosynthetic machinery in response to rapid variation in light conditions. Here, the authors describe a chloroplast voltage-dependent anion channel that contributes to photoprotection by fine-tuning the ion balance across the thylakoid membrane.

## Graphical abstract

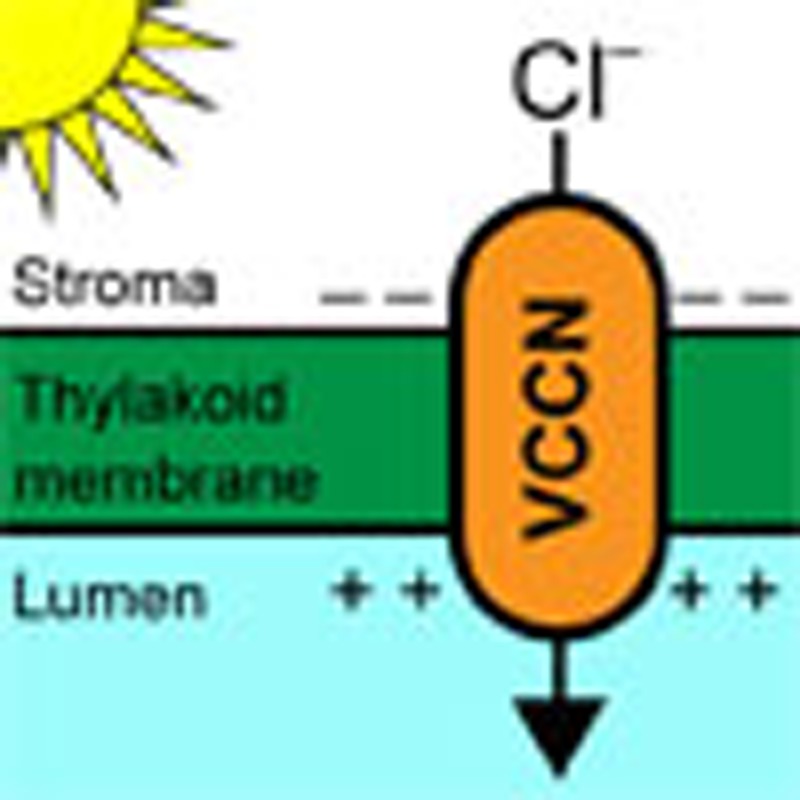

Chloroplasts have essential roles in harvesting and converting energy from the sun into carbohydrates, which are then used in cell metabolism. The protein machineries in the two aqueous compartments of this organelle (stroma and thylakoid lumen) are fine-tuned to the demands of the cell by changes in ion balance^1^. Photosynthetic electron transport in thylakoid membranes and the architecture of these membranes are highly sensitive to the concentration of ions (H^+^, K^+^, Mg^2+^ and Cl^−^) in the stroma and thylakoid lumen^1^,^2^. In natural habitats, plants experience variable light conditions, for example, shifts in light intensity and quality within seconds to minutes due to clouds, canopy architecture and leaf movement due to wind. To adjust to variable light, rapid changes in ion balance of the chloroplast occur through the regulation of ion transport^3^.

Ion channel activities across chloroplast envelopes and thylakoid membranes have been demonstrated, and are postulated to play critical roles in chloroplast physiology^4^,^5^,^6^,^7^. Light-induced charge separation and coupled H^+^ uptake into the thylakoid lumen generate a proton motive force (PMF), composed of the transmembrane electric-potential gradient (ΔΨ) and H^+^ concentration gradient (ΔpH). Both PMF components activate and drive ATP synthesis by chloroplast F_0_F_1_ ATP synthase. A high H^+^ concentration in the thylakoid lumen downregulates electron transport at the level of the cytochrome *b*_6_*f* complex and activates photoprotection through the dissipation of excess light as heat (non-photochemical quenching, NPQ)^8^. PMF partitioning into ΔpH and ΔΨ can vary with changes in the light environment, and is proposed to be a fine-tuning mechanism for photosynthesis^9^. More specifically, the plant stores PMF predominantly as ΔpH to downregulate electron transport and rapidly activate NPQ in conditions of sudden increases in light intensity, which would otherwise result in damage to the photosynthetic machinery and reduction in growth^10^. Conversely, the plant reduces the fraction of PMF stored as ΔpH after transitions to low light intensities to downregulate NPQ and maximize photosynthesis and growth. The mechanism by which PMF is partitioned and fine-tuned to achieve rapid photosynthetic acclimation is poorly understood. A critical factor is thought to be the ionic composition of the stroma and thylakoid lumen. The reasoning is that fast movements of counterions (Cl^−^ influx, Mg^2+^ and K^+^ efflux) electrically balance H^+^ uptake into the thylakoid lumen^4^. Hence, these ion fluxes would rapidly adjust PMF partitioning by efficient regulation of ΔΨ.

Our recent study in *Arabidopsis thaliana*^11^ revealed that a thylakoid K^+^ channel (AtTPK3) is involved in the modulation of PMF partitioning into ΔΨ and ΔpH through K^+^ efflux from the lumen, and in this way controls utilization of light in photosynthesis. More recently, two laboratories independently localized a K^+^/H^+^ exchanger (AtKEA3) to thylakoids and showed that it modulated PMF partitioning through H^+^ efflux from the lumen, which is critical for photosynthetic acclimation after transitions from high to low light^3^,^12^. In comparison with K^+^ transport, knowledge about the Cl^−^ fluxes that counterbalance H^+^ pumping in thylakoids is still limited, even though this is necessary to fully understand the mechanism of PMF partitioning by ions. Activities of voltage-dependent Cl^−^ channels have been reported in plant and algal thylakoid membranes^6^,^7^,^13^. However, they have not been conclusively attributed to any specific proteins. A member of the Cl^−^ channel family ClC in *Arabidopsis*, named AtClCe, was localized to the thylakoid membrane^14^,^15^,^16^. This protein was initially proposed to be involved in photosynthesis^15^, and later in NO_3_^−^ assimilation pathways^17^. Whether ClCe works as a Cl^−^ channel remains to be demonstrated. In this work, we describe a voltage-dependent Cl^−^ channel located in *Arabidopsis* thylakoids (AtVCCN1), where it functions to fine-tune PMF and allows the plant to adjust photosynthesis to variable light.

## Results

### AtVCCNs are thylakoid members of a conserved channel family

A T-DNA insertion mutant of *AtVCCN1* gene locus (*At3g61320*) was annotated to have altered NPQ by the Chloroplast Phenomics 2010 project (www.plastid.msu.edu)^18^, which identified previously uncharacterized regulatory photosynthetic proteins^19^,^20^. This gene encodes a chloroplast-predicted membrane protein, which was annotated as ‘bestrophin-like' in public databases. Bestrophins function as Ca^2+^-activated monovalent anion channels in mammalian membranes^21^.

Data extracted at ARAMEMNON database (http://aramemnon.botanik.uni-koeln.de/)^22^ indicated that the amino-acid sequence of AtVCCN1 comprises a predicted chloroplast-targeting peptide (residues 1–40), 4–5 putative transmembrane domains and termini of 60–70 residues (Supplementary Fig. 1). Using phylogenetic analyses, we found that the protein sequence was highly conserved in land plants, and that it had homologues in cyanobacteria, red and green algae, diatoms and haptophytes (Supplementary Fig. 2, Supplementary Data 1). The fact that we found homologues in non-photosynthetic bacteria and eukaryotes (fungi and arthropods) suggests a widespread importance of the biochemical function of VCCNs. No mammalian bestrophins were found in our searches, and alignment to human BEST1 revealed only 9% identity. Three residues important for anion selectivity in bestrophins^21^ were conserved, but four out of five acidic residues involved in Ca^2+^ binding were missing in AtVCCN1 (Supplementary Fig. 1). This casts doubt on the previous annotation as a ‘bestrophin-like protein', and instead suggests a family of uncharacterized anion channels. Similar to other thylakoid transport proteins such as ClCe^23^, an origin of this family from the host or symbiont in the endosymbiotic event in the evolution of chloroplasts could not be discerned in our analyses.

A close paralogue of AtVCCN1 is the protein encoded by the *At2g45870* gene, which we named AtVCCN2. Their amino-acid sequences are highly similar (76% identity; 86% similarity) and diverged during the early evolution of the Brassicaceae (c. 24–40 million years ago), but they are part of distinct clades (Supplementary Fig. 3a). AtVCCN2 also has a predicted chloroplast-targeting peptide, but we found that its transcripts were more abundant in flowers than in leaves, in contrast to *AtVCCN1* transcripts that were highly abundant both in leaves and flowers (Supplementary Fig. 3b,c), which was in agreement with Genevestigator database (https://genevestigator.com/)^24^.

**Figure 1:**
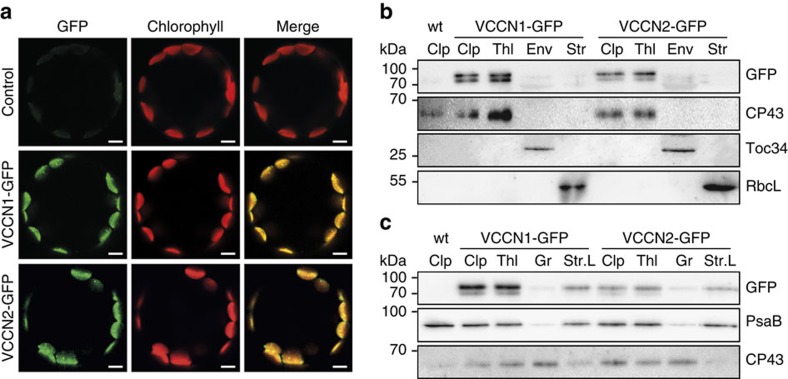
Chloroplast localization of VCCN1 and VCCN2 in *Arabidopsis*. (**a**) GFP and chlorophyll fluorescence imaged in leaf protoplasts from *Arabidopsis* wild-type plants (control) and *vccn* mutants transformed with AtVCCN-GFP fusions. Scale bars, 20 μm. (**b**,**c**) Localization of AtVCCN1 and AtVCCN2 in chloroplast and thylakoid subfractions by immunoblotting with an anti-GFP antibody. Chloroplasts (Clp), envelope (Env), stroma (Str), thylakoids (Thl), grana (Gr) and stroma lamellae (Str.L) were purified from leaves of plants transformed with AtVCCN1-GFP or AtVCCN2-GFP. Purity of the fractions was validated using antibodies against marker proteins for the respective compartments: chloroplast outer envelope membrane translocon complex Toc34 protein, ribulose bisphosphate carboxylase large subunit RbcL, photosystem I subunit PsaB and photosystem II subunit CP43.

For intracellular localization in *Arabidopsis*, we stably introduced green fluorescent protein (GFP) fusions of AtVCCN1 and AtVCCN2 into corresponding knockout mutants (*vccn1-1* and *vccn2-1*, respectively) and overexpressed them under the control of CaMV 35S constitutive promoter. In leaf protoplasts isolated from transformed plants, the GFP fluorescence signal of both fusions colocalized with chlorophyll fluorescence of chloroplasts (Fig. 1a), thereby confirming the predicted chloroplast location. Immunoblotting of chloroplast subfractions isolated from leaves of transformed plants revealed a location for both AtVCCNs in the thylakoid membrane, more precisely in the stroma-exposed regions (Fig. 1b,c, Supplementary Fig. 4a,b). Due to the lack of specific antibodies against the two proteins, the expression level of AtVCCN1 and AtVCCN2 proteins in non-transformed wild-type (wt) plants could not be assessed. However, on the basis of the tissue expression pattern, it is likely that AtVCCN1 is the predominant form in thylakoids from wt leaves.

### Electrophysiology of AtVCCN1 in planar lipid bilayers

**Figure 2:**
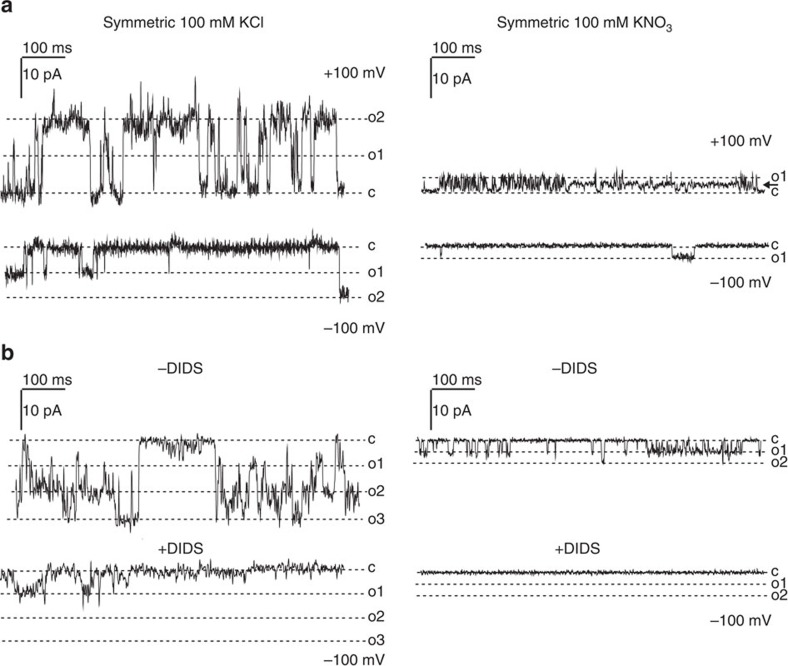
Recombinant AtVCCN1 displays a voltage-sensitive anion channel activity in planar lipid bilayers. (**a**) Current traces recorded in symmetric KCl (representative of 15 experiments) or KNO_3_ (representative of 26 experiments) solutions (*cis* side: 100 mM, *trans* side: 100 mM) at indicated potentials (*cis* side) are shown (c, closed state; o1, o2, o3, open states). The arrow in the upper right panel indicates a subconductance state. (**b**) Current traces recorded before and after addition of 20 μM DIDS (left: 100 mM KCl, *n*=4; right: 100 mM KNO_3,_ *n*=6; see also Supplementary Fig. 6c; for amplitude histograms, see Supplementary Figs 7a,8e). The general anion channel blocker DIDS caused inhibition of the activity in accordance with AtVCCN1 being an anion channel.

Previous electrophysiological studies on thylakoid membranes have revealed the activity of two distinct anion channels, one with a conductance of 110 pS in 110 mM KCl^7^,^13^ and another one with a conductance of 220 pS in 100 mM KCl^6^. We decided to study the biophysical function of AtVCCN1 in a recombinant form expressed in an *in vitro* transcription/translation system previously used for the study of various ion channels^25^,^26^,^27^ (Supplementary Fig. 5). When inserted into a planar lipid bilayer, the AtVCCN1 protein reproducibly displayed a channel activity in Cl^−^ and NO_3_^−^ media (Fig. 2a, Supplementary Figs 6,7d,8a–c,9a). The channel was selective for Cl^−^ over K^+^ displaying a permeability ratio of *P*_Cl_^−^:*P*_K_^+^ =1:0.17±0.03 (± indicates s.d., *n*=3), as determined from the reversal potential under asymmetric ionic conditions (300 mM/100 mM KCl; Supplementary Fig. 9), and calculated according to the Goldman–Hodgkin–Katz equation^28^. The single-channel conductance of AtVCCN1 in 100 mM KCl (96.1±5.0 pS and 60.0±8.2 pS at positive and negative voltages, respectively, where ± indicates s.d., *n*=5) was higher than that in 100 mM KNO_3_ (28.7±12.2 pS at both voltage ranges, where ± indicates s.d., *n*=3; Fig. 2a, Supplementary Figs 7c,8d), indicating a preference for Cl^−^ over NO_3_^−^. The channel displayed voltage dependence, being more active at positive than at negative voltages (Fig. 2a, Supplementary Figs 6,9,10). A fast-gating, flickering pattern was observed at positive voltages, whereas burst-like openings were dominant at negative voltages (Fig. 2a, Supplementary Figs 8a,10b). Furthermore, cooperative gating, that is, simultaneous opening of more than one channel and conductance sub-states (Fig. 2a, Supplementary Figs 8b,9) were observed frequently, as were changes in kinetic behaviour, that is, from slow to fast gating (Supplementary Fig. 8c). AtVCCN1 activity was blocked by general anion channel inhibitors dithiocyanatostilbene-2,2′-disulphonic acid (DIDS) and niflumic acid (Fig. 2b, Supplementary Figs 6c,7a,b,8e). Activity was affected by neither the K^+^ channel inhibitor tetraethylammonium (Supplementary Fig. 8f) nor by Ca^2+^ (Supplementary Fig. 7d), in accordance with the absence of Ca^2+^-binding motifs in the AtVCCN1 sequence (Supplementary Fig. 1). Taken together, these data demonstrate that AtVCCN1 forms a voltage-dependent, DIDS-sensitive and anion-selective channel that is permeable to Cl^−^ and NO_3_^−^. These characteristics are similar to those of the channel recorded directly in the thylakoid membrane of *Peperomia metallica* by Schönknecht *et al*.^7^ and in *Nitellopsis obtusa* by Pottosin and Schönknecht^13^. There are also differences from the previously reported activity: AtVCCN1 is less permeable to NO_3_^−^ than to Cl^−^, is also active at high voltages, and displays a different gating behaviour. The use of different species with respect to the above-mentioned works, different lipid composition and/or lack of possible regulatory subunits, post-translational modification in our system might *a priori* cause such differences. However, it cannot be excluded that AtVCCN1 and the proteins giving rise to the activities thus far characterized are distinct. The conductance and overall characteristics of AtVCCN1 were significantly different from those of another anion channel observed in thylakoids by Wagner and colleagues^6^.

### PMF and photosynthetic acclimation from dark to light

In the thylakoid membrane, the above-described activity for AtVCCN1 by electrophysiology would allow immediate Cl^−^ influx at the onset of illumination in response to an increase in thylakoid membrane voltage, that is, the ΔΨ component of PMF (positive on lumenal side) created by H^+^ uptake. Cl^−^ influx would in turn contribute to the rapid reduction in ΔΨ resulting in higher ΔpH. Conversely, defects in Cl^−^ influx would allow the total PMF to be higher and stored more as ΔΨ at the expense of ΔpH. The altered PMF size and partitioning are expected to perturb H^+^ efflux through ATP synthase, electron transport and activation of NPQ, with consequences for the acclimation of the plant after transition from dark to light and in fluctuating light conditions.

**Figure 3:**
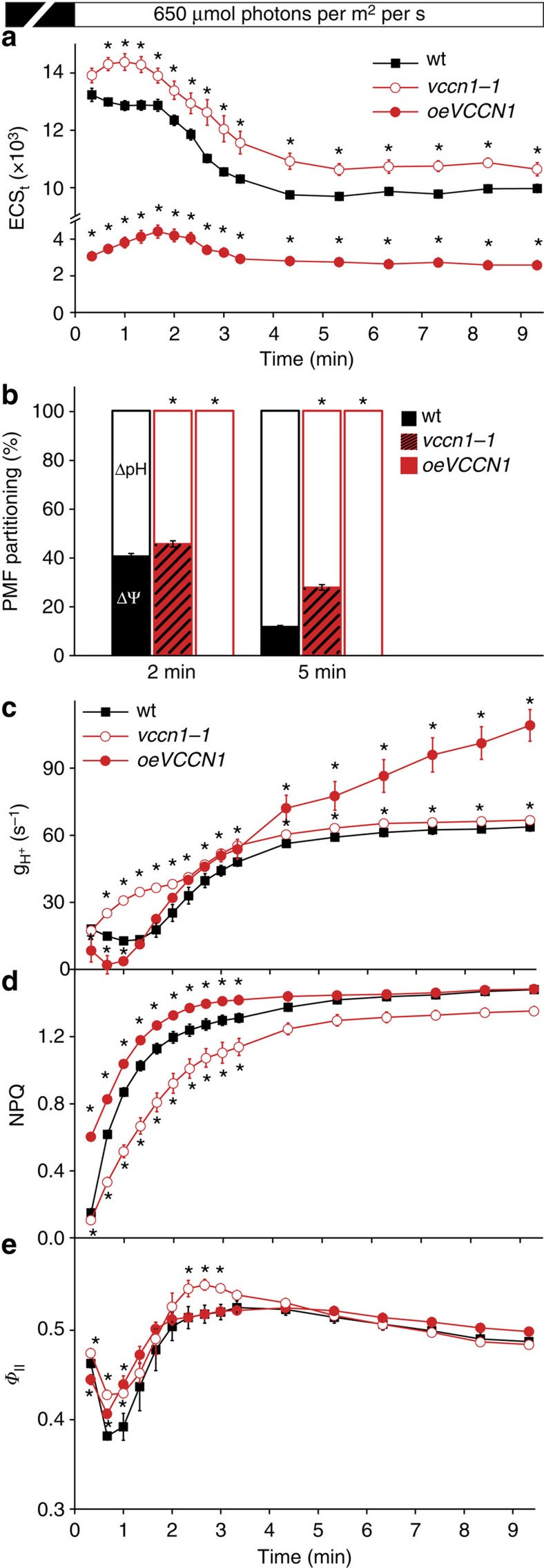
Kinetics of proton motive force and photosynthetic acclimation on transition from dark to light. (**a**) Kinetics of ECS_t_ (total electrochromic shift, a measure of total proton motive force size, PMF) in wild-type plants (wt), *vccn1-1* knockout mutant and mutant overexpressing *AtVCCN1* (*oeVCCN1*) were recorded at 650 μmol photons per m^2^ per s after 30 min dark adaptation. (**b**) Fraction of PMF contributed by transmembrane electric-potential gradient (ΔΨ) and H^+^ concentration gradient (ΔpH) at two time points in the light. (**c**) H^+^ conductivity through ATP synthase (g_H_^+^) was determined from ECS decay kinetics. (**d**) Kinetics for induction of non-photochemical quenching (NPQ) as a measure of photoprotective mechanisms were recorded during 10 min of illumination at 650 μmol photons per m^2^ per s. (**e**) Photosystem II quantum yield (*Φ*_II_) as a measure of linear electron transport was calculated from the same experiment as NPQ. Data are the means ±s.e.m. (*n*=5). Where not visible, error bars are smaller than the symbols. Asterisks denote a statistically significant difference between wt and mutants (Student's *t*-test, *P*<0.05).

To investigate the role of AtVCCN1 activity in these processes in leaves, we characterized two *Arabidopsis* T-DNA insertion knockout lines (*vccn1-1*, *vccn1-2*) and the overexpression line used in the localization of AtVCCN1-GFP (*oeVCCN1*; Supplementary Fig. 11). All genotypes grew like wt in standard conditions and displayed similar levels of photosynthetic proteins and pigments (Supplementary Fig. 12, Supplementary Table 1). To determine the total PMF size, we recorded decay of the total electrochromic shift (ECS_t_) by applying brief (600 ms) dark intervals at specific time points during illumination of intact leaves. PMF size and its partitioning into ΔΨ and ΔpH were subsequently determined from the dark interval decay kinetics of the ECS signal (Supplementary Fig. 13a,b). On transition from dark to light, PMF reached maximal values within the first 2 min, decreased by 20% until 5 min and remained stable for the remaining illumination time in wt plants (Fig. 3a). In *vccn1-1*, PMF size was moderately but significantly elevated, whereas in *oeVCCN1* it was strongly reduced as compared with wt. A significantly enhanced capacity to maintain high ΔΨ at the expense of ΔpH was observed in *vccn1-1* at both studied time points (Fig. 3b). The residual PMF observed in *oeVCCN1* consisted entirely of ΔpH, indicating the behaviour of a ‘ΔΨ uncoupler' for the overexpressed protein. These data are consistent with our hypothesis described above, that is, that AtVCCN1 is immediately activated on illumination and dissipates part of ΔΨ to maintain a significant ΔpH across the thylakoid membrane. A complementary method using pH-sensitive acridine orange (AO) fluorescence in isolated chloroplasts indicated significantly more acidic lumenal pH in the light in *oeVCCN1* than in wt (Supplementary Fig. 14), supporting the higher PMF partitioning to ΔpH in this line. In contrast to the more acidic lumenal pH, the total PMF size was lower than in wt (Fig. 3a). We do not exclude the possibility that PMF size was underestimated in o*eVCCN1*, for example, because of elevated accumulation of negative charges (Cl^−^) in the thylakoid lumen. This may have prevented the decay of the ECS signal on turning off the light during ECS measurement, which is required for estimation of PMF. This possibility does not alter the interpretation of our results for the other genotypes where decay of the ECS signal clearly takes place (Supplementary Fig. 13a,b).

Next, we determined the rate of H^+^ efflux through the thylakoid membrane (mainly via the ATP synthase) (g_H_^+^) from ECS decay during 600 ms dark intervals (Supplementary Fig. 13c,d). The observed rate was higher in *vccn1-1* than in wt throughout the illumination period (Fig. 3c), and resembled the pattern observed for PMF size rather than PMF partitioning (Fig. 3a,b). This result is in accordance with the knowledge that H^+^ efflux through the ATP synthase can be driven by both PMF components and is dependent on the total PMF size^8^. A reduced rate of H^+^ efflux was observed in *oeVCCN1* in the first minute of illumination as compared with wt (Fig. 3c). Later on during illumination, the rate increased over that of wt despite the reduced total PMF size. In control experiments, we incubated the leaves with N,N-dicyclohexylcarbodiimide (DCCD), which binds covalently to an F_0_-subunit, thus inhibiting H^+^ efflux through the ATP synthase^29^. DCCD reduced the rate of H^+^ efflux in both wt and mutants to similar levels (Supplementary Fig. 15), confirming that H^+^ efflux through the ATP synthase caused the observed differences between the three genotypes.

Chlorophyll fluorescence measurements were performed to determine the effects of AtVCCN1 on the regulation of photosynthesis. NPQ transiently decreased in *vccn1* and increased in *oeVCCN1*, resulting in an adjustment of the linear electron transport measured as PSII quantum yield (*Φ*_II_; Fig. 3d,e, Supplementary Fig. 16). On the basis of these data and the electrophysiological results described above, we suggest that AtVCCN1 is involved in light-triggered voltage-dependent Cl^−^ fluxes in thylakoids, which contribute to the counterbalancing of H^+^ pumping. In this way, AtVCCN1 fine-tunes the PMF and photosynthesis on transition from dark to light.

Although less abundant than AtVCCN1, AtVCCN2 is also expressed in leaves (Supplementary Fig. 3b,c), and may share a location in the chloroplast thylakoid membrane (Fig. 1b,c). To address possible functional redundancy between the two proteins, we characterized *vccn2*, *vccn1vccn2* and *oeVCCN2* (Supplementary Figs 11,12 and Supplementary Table 1) and compared them with wt, *vccn1* and *oeVCCN1*. We show that *vccn2* displayed a wt-like pattern for the measured parameters, whereas *vccn1vccn2* closely resembled *vccn1* (Supplementary Figs 17,18). Interestingly, the *oeVCCN2* line displayed a wt phenotype in PMF size, partitioning and rate of H^+^ efflux in the first 2–3 min of illumination, and resembled *oeVCCN1* afterwards (Supplementary Fig. 19). Pre-treatment with DCCD reduced the rate of H^+^ efflux through ATP synthase to similar levels in all six genotypes (Supplementary Fig. 15). These data suggest that AtVCCN2 is not redundant to AtVCCN1 in terms of the regulation of PMF and photosynthesis in leaf thylakoids. However, AtVCCN2 could form a channel based on the sequence similarity (Supplementary Fig. 1). Indeed, overexpressed AtVCCN2 triggered a similar response as overexpressed AtVCCN1 but with different kinetics.

### Photosynthetic acclimation from low to high light

**Figure 4:**
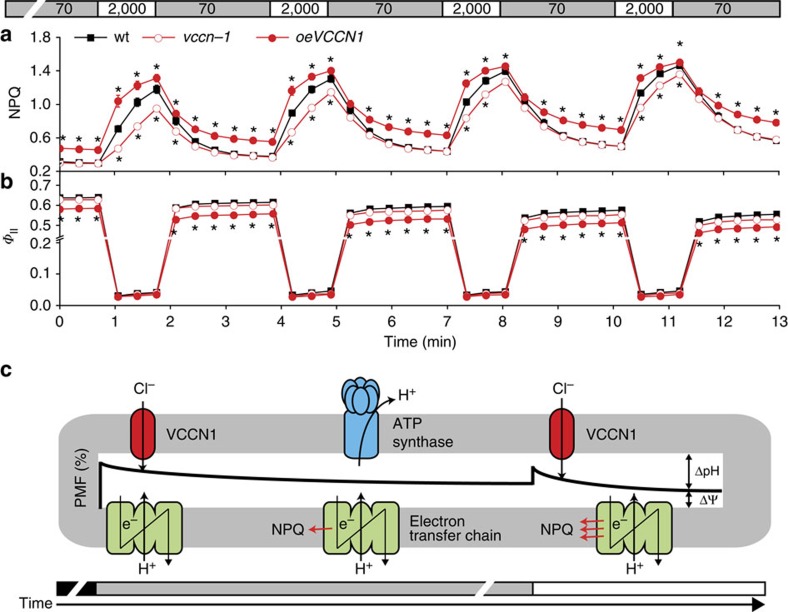
Dynamics of photosynthesis and photoprotection during transitions from low to high light. (**a**) Induction of non-photochemical quenching (NPQ) is slowed down in *vccn1-1* but approaches wild-type (wt) levels with each transition from low light (70 μmol photons per m^2^ per s) to high light (2,000 μmol photons per m^2^ per s). NPQ is induced faster in *oeVCCN1* and decays slower than in wt. (**b**) Photosystem II quantum yield (*Φ*_II_) as a measure of electron transport is not changed in *vccn1-1*, but is reduced in *oeVCCN1* when compared with the wt. Data are the means ±s.e.m. (*n*=5). Where not visible, error bars are smaller than the symbols. Asterisks denote a statistically significant difference between wt and mutants (Student's *t*-test, *P*<0.05). (**c**) Simplified model for sequence of events in the regulation of photosynthesis by AtVCCN1. At the onset of illumination (grey bar), electron transport-coupled H^+^ pumping into the thylakoid lumen results in the formation of proton motive force (PMF), consisting of a major transmembrane electric-potential gradient (ΔΨ) and a minor pH gradient (ΔpH). AtVCCN1 is activated by ΔΨ and partially dissipates it by anion influx. This phenomenon allows a rapid increase in ΔpH/ΔΨ ratio, which is maintained during the remaining illumination. AtVCCN1 also fine-tunes the size of total PMF (not illustrated in this model), which in turn regulates H^+^ efflux from the thylakoid lumen through ATP synthase. Electron transport and activation of NPQ are modulated by the ΔpH/ΔΨ ratio. After sudden shift from low light to high light (white bar), the ΔpH/ΔΨ ratio is changed by an increase in ΔΨ, but AtVCCN1 re-establishes a steady-state, resulting in rapid adjustment of photosynthesis.

To further investigate the role of AtVCCN1 in photosynthetic regulation, we undertook experiments under fluctuating light. As for the dark-to-light transition, *vccn1-1* displayed slower NPQ induction, but without significant change in linear electron transport compared with wt (Fig. 4a,b). In *oeVCCN1*, NPQ was induced faster and decayed slower, resulting in less electron transport than seen in the wt. This pattern of changes in *vccn1-1* and *oeVCCN1* can be explained by the observation that AtVCCN1 activity favours the build-up of ΔpH (Fig. 3b), and suggests its involvement in fine-tuning photosynthesis on transitions from low to high light. On the basis of the kinetic pattern of the parameters presented in Figs 3a–e and 4a,b we propose a model for the sequence of events where AtVCCN1 plays role in the regulation of photosynthesis (Fig. 4c).

### AtVCCN1 influences thylakoid ultrastructure

**Figure 5:**
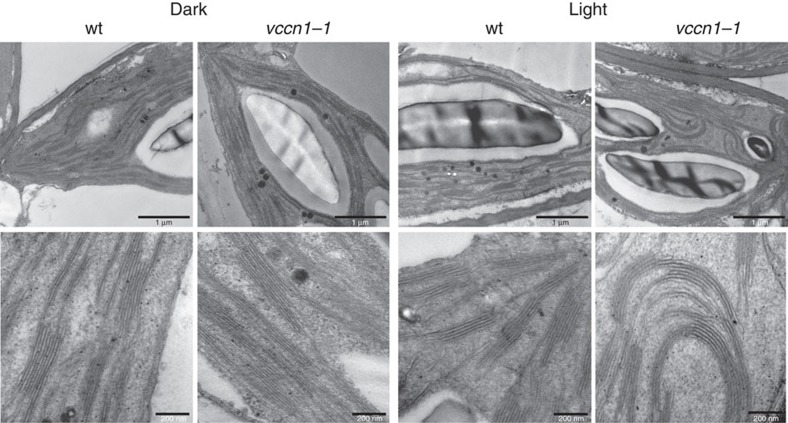
Thylakoid ultrastructure. Representative transmission electron micrographs are shown for leaf chloroplasts from 8-week-old wild-type (wt) plants and *vccn1-1* mutant fixed after 16 h of darkness or 3 h after the onset of illumination (150 μmol photons per m^2^ per s). The mutant shows consistently longer thylakoid stacks (grana) than wt in the dark state. In light conditions, the grana of *vccn1-1* are also longer, but often display a curved ‘banana-like' shape (observed in 20–50% of the studied plastid sections). Scale bars, 1 μm (upper panels) and 200 nm (lower panels).

In addition to charge compensation of light-driven H^+^ uptake in thylakoids, ion fluxes have been hypothesized to modulate the overall architecture of the thylakoid membrane because the formation of thylakoid stacks (grana) is known to depend on the ionic strength^1^,^2^. An impaired anionic permeability in *vccn1* would result in changes in intra-thylakoid ionic status and would alter thylakoid ultrastructure. Transmission electron microscopic (TEM) analyses revealed significantly longer grana in *vccn1-1* than in wt plants in the dark-adapted state (Fig. 5, Supplementary Table 2). In light-adapted plants, *vccn1-1* grana were also longer than in wt and in addition, often exhibited a curved ‘banana-like' shape rather than the horizontal shape that was commonly observed in the other samples. The overall increased granum size and modified shape in *vccn1-1* suggests significant rearrangements of the thylakoid membrane network as a result of altered ionic strength.

Studies using non-invasive small-angle neutron scattering (SANS) on detached, deuterium oxide (D_2_O)-infiltrated leaves from dark-adapted *vccn1-1* indicated a shift of the Bragg peak related to periodicity of the thylakoid stacks (grana) to higher *q* values as compared with wt (Supplementary Fig. 20a). This shift revealed a smaller lamellar repeat distance (RD) of grana in *vccn1-1* (Supplementary Table 2), which we attribute to a tighter packing than in wt. Isolated thylakoid membranes, while exhibiting larger RDs than leaves^30^, retained the small difference between the two genotypes (Supplementary Fig. 20b, Supplementary Table 2). On addition of KCl or KNO_3_, slight shrinkage of grana occurred in both samples, as indicated by a minor shift in the Bragg peak to higher *q* values, accompanied by a decrease in the Bragg peak—with this, the RD in the wt decreased to that of untreated *vccn1-1* (Supplementary Fig. 20c,d).

Circular dichroism (CD) spectra on detached leaves revealed a significantly increased (–)675-nm polymer- or salt-induced (psi)-type CD in *vccn1-1* as compared with the wt (Supplementary Fig. 21), indicating tighter stacking of grana^31^ in the mutant, in agreement with SANS data. At the same time, *vccn1-1*, similar to the wt, exhibited intense psi-type bands at around (+)505 and (+)690 nm, arising from the long-range chiral order of LHCII-PSII super-complexes^32^, suggesting that the overall macro-organization of pigment-protein complexes in grana was not perturbed by the mutation (Supplementary Fig. 21). Taken together, these data suggest the influence of AtVCCN1 on thylakoid ultrastructure in addition to its role in photosynthetic regulation.

## Discussion

Experimental evidence has recently shown that K^+^ fluxes by AtTPK3 and AtKEA3 in thylakoids regulate photosynthesis by modulation of PMF composition^3^,^11^,^12^. Activities of Cl^−^ channels have been reported previously in thylakoids and hypothesized to play role in charge counterbalance of H^+^ pumping^6^,^7^, and a member of the ClC chloride channel family, ClCe, has been located to thylakoids^14^,^15^,^16^. However, there is no experimental evidence for AtClCe being a Cl^−^ channel. Therefore, it can neither be excluded nor confirmed that ClCe contributes to the previously reported activities. In this study, we bring evidence that AtVCCN1 forms a voltage-dependent Cl^−^ channel, is located in the thylakoid membrane, and displays electrophysiological characteristics similar to those of the elusive activity previously reported. AtVCCN1 has homologues in plants, bacteria, algae, fungi and arthropods, suggesting that its function in Cl^−^ flux and membrane potential dissipation may be phylogenetically conserved, and have a widespread importance to cope with rapid changes in natural habitats. AtVCCN2 is a close paralogue, but is not functionally redundant to AtVCCN1 in leaves, where its expression is low.

Using electrophysiology in planar lipid bilayers we show that AtVCCN1 mediates flux of Cl^−^ in response to the applied voltage (Fig. 2, Supplementary Figs 6,7,9,10). In wt thylakoid membranes, the ΔΨ component of PMF is the voltage that activates and regulates AtVCCN1 to transport Cl^−^ into the lumen, which results in partial dissipation of ΔΨ (Fig. 3). A significant ΔΨ persists across wt thylakoids under steady-state conditions, in agreement with previous work by Kramer *et al*.^8^ Our observations appear to differ from those in the recent report by Johnson and Ruban^33^, who suggested that ΔΨ is completely and rapidly dissipated by ion fluxes during illumination. In our conditions, complete dissipation of ΔΨ was observed only in *oeVCCN1*, most likely due to an elevated accumulation of negative charges (Cl^−^) in the lumen of this genotype.

Through partial dissipation of ΔΨ, AtVCCN1 could also reduce the size of total PMF. Thus AtVCCN1 could be one of the factors determining the PMF size, together with the photosynthetic linear and cyclic electron transport and the H^+^ efflux through ATP synthase^8^,^34^. Overexpression of AtVCCN1 led to increased rate of H^+^ efflux through ATP synthase despite a lower PMF than in wt, without impacting plant growth under standard cultivation conditions (Fig. 3, Supplementary Fig. 11, Supplementary Table 1). Together with similar observations by Wang *et al*.^34^ for mutants affected in cyclic electron transport, our data suggest that even a lower PMF size could be sufficient for the activity of the ATP synthase during steady-state photosynthesis and plant growth. Overexpression of AtVCCN1 resulted in PMF consisting solely of ΔpH (Fig. 3), and an enhanced acidification of the thylakoid lumen (Supplementary Fig. 14). These observations are in line with the higher NPQ and lower electron transport (Fig. 3, Supplementary Fig. 19). On the basis of our results, we propose that the lower lumenal pH did not limit but rather stimulated the activity of the ATP synthase.

The wt-like phenotype of *vccn2* mutants in all studied PMF and photosynthesis parameters (Supplementary Figs 17,18) could be explained by a possible compensation by AtVCCN1 for the function of AtVCCN2. However, the double *vccn1vccn2* mutant did not display an enhanced phenotype but resembled *vccn1*. These findings together with the limited expression of AtVCCN2 in leaves make it unlikely that it is functionally redundant to AtVCCN1 in the regulation of PMF and photosynthesis in leaf thylakoids. The possibility that AtVCCN2 could form an anion channel as well, based on the close homology to AtVCCN1 and similar effects on PMF caused by its overexpression, requires further investigations.

Participation of AtVCCN1 in the regulation of PMF and photosynthesis ascribes this protein a function in the rapid acclimation of plants in variable light environments (Fig. 4). We propose that AtVCCN1 plays distinct roles as well as roles in common with AtTPK3 and AtKEA3 in photosynthetic acclimation. Like these K^+^ transport systems, AtVCCN1 modulates PMF partitioning into ΔpH and ΔΨ, affecting the balance between electron transport and photoprotective mechanisms. Distinctly, AtVCCN1 is activated immediately on the transition from dark to light by the establishment of ΔΨ, and is thus an early component modulating the PMF. Activation of AtTPK3 and AtKEA3 may require a significant ΔpH to be established by the partial dissipation of ΔΨ, for example by AtVCCN1. Furthermore, AtVCCN1 acts in photosynthetic acclimation after transitions to high light, whereas AtKEA3 activity is important for transitions to low light. Finally and distinctly from AtTPK3 and AtKEA3, AtVCCN1 alters the total PMF size and activity of ATP synthase, which may be a strategy to adjust photosynthetic carbon fixation in addition to electron transport. Our findings make AtVCCN1 an early component acting to rapidly adjust plant photosynthesis in variable light environments, and therefore it may be a target for improving photosynthetic acclimation in fields for agricultural and bioenergy production.

## Methods

### Plant material and growth conditions

*Arabidopsis thaliana* wt plants (Columbia ecotype) and mutants were grown in soil for 7–8 weeks in a growth chamber (CLF PlantMaster; Plant Climatics, Wertingen, Germany) using a daily cycle of 8 h of light (150 μmol photons per m^2^ per s) at 20 °C and 16 h of dark at 19 °C at relative humidity of 60%. The *vccn1-1* (SALK_103612) and *vccn2-1* (SALK_114715) mutants were obtained from the SALK collection^35^, *vccn1-2* (GABI_796C09) from the GABI-KAT collection^36^, and *vccn2-2* (SK_2655) from the Saskatoon collection^37^. The double *vccn1vccn2* mutant was obtained by crossing *vccn1-1* and *vccn2-1*. Reverse transcription–PCR with gene-specific primers was used to confirm the absence of transcript in the corresponding mutants (Supplementary Fig. 11b, Supplementary Table 3). *ACTIN8* (*At1g49240*) was used as the reference gene.

AtVCCN-GFP fusions were constructed as follows. The complete coding sequence of *AtVCCN1* or *AtVCCN2* (excluding native stop codons), were synthesized with flanking *att*L1 and *att*L2 sites and cloned into vector pMK-RQ (GeneArt, Life Technologies, Carlsbad, CA, USA). Gene fragments were transferred to the binary vector pB7FWG2 (ref. 38) containing the CaMV 35S promoter and eGFP at the C-termini using Gateway recombinational cloning (Invitrogen, Life Technologies). Constructs were transformed into the *Agrobacterium tumefaciens* strain GV3101 and subsequently in *Arabidopsis vccn1-1* or *vccn2-1* genetic backgrounds, respectively, using the floral dip method^39^. To select for successfully transformed plants, seeds were sown on soil and sprayed for 3 days at the fourth leaf stage with 60 mg per ml glufosinate (Basta; Hoechst Schering AgrEvo, Düsseldorf, Germany). GFP expression in transformed plants was verified using an Axio Scope A1 epifluorescence microscope (Carl Zeiss Microscopy, Göttingen, Germany). Presence of the *VCCN* transgene in transformants was confirmed by PCR and its expression verified by quantitative PCR.

### Quantitative PCR

Total RNA was isolated from plant tissues of 7-week-old plants with an E.Z.N.A. R6827-01 Plant RNA kit (Omega Bio-Tek, Norcross, GA, USA) and residual DNA was removed with E1091 DNAse (Omega Bio-Tek). Tissues pooled from 30 plants were used for each analysis. Complementary DNA (cDNA) was prepared from 400 ng RNA with an iScript cDNA Synthesis kit (Bio-Rad, Hercules, CA, USA). Quantitative real-time PCR analyses were conducted with a SsoAdvanced Universal SYBR Green Supermix on a C1000 Touch Thermal Cycler (Bio-Rad). PCR template cDNA corresponding to 40 ng total RNA was used in 15-μl reactions. Amplifications were undertaken in two-step PCR with the following cycling conditions: initial denaturation for 3 min at 95 °C, followed by 40 cycles of denaturation for 5 s at 95 °C and annealing/extension for 30 s at 60 °C. Melt-curve analyses were conducted for all primers after amplification. Expected product sizes were confirmed by agarose gel electrophoresis. Efficiencies of PCR amplification of all target genes were established from serially diluted calibration curves of wt cDNA prepared from leaf tissue. Gene-specific primers used are listed in Supplementary Table 3. Relative expression was calculated with the ΔCq method (2^-ΔCq^) using *PEX4* (*At5g25760*) as the reference gene.

### Subcellular localization of AtVCCN-GFP fusion proteins

Protoplasts were prepared using the Tape-Arabidopsis Sandwich procedure from leaves of transgenic plants (T2 generation) expressing *AtVCCN-GFP*^40^. Protoplasts were mounted in W1 solution (0.5 M mannitol, 20 mM KCl, 4 mM MES-KOH, pH 5.7) and observed using a LSM 700 inverted Axio Observer Z1 confocal laser scanning microscope (Carl Zeiss Microscopy, Jena, Germany) equipped with a LD C-Apochromat water immersion objective lens (× 40/1.1 NA). GFP and chlorophyll fluorescence were both excited at 488 nm and emitted signals were collected above 640 nm for chlorophyll fluorescence (long-pass filter) and below 548 nm for GFP fluorescence (variable secondary dichroic beamsplitter). Images were analysed with Zen 2012 blue edition software (Carl Zeiss Microscopy).

### Electrophysiology measurements

The *AtVCCN1* gene was PCR-amplified without the targeting peptide (corresponding to the first 40 amino acids) from *Arabidopsis* leaf cDNA introducing the restriction sites *NdeI* (5′) and *SacI* (3′) and cloned into the pIVEX1.4 WG vector (Roche, Basel, Switzerland) in fusion with a 6 × His-tag. Gene-specific primers used are listed in Supplementary Table 3. *In vitro* expression was conducted using a RTS 100 Wheat Germ CECF kit (Roche Diagnostics, Basel, Switzerland) according to manufacturer's instructions. The expressed protein present in the lysate, which does not contain other membrane proteins, was solubilized with 2% (w/v) Triton X-100.

Experiments involving planar lipid bilayers were carried out as described previously^11^. Briefly, a BC-525C electrophysiological planar bilayer apparatus (Warner Instruments, Hamden, CT, USA) was used. Bilayers with a capacity of ∼150–200 pF were prepared by painting a decane/chloroform solution of soybean asolectin (Sigma-Aldrich, St Louis, MO, USA), purified by precipitation with cold acetone from a chloroform solution, across a 250-μm hole in a polystyrene cuvette. Solutions consisting of 100 mM KCl or 100 mM KNO_3_ in 10 mM Hepes-KOH pH 7.0 were used. For ionic gradient experiments, 300 mM and 100 mM KCl in the same buffer were used on the *cis* and *trans* side, respectively. Contents of both chambers were stirred by magnetic bars when necessary. Connections to electrodes were provided by agar bridges. The protein expressed in wheat germ lysate was added to the *cis* side. Addition of empty wheat germ lysate never resulted in channel activity (Supplementary Fig. 5b). Voltages reported are those of the *cis* chamber, and current is considered to be positive when carried by cations flowing from the *cis* compartment to the *trans* compartment. The recorded data were analysed using the pCLAMP8.0 program set (Molecular Devices, Sunnyvale, CA, USA).

### Protein and pigment analyses

For localization studies, chloroplasts were isolated from leaves of dark-adapted plants, which were grinded in preparation media (50 mM Hepes-KOH pH 7.8, 330 mM Sorbitol, 10 mM KCl and 1 mM EDTA), filtrated through two layers of Miracloth and subsequently centrifuged at 3,000*g* for 3 min at 4 °C. The pellet representing the crude chloroplast fraction was resuspended in preparation buffer, loaded and centrifuged in a 10% (v/v) Percoll solution at 3,000*g* for 10 min (ref. 41). After lysis of purified chloroplasts, thylakoids and envelope were purified on a four-step sucrose gradient by centrifugation at 70,000*g* for 1 h at 4 °C (ref. 42). Thylakoid subfractions, grana and stroma lamellae, were isolated by solubilization of thylakoids with digitonin^43^. Thylakoids at 0.5 mg chlorophyll per ml in 25 mM Tricine-KOH pH 7.8, 100 mM Sorbitol, 5 mM MgCl_2_, 10 mM KCl and 10 mM NaF were solubilized with 1% (w/v) digitonin by gentle stirring for 7 min at room temperature. The suspension was centrifuged at 1,000*g* for 5 min to remove insolubilized material. The supernatant was further centrifuged at 40,000*g* for 30 min and at 140,000*g* for 90 min, resulting in the grana and the stroma lamellae fractions. Chlorophyll concentration of various fractions was determined following extraction in 80% (v/v) acetone for 5 min at 4 °C and spectrophotometry^44^.

SDS/urea/PAGE and immunoblotting using antibodies against various proteins were conducted as described by Yin *et al*.^45^ Antibodies against PsbS (AS09533, 1/2,000), Lhcb2 (AS01003, 1/20,000), Cytf (AS08306, 1/2,000), PsaA (AS06172, 1/1,000), PsaB (AS10695, 1/1,000), AtpB (AS05085, 1/1,000), Toc34 (AS07238, 1/1,000), and RbcL (AS03037, 1/10,000) were purchased from Agrisera (Vännäs, Sweden). The antibodies against D2 and CP43 proteins from spinach (1/5,000) were kindly provided by Eva-Mari Aro (Turku University) and Torill Hundal (Stockholm University). Anti-GFP antibody (Cat#11814460001, 1/5,000) was obtained from Roche Diagnostics (Indianapolis, IN, USA). Immunodetection was done using an ECL system WesternBright Quantum (Science Imaging Scandinavia, Stockholm, Sweden) and visualized using Fusion SL7 (Vilbert Lourmat, Eberhardzell, Germany). Uncropped versions of the immunoblots from Fig. 1 and Supplementary Fig. 12 are shown in Supplementary Figs 4 and 22, respectively.

Chlorophyll and carotenoid contents of leaf discs were determined following extraction in 96% (v/v) ethanol for 10 min at 60 °C and spectrophotometry^46^.

### Measurements of chlorophyll *a* fluorescence

Slow kinetics of chlorophyll fluorescence induction were recorded with a pulse-amplitude modulated fluorometer DUAL-PAM 100 (Walz, Effeltrich, Germany) on attached leaves of 30-min dark-adapted plants at room temperature at actinic red light of given intensity. NPQ and *Φ*_II_ were calculated as (*F*_m_*–F*_m_′*)/F*_m_′ and (*F*_m_′*–F)/F*_m_′, respectively^47^, where *F*_m_ is maximum fluorescence yield, *F*_m_′ is maximum fluorescence yield in the light, and *F* is variable fluorescence yield in the light.

### Electrochromic band shift measurements

ECS was recorded with a DUAL-PAM 100 system (Walz) equipped with a P515/535 emitter/detector module as absorbance difference signal 550–515 nm according to Walz protocols^48^. First, plants were dark adapted for 30 min, and then exposed to actinic red light of given intensity for ≤10 min. The light was turned off and the ECS decay kinetics were recorded to determine total ECS (ECS_t_) representing total PMF size and its components (ΔpH and ΔΨ) according to the method of Cruz and colleagues^49^. Before each measurement, three pulses of 5-μs and 200,000 μmol photons per m^2^ per s were applied and the signals were averaged to determine ECS_ST_, which was used to normalize the ECS_t_ values of each measurement. Normalization was done by multiplying ECS_t_ with a correction factor calculated as (maxECS_ST_/*ECS*_*ST*_), where maxECS_ST_ is the highest ECS_ST_ from all the measurements of a dataset, and *ECS*_*ST*_ is the ECS_ST_ corresponding to the measurement of the ECS_t_ that is being normalized. To determine H^+^ conductivity of the thylakoid membrane through ATP synthase (g_H_^+^), the light was turned off at specific time periods and the decay of the ECS signal was recorded during 600 ms dark intervals. The ECS decay was fitted with a single exponential function for the first 100 ms, and g_H_^+^ was calculated as 1/time constant for decay^50^. Where indicated, leaf discs were incubated for 10 min in a solution containing 1% (v/v) Tween 20, 2% (v/v) DMSO and 5 mM DCCD before illumination and ECS recording.

### Measurement of pH changes by AO fluorescence

The fluorescent probe AO was used to measure thylakoid membrane energization^51^,^52^. Although a simple relation between fluorescence quenching attributable to accumulation of protonated amines in the lumen and pH has been proposed, this method only allows a semiquantitative indication of pH changes^51^,^52^. Intact chloroplasts were isolated from *Arabidopsis* leaves and purified by a two-step Percoll gradient centrifugation^53^. Time-resolved measurements of AO using *Arabidopsis* chloroplasts were performed with a PAM101/102/103 fluorometer (Walz, Effeltrich, Germany) endowed with a GFP filter set (excitation at 480 nm; cutoff, <505 nm; emission with long-pass filter, 515 nm; cutoff, 575 nm). Actinic light was applied by fibre optic and was low enough to avoid actinic effects. AO fluorescence was measured in darkness and during illumination for 100 s at 700 μmol photons per m^2^ per s. Chlorophyll concentration was adjusted to 10 μg per ml in the purification resuspension buffer (20 mM Hepes-KOH pH 7.6, 0.4 M Sorbitol, 2.5 mM EDTA, 5 mM MgCl_2_, 10 mM NaHCO_3_, 0.15% (w/v) BSA) and the chloroplast suspension was preincubated with 1 μM AO for 20 min in darkness before measurement.

### Transmission electron microscopy

Sections (∼1 mm × 1 mm) cut from the central parts of leaf blades of 8-week-old plants were fixed in 2.5% (v/v) glutaraldehyde for 4 days, then post-fixed in 1% OsO_4_ (w/v) for 2 h. Fixatives were buffered with 70 mM Na_2_HPO_4_–KH_2_PO_4_ pH 7.2. After fixation steps, samples were rinsed in the same buffer. After dehydration in an alcohol series, samples were embedded in Durcupan ACM resin (Fluka, Buchs, Switzerland). Ultrathin sections (thickness 50–60 nm) were cut with an UC6 ultramicrotome (Leica Microsystems, Vienna, Austria) fitted with a diamond knife (Diatome, Hatfield PA, USA). Sections were mounted on copper grids and contrasted with 5% uranyl acetate and Reynolds' lead citrate solution. TEM was conducted with a LEO 912AB Gemeni system (Carl Zeiss, Oberkochen, Germany) equipped with a LaB6 emitter at an accelerating voltage of 120 kV using a Veleta CCD camera and iTEM software (Olympus Soft Imaging Systems, Münster, Germany).

ImageJ software was used to measure granum diameter (measured at the middle of perpendicular granum sections) on transmission electron micrographs. Calculations were done on randomly chosen grana (350–450) originating from 60–90 different plastids taken randomly from 60–90 different mesophyll cells from leaves of two different plants per treatment. Statistical analyses were undertaken using InStat v.6.0 (GraphPad, La Jolla, CA, USA). Ultrastructural data did not follow a normal distribution, so Kruskal–Wallis non-parametric ANOVA was used followed by the Mann–Whitney rank-sum test. For all data, *P*<0.05 was considered significant.

### SANS measurements

SANS measurements were carried out on the SANS-I (leaves) and SANS-II instruments (isolated thylakoid membranes) at the Paul Scherrer Institute (Villigen, Switzerland). On SANS-I and SANS-II, we used a wavelength of 6 Å, collimation of 11 and 6 m, and sample-detector distance of 11 and 6 m, respectively. Detached leaves were infiltrated in D_2_O to enhance contrast. Owing to the relatively weak Bragg peak in *Arabidopsis* leaves compared with several other species^30^, for each measurement 8–10 leaves were placed in a quartz cuvette (path length, 2 mm) filled with D_2_O. To improve the signal-to-noise ratio, experiments were repeated three times for *vccn1-1* and four times for wt, and the SANS curves were averaged. Hence, these SANS profiles represent the statistical average of ∼25–30 leaves. Thylakoid membranes, isolated as described by Posselt and colleagues^54^, were suspended in a D_2_O-containing reaction medium (20 mM Tricine, pD 7.6, 0.4 M sorbitol, 5 mM MgCl_2_, 5 mM KCl) and measured at a chlorophyll concentration of ∼500 μg per ml in a quartz cuvette (optical path length 2 mm) placed in a magnetic field of 0.4 T.

Primary steps of data analyses and radial averaging in a 360° sector (leaves) and in two 75° sectors (isolated thylakoid membrane) of the two-dimensional scattering profiles were performed with the Graphical Reduction and Analysis SANS Program package (GRASP) developed by C. Dewhurst (Institut Laue-Langevin, Grenoble, France). RD of the thylakoid membrane was calculated from the position of the characteristic Bragg peak, which corresponded to the periodicity of grana thylakoid membranes. For this, SANS curves in 0.0151–0.0380 Å^−1^ (leaves) and 0.0194–0.0310 Å^−1^ (isolated thylakoids) regions were fitted with equation (1):


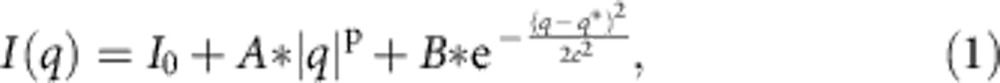


where *I* is the scattered intensity, *I*_0_, *A* and *B* are constants, *q* is scattering vector, q* is position of the Bragg peak, *p* and *c* are parameters of the power function and Gaussian distribution, respectively^55^. The q* parameter was used to calculate RD with equation (2):


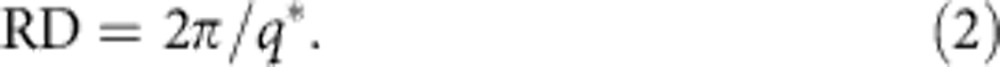


For detached leaves, statistically averaged RD values for 25–30 leaves per treatment are given; the corresponding error values arise from uncertainty of the fitting, containing the statistical error of the scattering intensity data points. SANS profiles of untreated and salt-treated thylakoids were measured on freshly isolated membranes; the variation of RD values among repeated measurements within 3 h was <1 Å. Representative one-dimensional plots and RD values with the error of fitting are presented.

### CD spectroscopy

CD measurements were made on a J-815 spectropolarimeter (JASCO, Tokyo, Japan). Detached, water-infiltrated leaves were placed between two glass slides in an optical cell. Spectra were recorded at room temperature between 400 and 800 nm at a scan speed of 100 nm per min, band-pass of 3 nm and step size of 1 nm. For each sample, 3–4 scans were averaged. Spectra were normalized to the absorption of the red-most peak of the spectra recorded at the same time as the CD spectra and were corrected for baseline distortions. Measurements were repeated on three different leaves for each genotype. Amplitudes of psi-type CD bands, at around (+)505, (–)675 and (+)690 nm, were determined using the reference wavelengths of 550, 600 and 750 nm, respectively.

### Phylogenetic analyses

To identify candidates for the most closely related eukaryotic protein sequences to AtVCCN1, we used BlastP searches with ‘AtVCCN1' as the query against the GenBank database^56^. Initially, we selected best matches from representative green plant lineages to build up the probable orthologous group of sequences from these lineages. Sampled lineages included the land-plant families Brassicaceae, Fabaceae, Solanaceae, Vitaceae, Poaceae, Pinaceae, Selaginellaceae, Funariaceae, and the green alga families Chlorellaceae and Chlamydomonadaceae.

Non-green-plant best matches were very few and had low *E* values. Among the best matches were representatives of haptophytes (*Emeliana E*=7 × 10^−40^), heterokontophytes (*Phaeodactylum E*=5 × 10^−37^) and cyanobacteria (*Synechococcus E*=9 × 10^−28^), with 16–25% maximum identity to AtVCCN1 (based on MUSCLE alignment). Specific searches to bacteria also retrieved the same cyanobacterium best match as observed previously. We included representative matches of each of these lineages in our alignments (Supplementary Data 1).

Specific searches for opisthokonta targets (the clade including animals and fungi) yielded matches with *E* values ≤10^−15^, with matches between 10^−15^ and 10^−11^ (the top 10 matches) all from fungi, with 12–14% maximum identity to AtVCCN1 (Supplementary Data 1). Representative fungal sequences as well as BEST1 from human were included to evaluate the type of homology present between AtVCCN1 and BEST1 (that is, if they are orthologous, paralogous, or not even homologous).

Sequences were aligned using automated methods: MAFFT, MUSCLE, T-COFFEE and CLUSTAL-O (default values in each case, using the EMBL server at www.ebi.ac.uk/Tools/msa/). BEST1 and AtVCCN1 sequences were compared using a BLOSUM62 matrix. Then, preliminary neighbour-joining (NJ) analyses were conducted on alignments. When including BEST1 from humans, only 9% identity was found with AtVCCN1 (MUSCLE alignment in Supplementary Fig. 1), and so this sequence was excluded from the main phylogenetic analyses. Neighbour-joining trees were used to assess which branches in the phylogeny were particularly long in comparison with others in the tree. Further searches in BlastP were conducted using sequences on long branches to sample additional closely related sequences. These were added to the tree so that these long branches would be subdivided, thereby making later phylogenetic estimates likely to be more robust^57^. The best BlastP match to red algae (*Galdieria*, *E*=5 × 10^−22^, 32% maximum identity) was also included because this group can help to understand the host versus the symbiont origin of genes^23^.

The sequence from all taxa corresponding to the chloroplast-targeting region in AtVCCN1 (residues 1–40) and of the LALD amino-acid motif common to most land plants, were trimmed before running the main analyses. These analyses were conducted using the Bayesian inference in MrBayes 3.2.2 (ref. 58): (a) using the same trimmed MAFFT alignment as described previously for all taxa except human BEST1 (confirmed using PhyML^59^), and (b) using the corresponding DNA sequences from the genes found only in Brassicaceae and Vitaceae, with the addition of a Cleomaceae sequence (sister family to Brassicaceae^60^), to clarify when the gene duplication that produced *VCCN1* and *VCCN2* occurred. MrBayes settings included reversible model jump MCMC over the substitution models, four chains, and paired runs for 5 million generations.

### Data availability

The data that support the findings of this study are available from the corresponding authors on request.

## Additional information

**How to cite this article:** Herdean, A. *et al*. A voltage-dependent chloride channel fine-tunes photosynthesis in plants. *Nat. Commun.* 7:11654 doi: 10.1038/ncomms11654 (2016).

## Supplementary Material

Supplementary Information Supplementary Figures 1 - 22, Supplementary Tables 1 - 3 and Supplementary References

Supplementary Data 1 Expanded version of Supplementary Figure 2 and MAFTT alignment.
