# Supplementary material for: A voltage-dependent chloride channel fine-tunes photosynthesis in plants
Source: Nat Commun. 2016 May 24;7:11654. doi: 10.1038/ncomms11654 (PMC4890181; doi:10.1038/ncomms11654)
Supplement: Supplementary Information — Supplementary Figures 1 - 22, Supplementary Tables 1 - 3 and Supplementary References [file ncomms11654-s1.pdf]

```
BEST1_HUMAN      -------------MTITYTSQVANA-------RLGSFSRLLLCWRGSIYKLLYGEFLIFLL
VCCN1_arath      ---------------VSSGPESNDSGHETLTDKLIHLLRAVPDWADEIKERGMQQKRSLYT
VCCN2_arath      RAPKSLHFKFNPSCVSSGPKSDDS---PLSEKLISLLKAVPNWSDGIKERRMQQKRSLYT
                               ::  .:  ::       .*  : . :  * . * :    :   :

                                                   Anion selectivity
BEST1_HUMAN      CYYIIRFIYRLALTEEQQLMFEKLTLYCDSYIQLIPISFVLGFYVTL-VVTRWWNQYENL
VCCN1_arath      HEKWVE--HRSSLRHVRHLLS---SFSSRVILSLIPPVF---FFTSVAVVIASYNSAVAL
VCCN2_arath      HENWVR--HRSSLRHLRHVSS---SPSSRVILSLIPPVF---FFTTVAILIAGYNSAVDL
                     :   :* :*   .::     :  .   :.***  *   *:.:: ::   :*.   *

BEST1_HUMAN      PWPDRLMSLVSGFVEGKDEQGRLLRRTLIRY----ANLGNVLILRSVSTAVYKRFPSAQH
VCCN1_arath      DW-------LPGIFP-------ILRSSSLPYQLTAPALALLLVFRTEAS--YSRYEEGR-
VCCN2_arath      DW-------LPDFFP-------VLRASPLPYQLTAPALALLLVFRTEAS--YSRFEQGR-
                  *       :..:.        :** : : *    . *. :*::*: ::  *.*: ...

BEST1_HUMAN      LVQAGFMTPAEHKQLEKLSLPHNMFWVPWVWFANLSMKAWLGGRIRDPILLQSLLNEMNT
VCCN1_arath      -------------------------------------KAWVG-----------IIAGTND
VCCN2_arath      -------------------------------------KAWVK-----------IISGTND
                                                      ***:            ::   *

BEST1_HUMAN      LRTQCGHLYAYDWISIPLVYTQVVTVAVYSFFLTCLVGRQFLNPAKAYPGHELDLVVPVF
VCCN1_arath      L-------------------ARQVICSVDS------------------SGDELIIKDLLL
VCCN2_arath      L-------------------ARLVISSVHG------------------SGDELIIRDALL
                 *                   :. *  :* .                  .* ** :   ::

                                    Ca2+clasp
BEST1_HUMAN      TFLQFFFYVGWLKVAEQLINPFGEDDDDFETNWIVDRNLQVSLLAVDEMHQDLPRMEPDM
VCCN1_arath      RYIAAF------PVALKCHVIYGSD---------IARDLR-NLIEADDLSLILQAK----
VCCN2_arath      RYIVAF------PVALKCHVIYGSD---------IASDLK-NVIEVDDLSLILQSK----
                  ::  *       ** :    :*.*         :  :*. .:: .*::   *

BEST1_HUMAN      YWNKPEPQPPYTAASAQFRRASFMGSTFNISLNKEEMEFQPNQEDEEDAHAGIIGRFLGL
VCCN1_arath      --HRPRCVIEFISQSIQL-----------LKLDDAKRDLLESKMLHLHEGIGVCEQLMGI
VCCN2_arath      --HRPRCVIQFISQSLQL-----------LNLDSTKIDMLETKMMQLQEGIGVCEQLMGI
                   :.*     : : * *:           :.*:. : ::  .:        *:  .::*:

BEST1_HUMAN      QSHDHHPPRANSRTKLLWPKRESLLHEGLPKNHKAAKQNVRGQEDNKAWKLKAVDAFKSA
VCCN1_arath      PIPLSY-TRLTSRFLVFW-------HLTLP-----------------------------I
VCCN2_arath      PIPLSY-TRLTSRFLVLW-------HLTLP-----------------------------V
                      : .* .**  ::*       *  **

BEST1_HUMAN      PLYQRPGYYSAPQTPLSPTPMFFPLEPSAPSKLHSVTGIDTKDKSLKTVSSGAKKSFELL
VCCN1_arath      ILWDECHWIVVPATFISAASLF----------------------CIEEVGVLIEEPFPML
VCCN2_arath      ILWDDCHWNVVPATFISAASLF----------------------CIEEVGVLIEEPFSML
                  *::   :  .* * :*.:.:*                      .:: *.   ::.* :*

BEST1_HUMAN      SESDGALMEHPEVSQ-VRRKTVEFNLTDMPEIPENHLKEPLEQSPTNIHTTLKDHMDPYW
VCCN1_arath      ALDELCDLVHSNIQEAVKSEKVIRNRI----IAKIKLHE-FKHSSNGRHRS---------
VCCN2_arath      ALDELCAMVLSNSDEAVESKEVIRNRI----IAKKRILE-IKHSSNGWHKS---------
                 : .: . :  .: .: *  : *  *      *.: .: * :::*... * :

BEST1_HUMAN      ALENRDEAHS
VCCN1_arath      ----------
VCCN2_arath      ----------
```

Supplementary Figure 1. Sequence alignment of *Arabidopsis* VCCNs and human bestrophin BEST1 (UniProt O76090) using MUSCLE. Despite the removal of the putative chloroplast-targeting sequence from VCCNs, the alignment showed only 9% identity and several gaps. The putative transmembrane domains in VCCNs according to a consensus at ARAMEMNON database are marked with borderline. A continuous borderline denotes high score for hydrophobicity (>0.5), broken borderline denotes a low score (0.2 < score < 0.5), whereas dotted borderline represents a score of 0.2. A few transmembrane domains in BEST1, marked according to the crystal structure by Kane Dickson and colleagues[1] are present in VCCNs, suggesting a different protein architecture. Three residues important for anion selectivity in BEST1 are conserved in VCCNs (shown in red). From the 'Ca^{2+} clasp' in BEST1 comprising five acidic residues only one is conserved in VCCNs (shown in blue).

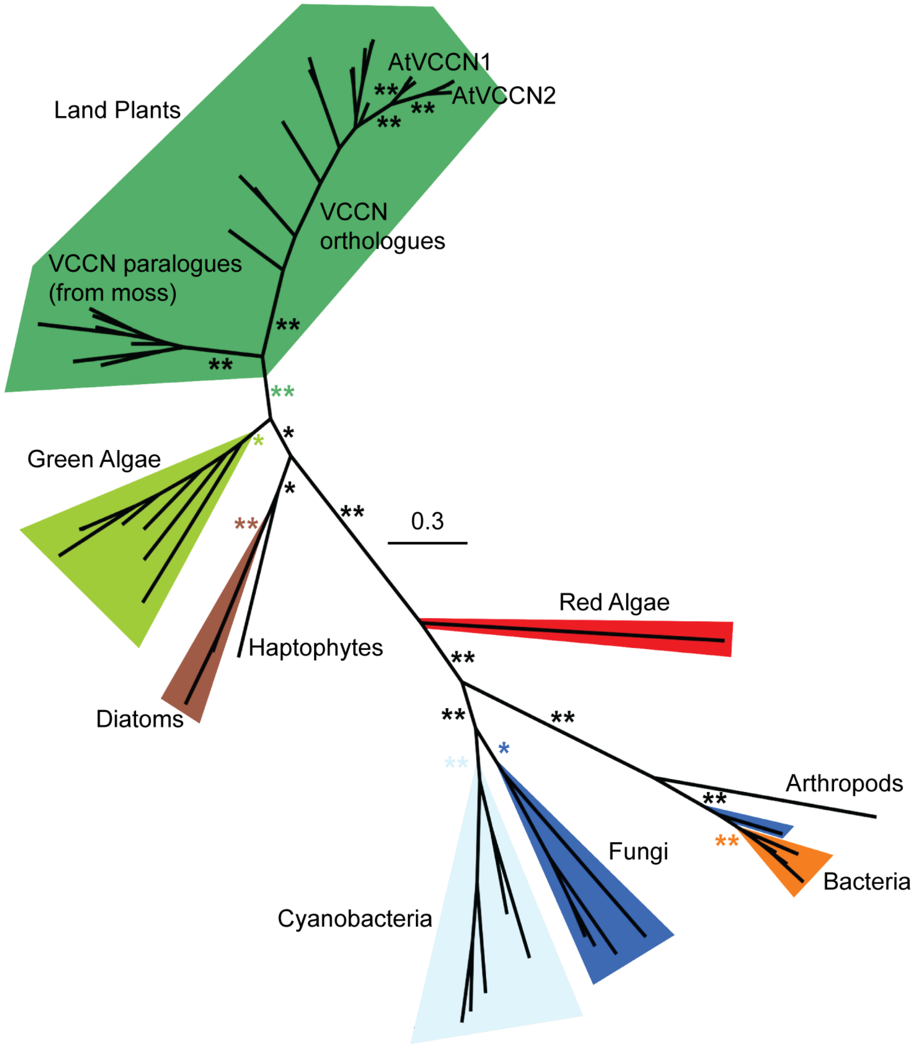

Supplementary Figure 2. VCCN phylogeny. Maximum clade credibility tree of AtVCCN1-related proteins is shown. Analyses are based on the MAFFT alignment presented in Supplementary Data 1 and trimmed as described in the Methods. Named clades and major internal branches marked with a single asterisk have ≥0.95 posterior probability; clades marked with two asterisks also have maximum likelihood bootstraps ≥80% (minor clades are not marked). The scale bar indicates substitutions per site. Within land plants, two clades of proteins were found; the moss clade probably arose from gene duplication early in the land plant lineage, and is thus paralogous to the VCCNs.

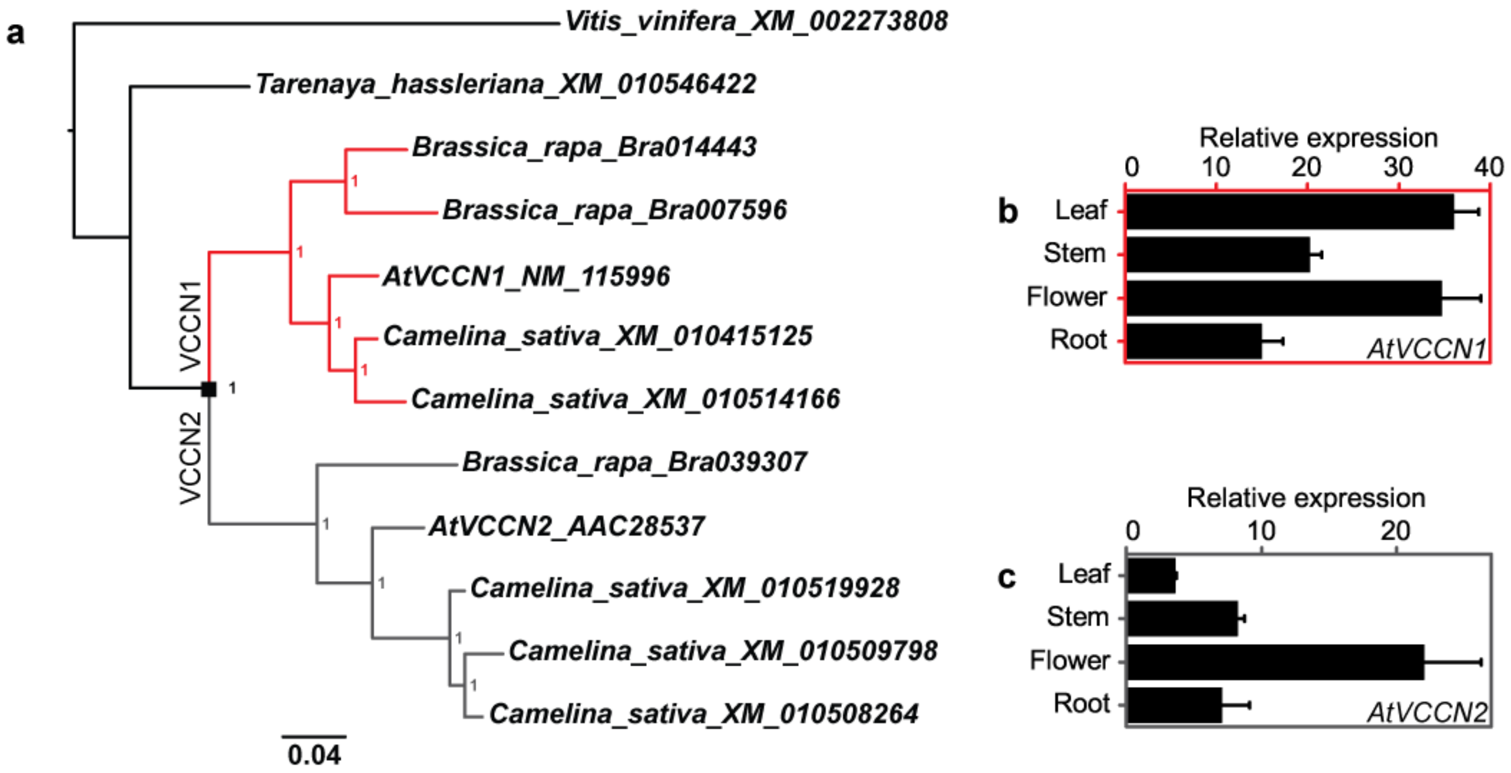

Supplementary Figure 3. VCCNs have diverged in plants and have distinct expression pattern in *Arabidopsis*. a, Maximum clade credibility tree of selected VCCN-related genes (DNA sequences) from a Bayesian reversible model jump MCMC analysis. The analysis is based on the MAFFT alignment, trimmed as described in Methods, and midpoint-rooted. Clade posterior probabilities >0.5 are shown to the right of branches. The gene duplication of the ancestral gene copy that gave rise to VCCN1 and VCCN2 lineages is marked with a black square. This event occurred between 24–40 Myr ago, *i.e.*, after the divergence of Cleomaceae from Brassicaceae but before the divergence of *Brassica* and *Arabidopsis*[2]. VCCN1 and VCCN2 lineages are shown as red and grey branches, respectively. The shared branch of the VCCN2 lineage (labelled) is under positive selection. The scale bar indicates substitutions per site. **b** and **c**, Quantitative analyses of *VCCN1* (**b**) and *VCCN2* (**c**) gene expression in *Arabidopsis*. Real time quantitative PCR was conducted using cDNA synthesized from RNA isolated from the rosette leaves, stems, flowers and roots of 7-week-old wild-type plants. Expression relative to the reference gene *PEX4* was calculated as $2^{-\Delta Cq}$. Values plotted are the mean ±s.e.m. (n=3).

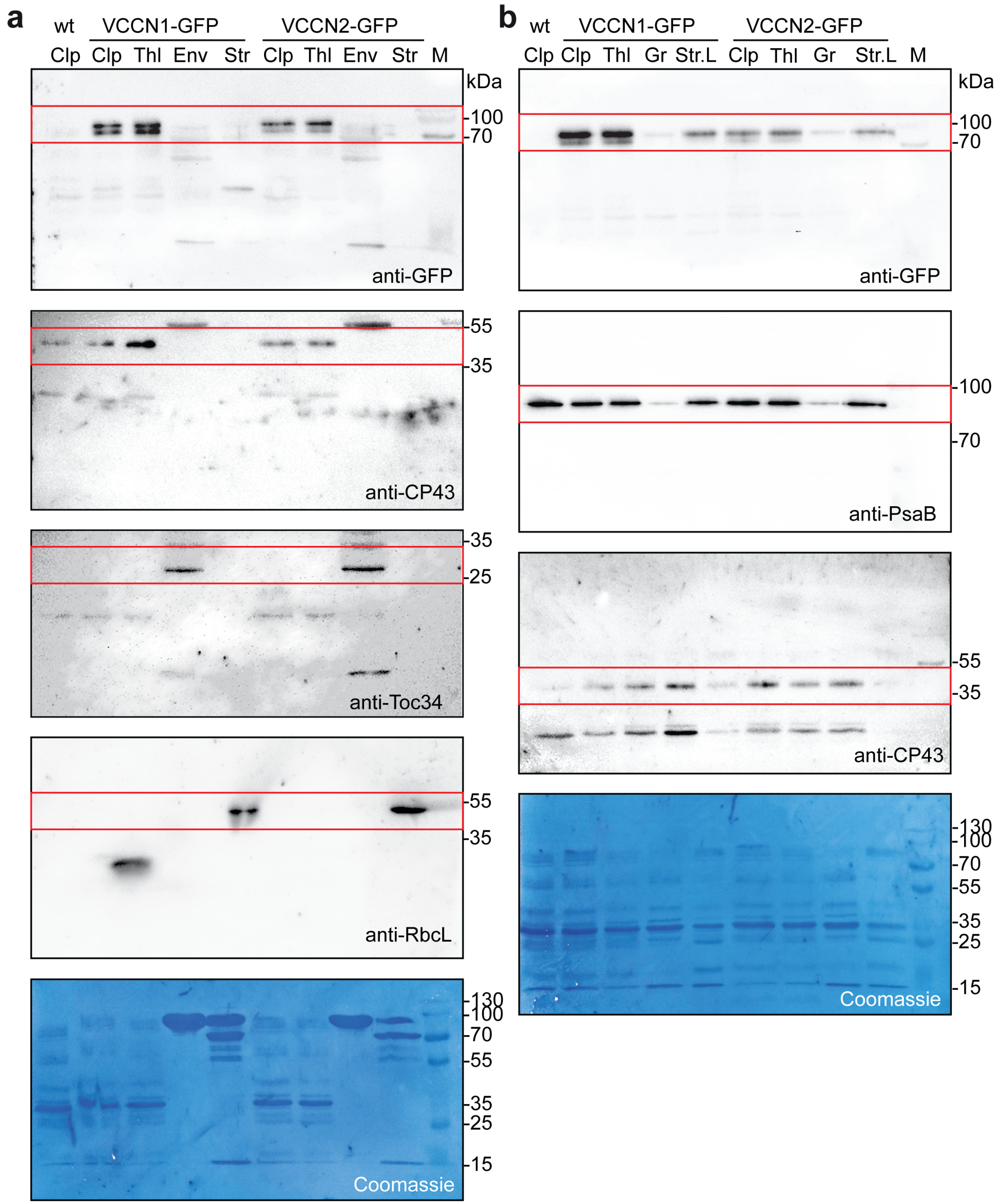

Supplementary Figure 4. Localization of VCCN1 and VCCN2 in *Arabidopsis* chloroplast (a) and thylakoid subfractions (b). The uncropped images of immunoblots corresponding to Fig. 1b,c are shown. The proteins from chloroplasts (Clp), envelope (Env), stroma (Str), thylakoids (Thl), grana (Gr), stroma lamellae (Str.L) and molecular weight markers (M) were separated by SDS-gel electrophoresis, electro-transferred to PVDF membranes and probed with the indicated antibodies. The Coomassie-stained membranes after protein transfer and immunoblotting are shown as loading controls.

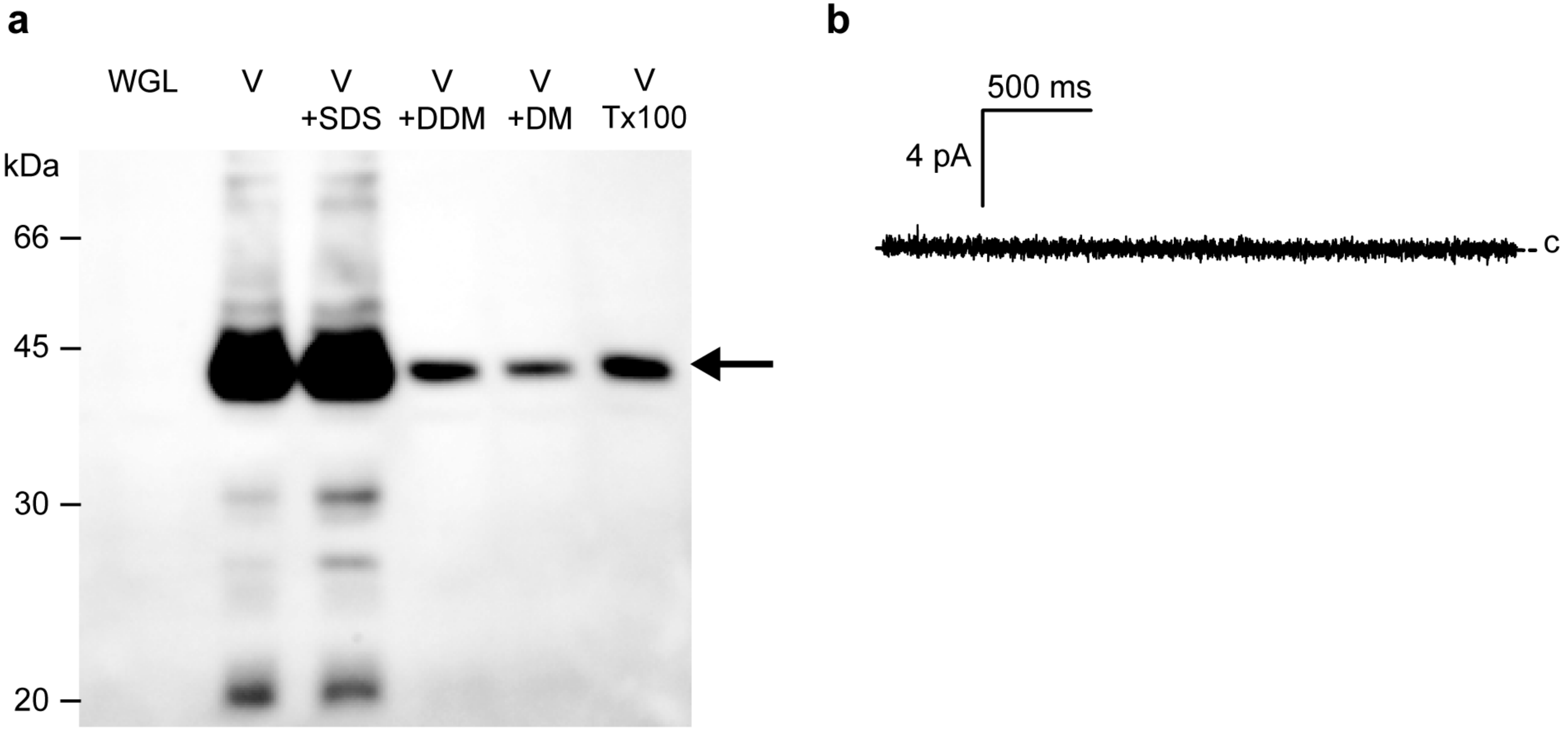

Supplementary Figure 5. Cell-free *in vitro* expression of AtVCCN1. a, Immunoblot showing a representative expression *in vitro*. Empty Wheat Germ Lysate (WGL) and lysate after expression of AtVCCN1 (V) were assayed with an anti-His-tag antibody to check protein expression. After expression, the protein was solubilized with different detergents at a final concentration of 2% (w/v) to ascertain optimal conditions: sodium dodecyl sulfate (SDS), dodecylmaltoside (DDM), decylmaltoside (DM), and Triton X-100 (Tx100), centrifuged, and the supernatants were loaded on the gel. The AtVCCN1 polypeptide band is indicated with an arrow. Electrophysiology measurements giving reproducible results were undertaken with the protein obtained in six different experiments using the expression kit and solubilized with 2% Tx100. Few microliters of the solubilized protein were added to the *cis* chamber. Control experiments (*n*=6) showed that the same volume of 2% Tx100 added to the membrane without protein did not alter membrane stability. **b**, Current trace recorded at -100 mV using the empty wheat germ lysate in which no exogenous protein has been transcribed and translated. The trace is representative of 15 experiments.

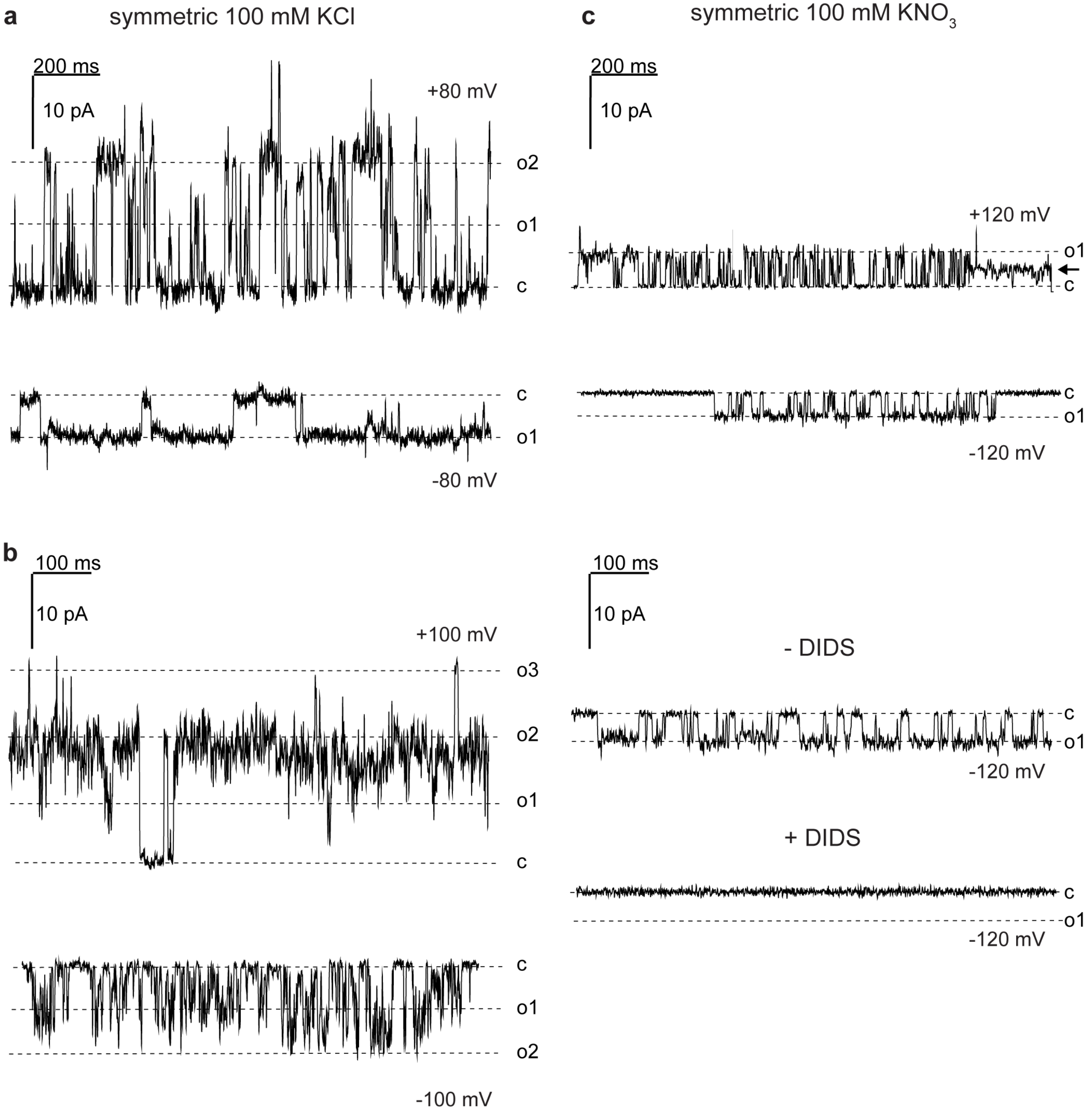

Supplementary Figure 6. Recombinant AtVCCN1 displays a voltage-sensitive anion channel activity in planar lipid bilayers. a-c, Representative current traces recorded in symmetric KCl (*n*=15) (**a** and **b**) or KNO_3 (*n*=26) (**c** *upper panel*) solutions (*cis* side: 100 mM, *trans* side: 100 mM) from different experiments with respect to those illustrated in Fig. 2a. Activity is shown at the indicated potentials (*cis* side) (c, closed state; o1, o2 and o3, open states). The arrow in **c** *upper panel* indicates a half-subconductance state. **c** *lower panel*, Current traces recorded before and after addition of 20 µM DIDS in 100 mM KNO_3, *n*=6; for amplitude histograms of the same experiment, see Supplementary Fig. 8e). The conductance values, the more prominent activity at positive voltages and sensitivity to DIDS are typical characteristics of the channel that can be observed in different experiments (see also Fig. 2 and Supplementary Fig. 10).

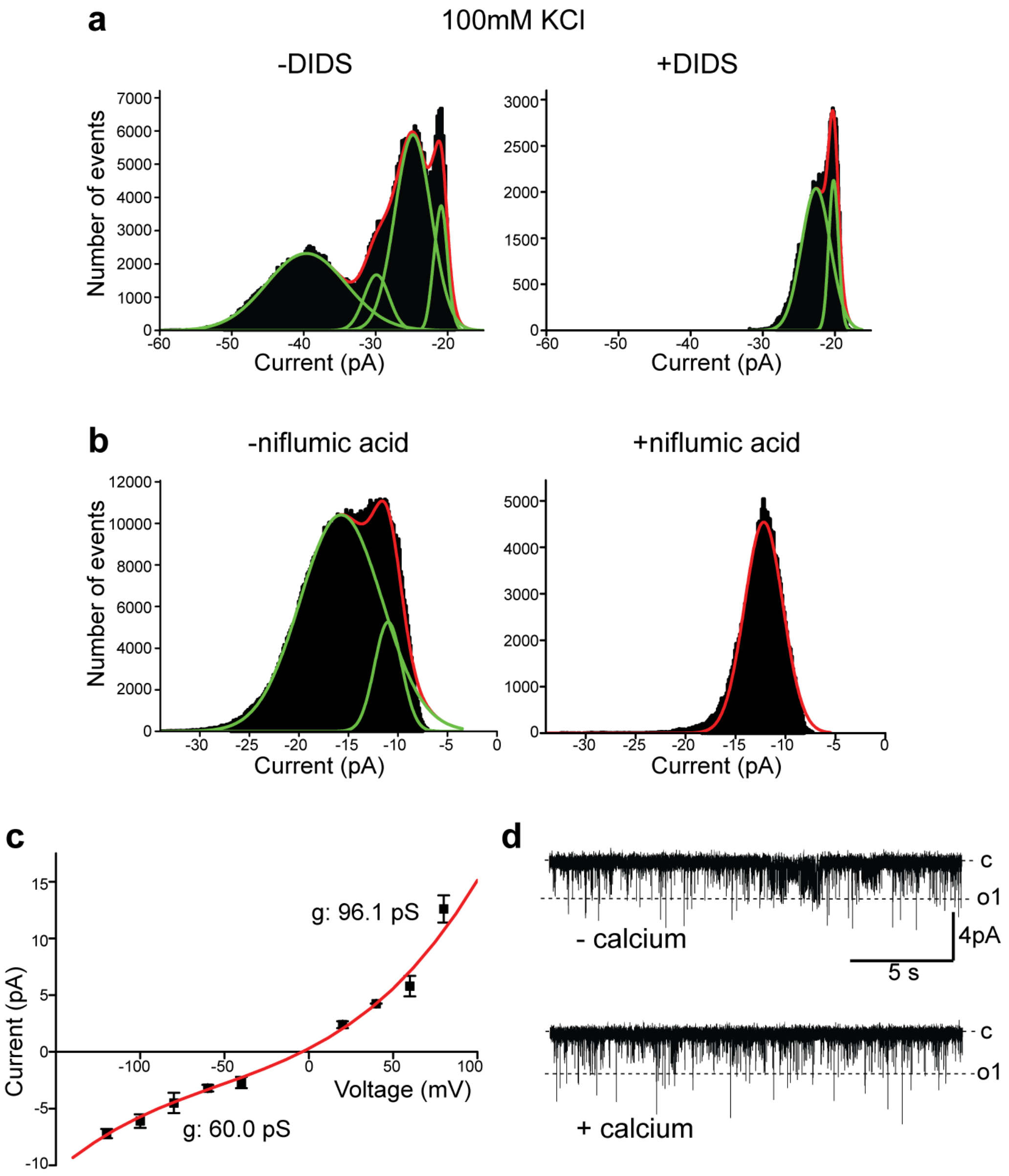

Supplementary Figure 7. Electrophysiology of AtVCCN1 in 100 mM KCl. a, Current amplitude histograms obtained in 100 mM KCl from the experiment shown in Fig. 2b (V*cis*=−100 mV) before (*left panel)* and after *(right panel)* addition of 20 μM DIDS to both sides of the membrane. **b,** Effect of 500 μM niflumic acid as evaluated by amplitude histograms (representative of two experiments). The presence of the open-state and closed-state levels is shown by green and red Gaussian curves, respectively. Amplitude histograms in **a**–**b** were obtained using the PCLAMP8 program. Gaussian fits of multi-peak histograms (red) and AtVCCN1 current levels (green) were obtained using the Origin 6.1 software. Leak current was not subtracted in any of the experiments. **c,** AtVCCN1 displayed slightly rectifying activity with a conductance of 96.1±5.0 pS at positive potentials and 60.0±8.2 pS chord conductance at negative voltages as observed from the current–voltage relationship. Reported current values are the mean ±s.d. obtained from amplitude histogram fittings at the different voltages from five different experiments. In the range between +60 mV and –60 mV, the channel showed an ohmic behavior. **d,** Lack of effect on channel activity by addition of 1 mM $CaCl_2$ to both sides of the membrane (representative of three experiments) (c, closed state; o1, open state). V*cis*=−60 mV.

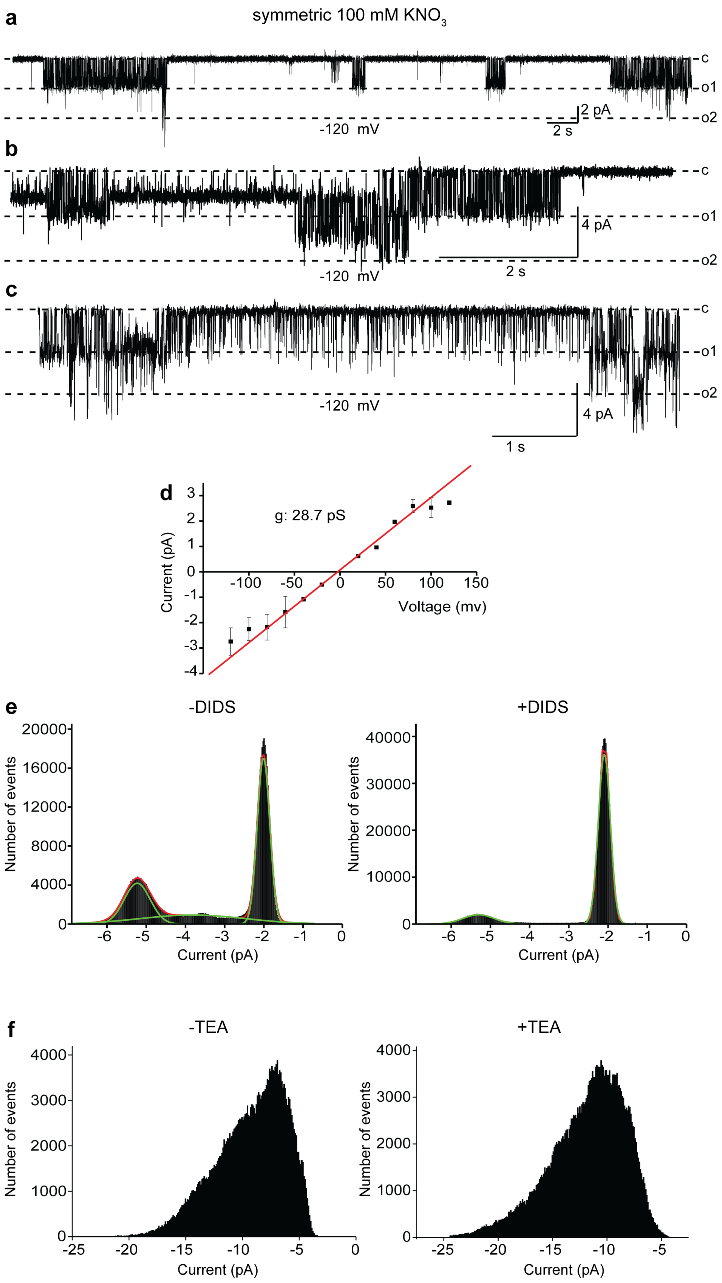

Supplementary Figure 8. Electrophysiology of AtVCCN1 in 100 mM KNO_3. a-c, Current traces recorded at indicated voltages from different experiments than those shown in Fig. 2b illustrate the typical kinetic behavior of the channel (observed also in 100 mM KCl): (**a**) burst-like opening, (**b**) cooperative gating, and (**c**) change from slower kinetic to fast gating (c, closed state; o1, and o2, open states). **d**, AtVCCN1 displayed a chord conductance of 28.7±12.2 pS in symmetrical 100 mM KNO_3. Reported current values are the mean ±s.d. obtained from three independent experiments. Where not visible, error bars are smaller than the symbols. **e,** Current amplitude histograms obtained from the experiment in 100 mM KNO_3 shown in Supplementary Fig. 6c (V_{cis}=−120 mV) before (*left panel*) and after (*right panel*) addition of 20 μM DIDS to both sides of the membrane. Gaussian fits of multi-peak histograms (red) and AtVCCN1 current levels (green) were obtained using the Origin 6.1 software. **f**, Current amplitude histograms obtained before and after addition of 10 mM TEA^+. No effect was observed. Amplitude histograms in e-f were obtained using the PCLAMP8 program. Fitting was obtained using Origin 6.1 software.

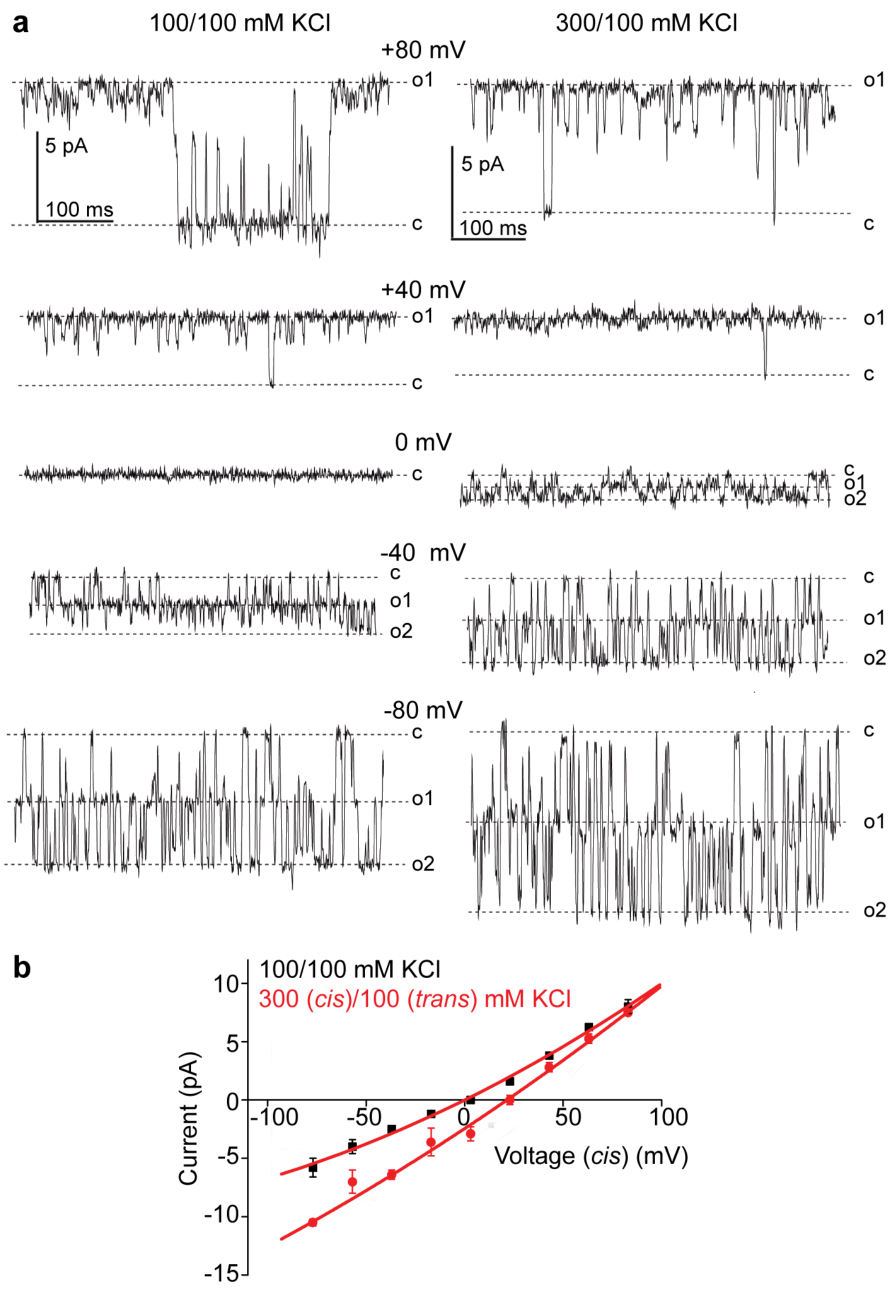

Supplementary Figure 9. Cation versus anion selectivity of AtVCCN1. a, Current traces recorded at indicated voltages (*cis* side) from one representative experiment under symmetric ionic conditions (100 mM KCl) *(left panels)* and under ionic gradient (300 mM KCl *cis* side, 100 mM KCl *trans* side) *(right panels)*. In this particular experiment, the half-conductance substate (o1) was prevalently observed at negative voltages. **b,** Current-voltage relationship from the same experiment shown in **a**. Where not visible, error bars are smaller than the symbols. The reversal potential (Erev) under asymmetric ionic conditions was 19.0 mV yielding a permeability ratio of $P_{Cl}^{-}:P_{K}^{+}$=1:0.17. In other two experiments Erev values of 17.2 and 20.2 mV were obtained, yielding thus an average value of 18.8±1.5 mV (± indicates s.d.), corresponding to $P_{Cl}^{-}:P_{K}^{+}$ = 1: 0.17 ±0.03.

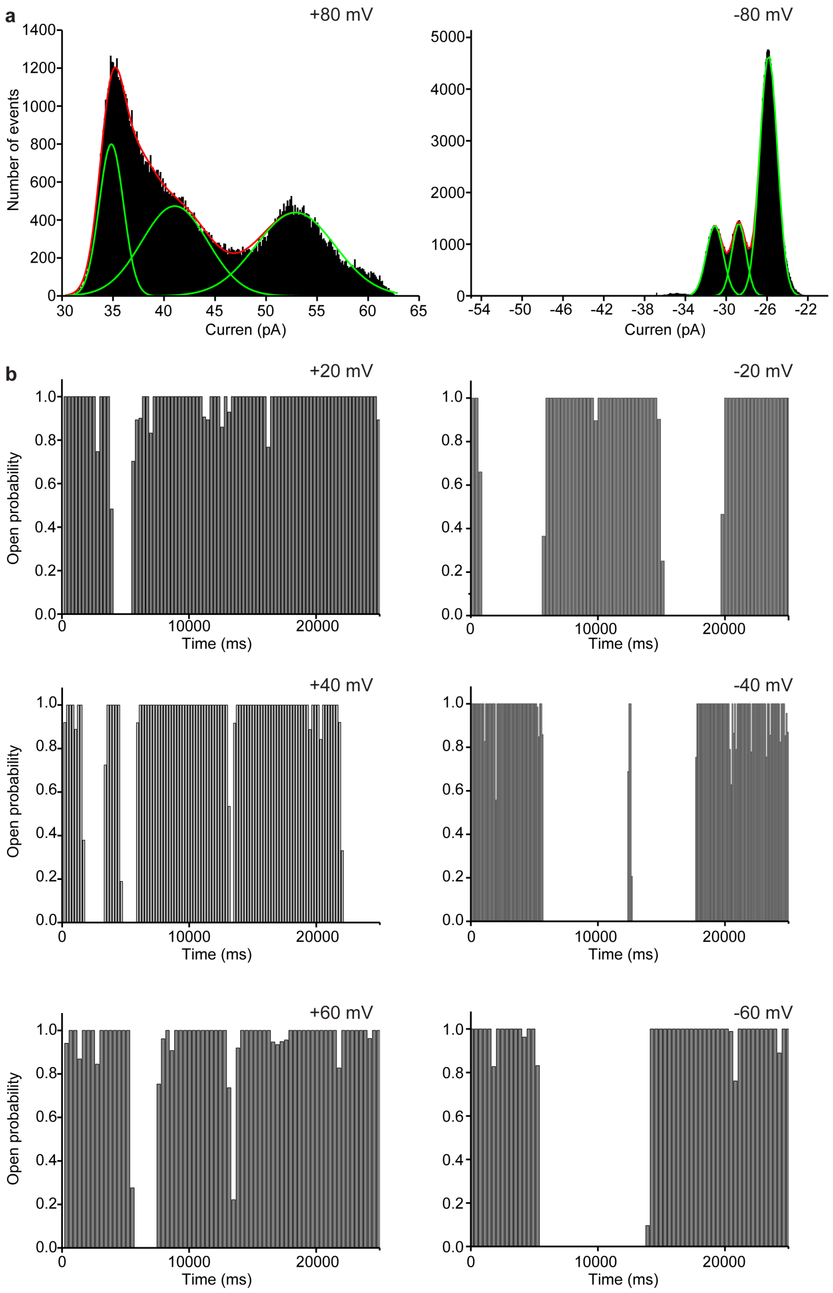

Supplementary Figure 10. Voltage dependence of AtVCCN1 studied in 100 mM KCl. a, Amplitude histograms obtained at −80 and +80 mV applied potentials (*cis* side) from 30 s current traces (experiment shown in Supplementary Fig. 6a). Continuous, flickering activity was observed in nearly all experiments (see also Supplementary Fig. 6b), whereas burst-like opening (a train of transitions to open state with long intervals of closure) was especially prevalent at negative voltages (see also **b** and Supplementary Fig. 8a). Overall, the activity was higher at positive potentials. **b,** Open probability density histograms for open channel level. Open probability as a function of time was monitored and measured for the indicated times at the indicated voltages from another experiment where gating kinetics were relatively slow. The open probability (P_o) was calculated for each time segment (automatically binned by PSTAT) as the mean integral current divided by the maximum current which could be attained if the channels present in the membrane were continuously open (P_o=1). Analysis has been performed using the Fetchan and PSTAT programs of PCLAMP8.0 software according to the manual. The P_o values were the following: 0.94 at +20 mV and 0.64 at -20 mV, 0.83 at +40 mV and 0.52 and −40 mV, 0.88 at +60 mV and 0.60 at −60 mV. Burst-like behavior (long periods without any channel activity, i.e. P_o=0) is especially evident at negative applied voltages (see histograms in **b**). P_o values of 0.61 and 0.39 have been obtained from another experiment at +40 and −40 mV, respectively.

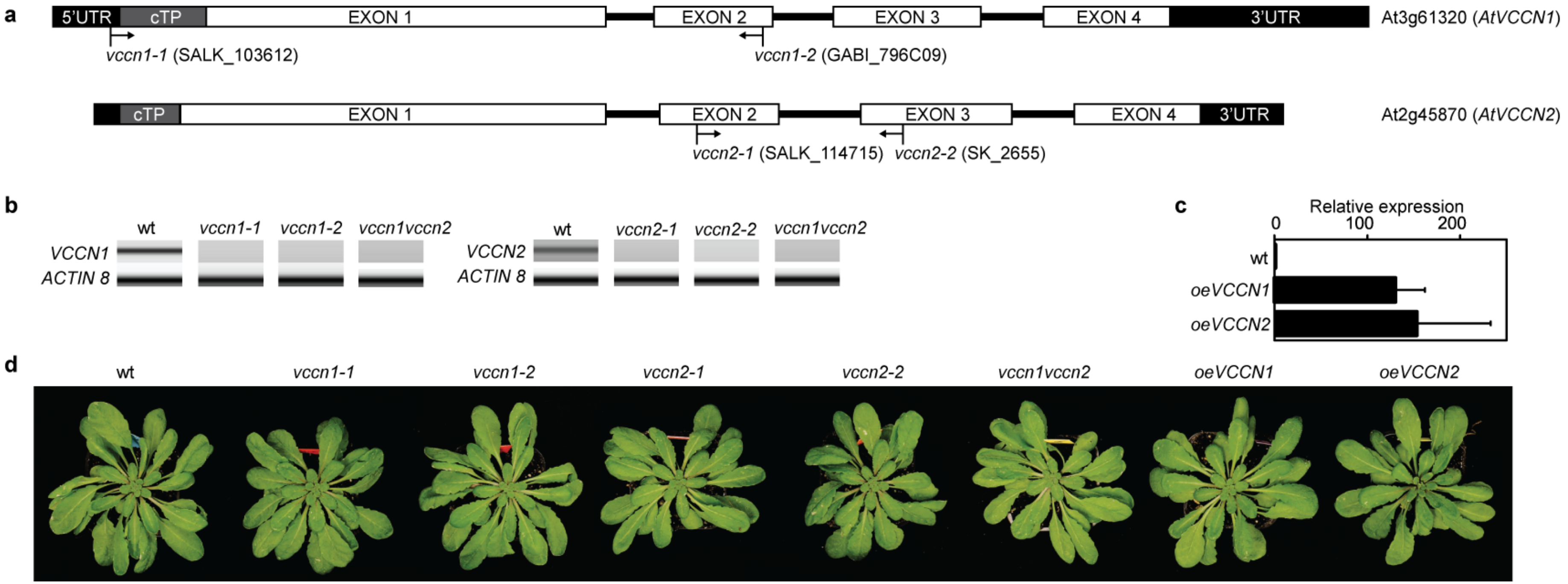

Supplementary Figure 11. Genotype and phenotype of the mutants used in this work. a, Structure of *AtVCCN* genes with site and orientation of T-DNA insertions in the *vccn* mutants. The double *vccn1vccn2* line was obtained by crossing *vccn1-1* and *vccn2-1*. **b**, RT-PCR analyses were undertaken on total leaf RNA from wild-type (wt) and mutant plants with *AtVCCN* specific primers and primers to the reference gene *ACTIN8* as positive control. Absence of the corresponding transcript in the *vccn* lines indicates that they are knockout mutants. **c**, Transcript levels of the *AtVCCN1* and *AtVCCN2* genes in wt and overexpressing mutant lines. Real-time quantitative PCR was conducted using cDNA synthesized from RNA isolated from rosette leaves of 7-week-old wt plants, *oeVCCN1* and *oeVCCN2* lines. Expression relative to the reference gene *PEX4* was calculated as $2^{-\Delta Cq}$, and normalized to wt transcription level of the respective gene. Values plotted are means ±s.e.m. (*n*=3). **d**, Representative photo of mutant lines grown under a light regime of 8h/16h light/dark cycle and at a light intensity of 150 μmol photons m^{-2} s^{-1} shows no difference in growth with respect to wt.

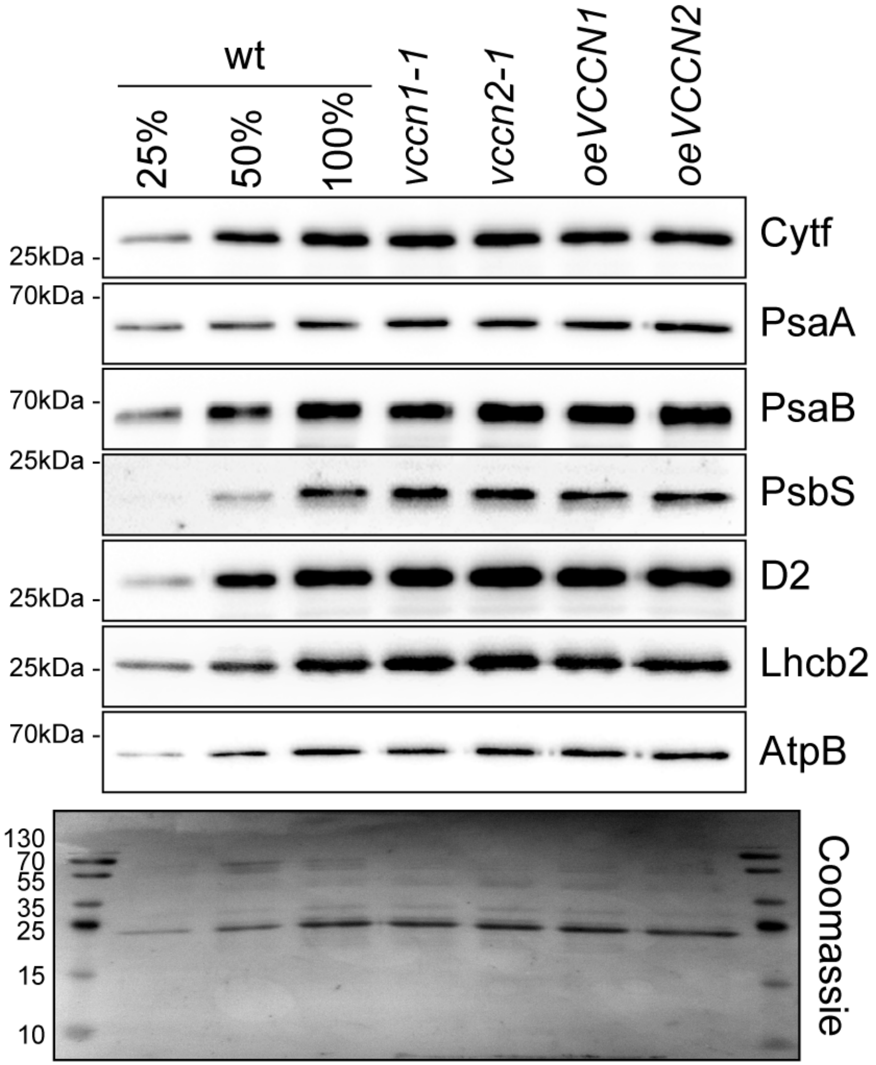

Supplementary Figure 12. Levels of photosynthetic complexes. Chloroplast protein extracts were prepared from wild type (wt), *vccn1-1*, *vccn2-1*, *oeVCCN1* and *oeVCCN2* mutant plants. The proteins were separated by SDS-gel electrophoresis, electro-transferred to PVDF membranes and probed with antibodies against marker proteins for photosystem II (D2, Lhcb2, PsbS), photosystem I (PsaA, PsaB), cytochrome b_6f (Cytf), and ATP synthase (AtpB). As a loading control, a Coomassie-stained membrane after protein transfer and immunoblotting is shown. Uncropped versions of the immunoblots are shown in Supplementary Fig. 22.

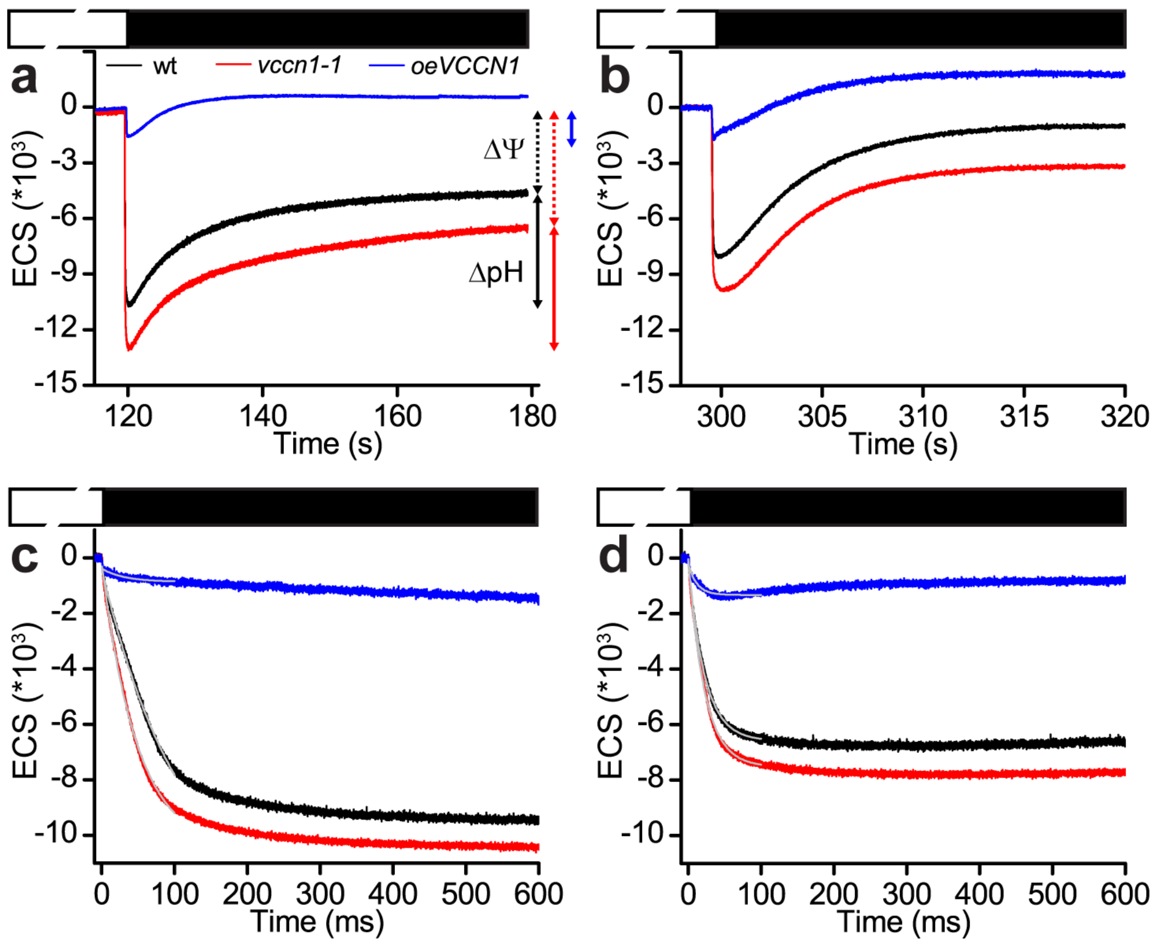

Supplementary Figure 13. Electrochromic shift (ECS) decay kinetics after different illumination periods. Plants were dark-adapted for 30 min and then illuminated with 650 µmol photons m^{-2} s^{-1} (white bar) for 2 min (**a**), or 5 min (**b**) after which illumination was stopped (black bar) to record ECS decay in darkness. ΔpH and ΔΨ contribution to PMF were determined as indicated with dotted (ΔΨ) and full double-headed arrows (ΔpH). To determine the g_H^+ parameter, ECS decay kinetics were recorded during 600 ms dark intervals. Examples of averaged curves after 1 min (**c**) or 9 min (**d**) of illumination are shown (*n*=5). The ECS decay of the first 100 ms was fitted with a single exponential function (shown with grey lines) to calculate $g_H^+(s^{-1})$ =1/time constant for decay.

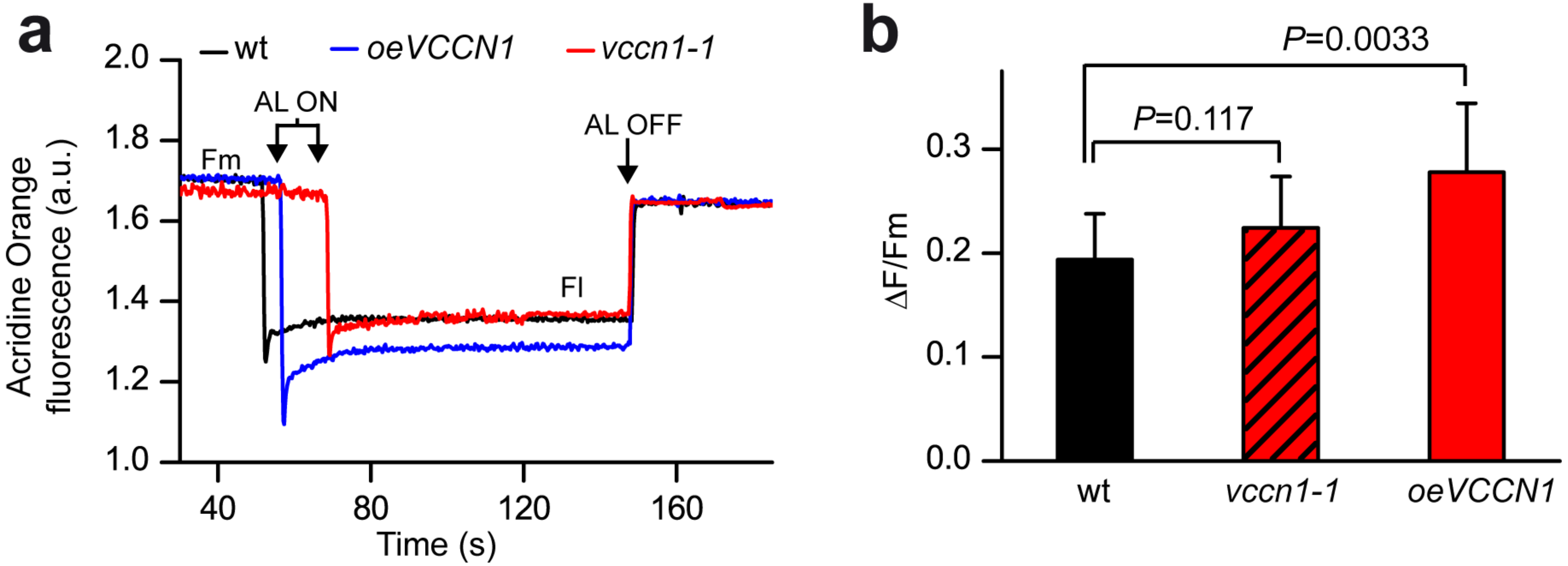

Supplementary Figure 14. Thylakoid lumen acidification assessed by acridine orange (AO) fluorescence. Chloroplasts isolated from leaves and purified by Percoll gradient were preincubated at 10 µg Chl ml^{-1} in the purification medium with 1 µM AO for 20 min in darkness prior to measurement. **a,** Representative AO fluorescence traces obtained from wt and mutant plants. Downward deflections correspond to quenching of the AO fluorescence attributable to its protonation within the thylakoid lumen following application of actinic light (AL, 700 µmol photons m^{-2} s^{-1}), whereas upward deflections indicate dissipation of ΔpH when the light is switched off. **b**, ΔF/Fm values for isolated chloroplasts from wild type (wt) (*n*=18), *vccn1-1* (*n*=9) and *oeVCCN1* (*n*=11) mutants. AO fluorescence values were measured in the dark (Fm) and 100 s after illumination in the steady state (Fl). ΔF was calculated as Fm-Fl. The mean ±s.d. is plotted and the *P*-values of Student *t*-test are shown. ΔF/F ratio decreased to 0.143±0.048 in wt upon addition of 0.5 µM FCCP protonophore indicating that the light-induced changes in the AO fluorescence reflect changes in H^+ concentration. The ΔF/Fm data indicate a significantly more acidic lumenal pH in the light in *oeVCCN1* than in wt, supporting the higher PMF partitioning to ΔpH in this line (Fig. 3b). The lumenal pH was similar to wt in *vccn1-1* although lower PMF partitioning to ΔpH was indicated by ECS measurements. The explanation may reside in that AO fluorescence is a semi-quantitative method for lumenal acidification employed in isolated systems, whereas ECS measures PMF partitioning to ΔpH in intact leaves.

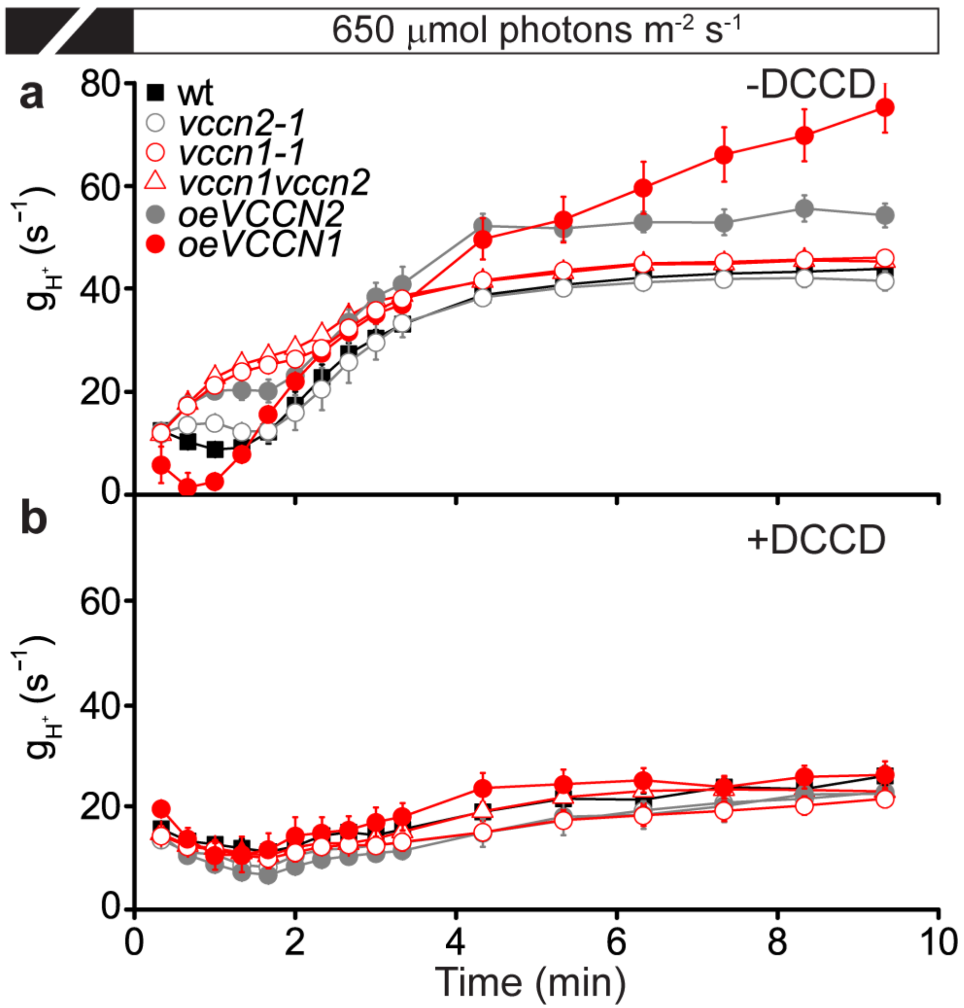

Supplementary Figure 15. Effect of DCCD on H^+ efflux through ATP synthase. Leaf discs from wild-type (wt) plants and mutants were incubated for 10 min without (**a**) or with 5 mM DCCD (**b**) and then illuminated for 10 min at 650 μmol photons m^{-2} s^{-1}. Electrochromic shift (ECS) was recorded with brief intervals of darkness during illumination. The H^+ efflux through ATP synthase ($g_H^{\ +}$) was calculated from ECS decay kinetics as described in Methods. The means ±s.e.m. are plotted (*n*=6-8 in **a**, and 5-6 in **b**). Where not visible, error bars are smaller than the symbols.

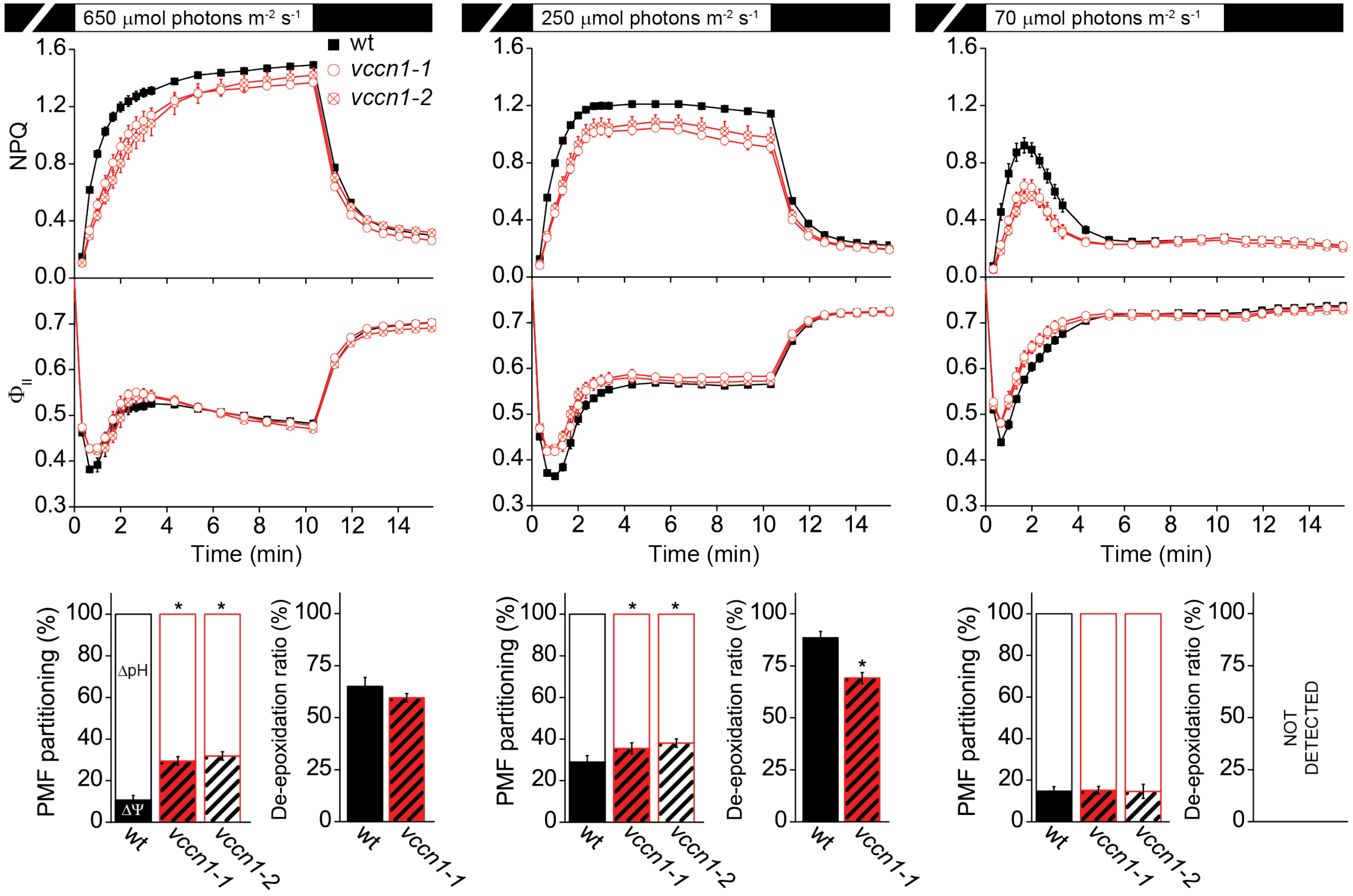

Supplementary Figure 16. Non-photochemical quenching (NPQ), photosystem II quantum yield (Φ_{II}), proton motive force (PMF) partitioning and de-epoxidation ratio at three light intensities. Slow kinetics of NPQ induction were recorded on 30 min dark-adapted wild type (wt) plants, *vccn1-1* and *vccn1-2* mutants during 10 min of illumination at indicated light intensities followed by 5 min of relaxation in darkness. The Φ_{II} parameter was calculated from the same experiment as NPQ. PMF partitioning was determined after 10 min of illumination from ECS decay kinetics. Leaf samples were collected at the end of the light period and analyzed by high-performance liquid chromatography for the content of violaxanthin, antheraxanthin and zeaxanthin. The de-epoxidation ratio was calculated as (antheraxanthin+zeaxanthin)/(violaxanthin+antheraxanthin+zeaxanthin) and expressed as a percentage. Means ±s.e.m. are plotted (NPQ and PMF measurements n=5 and pigment analyses n=10). Where not visible, error bars are smaller than the symbols. Asterisks in the bar plots indicate a statistically significant difference between wt and mutants (Student's t-test, P<0.05).

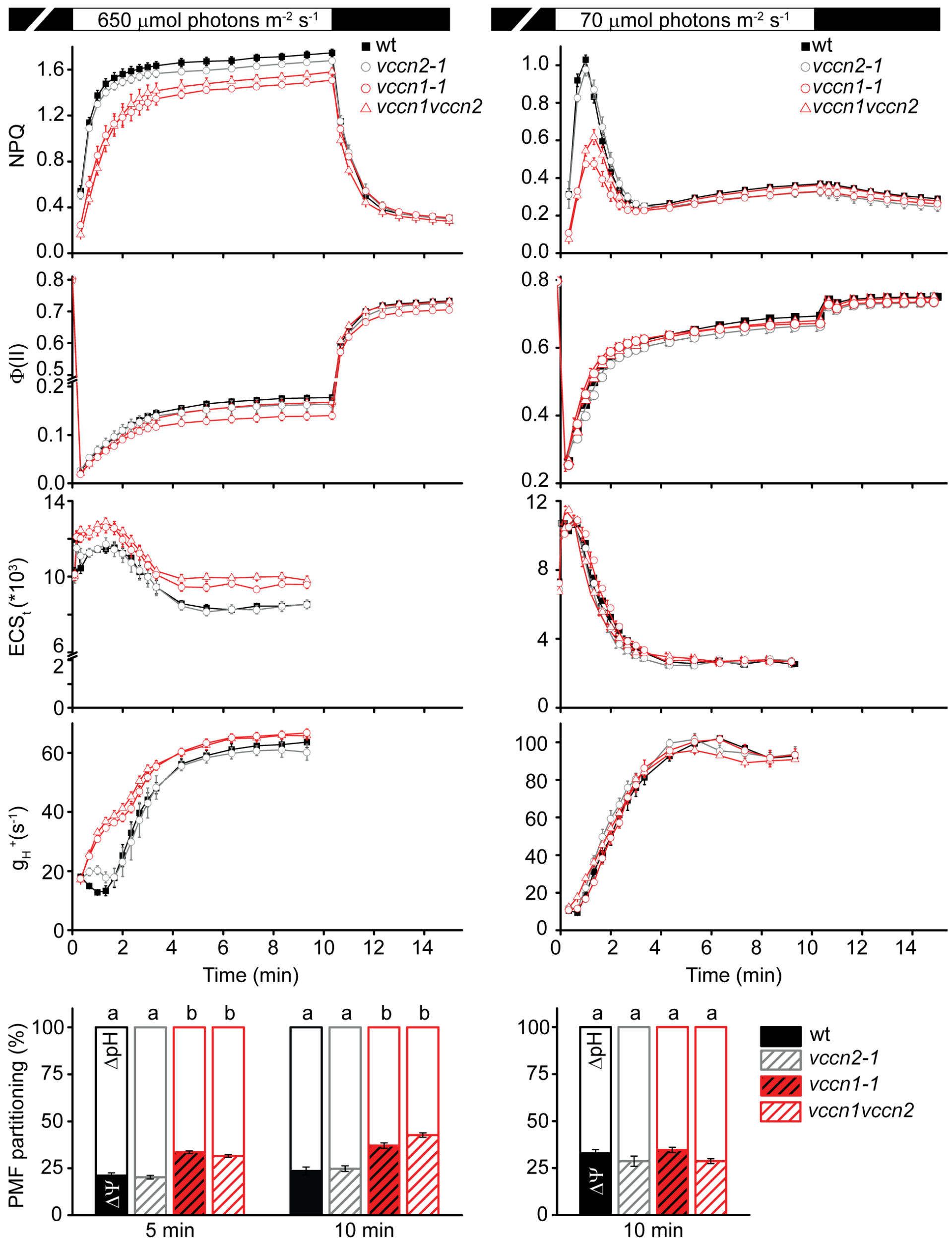

Supplementary Figure 17. Non-photochemical quenching (NPQ), photosystem II quantum yield (Φ_{II}), proton motive force (PMF) size, H$^+$ conductivity through ATP synthase (g_H^+) and PMF partitioning at two light intensities. Slow kinetics of NPQ induction were recorded on 30 min dark-adapted wild type (wt) plants, *vccn1-1*, *vccn2-1* and *vccn1-1vccn2-1* mutants during 10 min of illumination at indicated light intensities followed by 5 min of relaxation in darkness. The Φ_{II} parameter was calculated from the same experiment as NPQ. PMF size was measured during illumination as the total electrochromic shift (ECS_t), whereas the g_H^+ and PMF partitioning into ΔpH and ΔΨ were determined from ECS decay kinetics. Means ±s.e.m. are plotted (*n*=5). Where not visible, error bars are smaller than the symbols. Different letters in the bar plots indicate a statistically significant difference between the genotypes (Student's *t*-test, *P*<0.05).

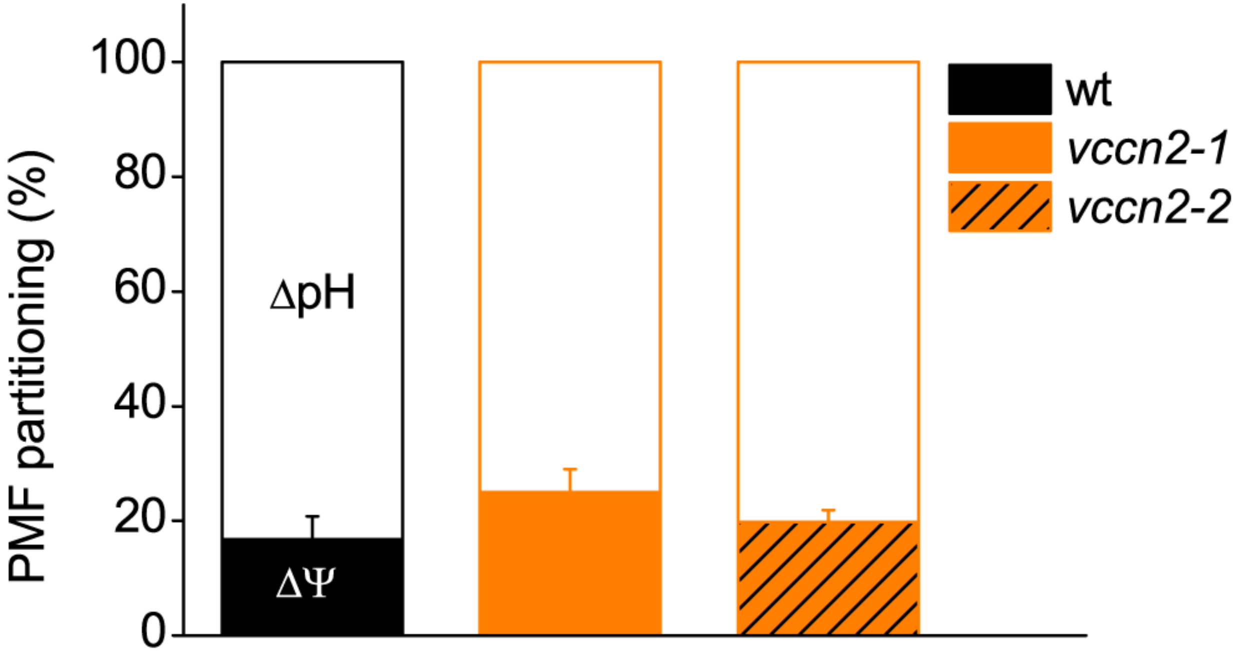

Supplementary Figure 18. Composition of proton motive force (PMF) in *vccn2* mutants. Plants were dark-adapted for 30 min and then illuminated for 10 min with 1,300 μmol photons m^{-2} s^{-1}. Following illumination, kinetics of dark relaxation of the electrochromic shift signal were recorded and deconvoluted to determine ΔΨ and ΔpH. Means ±s.e.m. are plotted (*n*=5). No significant differences in PMF partitioning were observed between wild type (wt) and *vccn2* mutants (Student's *t*-test, *P*>0.05).

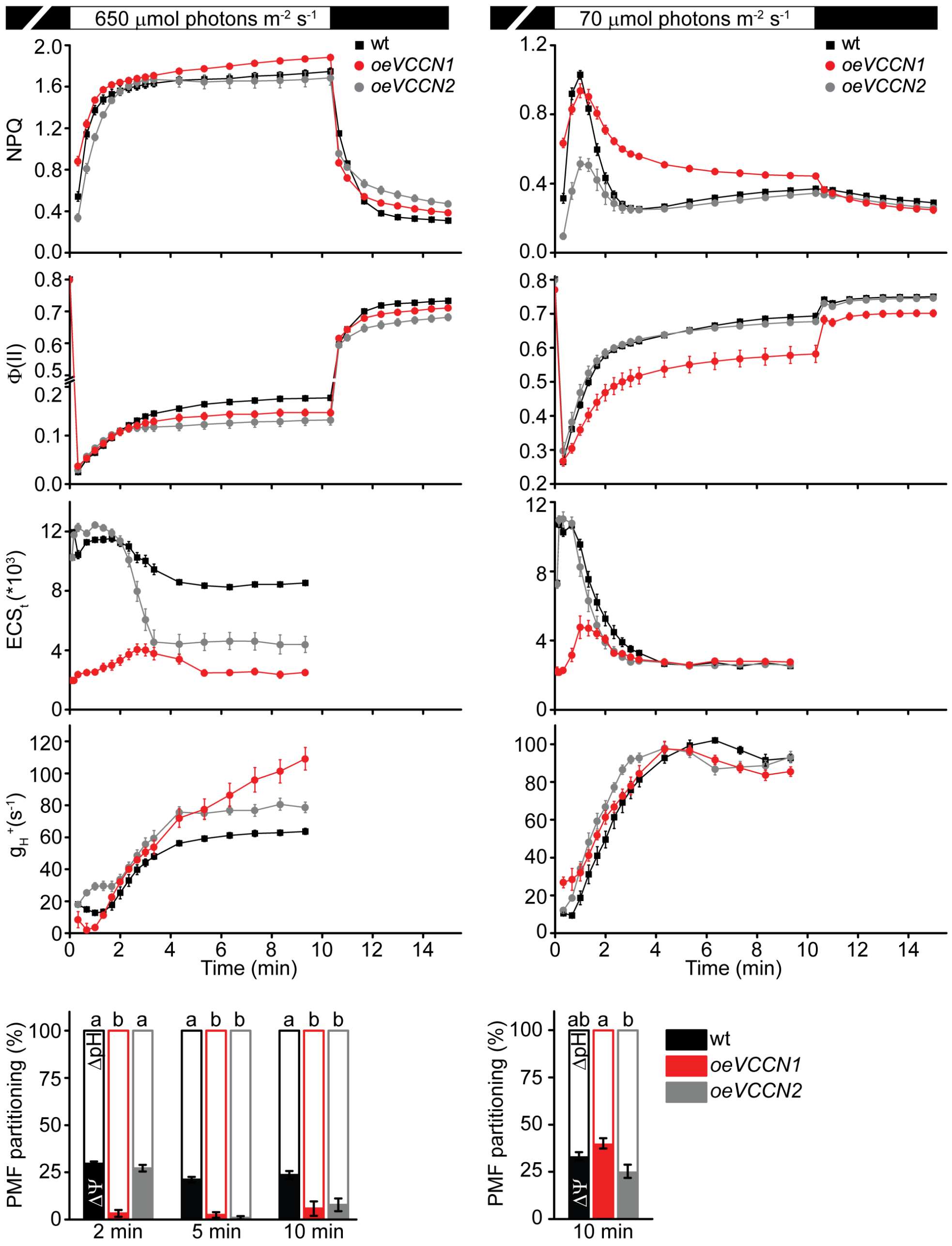

Supplementary Figure 19. Non-photochemical quenching (NPQ), photosystem II quantum yield (Φ_{II}), proton motive force (PMF) size, H^+ conductivity through ATP synthase (g_H^+), and proton motive force (PMF) partitioning at two light intensities. Slow kinetics of NPQ induction were recorded on 30 min dark-adapted wild type (wt) plants, *oeVCCN1* and *oeVCCN2* mutants during 10 min of illumination at indicated light intensities followed by 5 min of relaxation in darkness. The Φ_{II} parameter was calculated from the same experiment as NPQ. PMF size was measured during illumination as the total electrochromic shift (ECS_t), whereas the g_H^+ and PMF partitioning into ΔpH and ΔΨ were determined from ECS decay kinetics. Means ±s.e.m. are plotted (*n*=5). Where not visible, error bars are smaller than the symbols. Different letters in the bar plots indicate a statistically significant difference between the genotypes (Student's *t*-test, $P<0.05$).

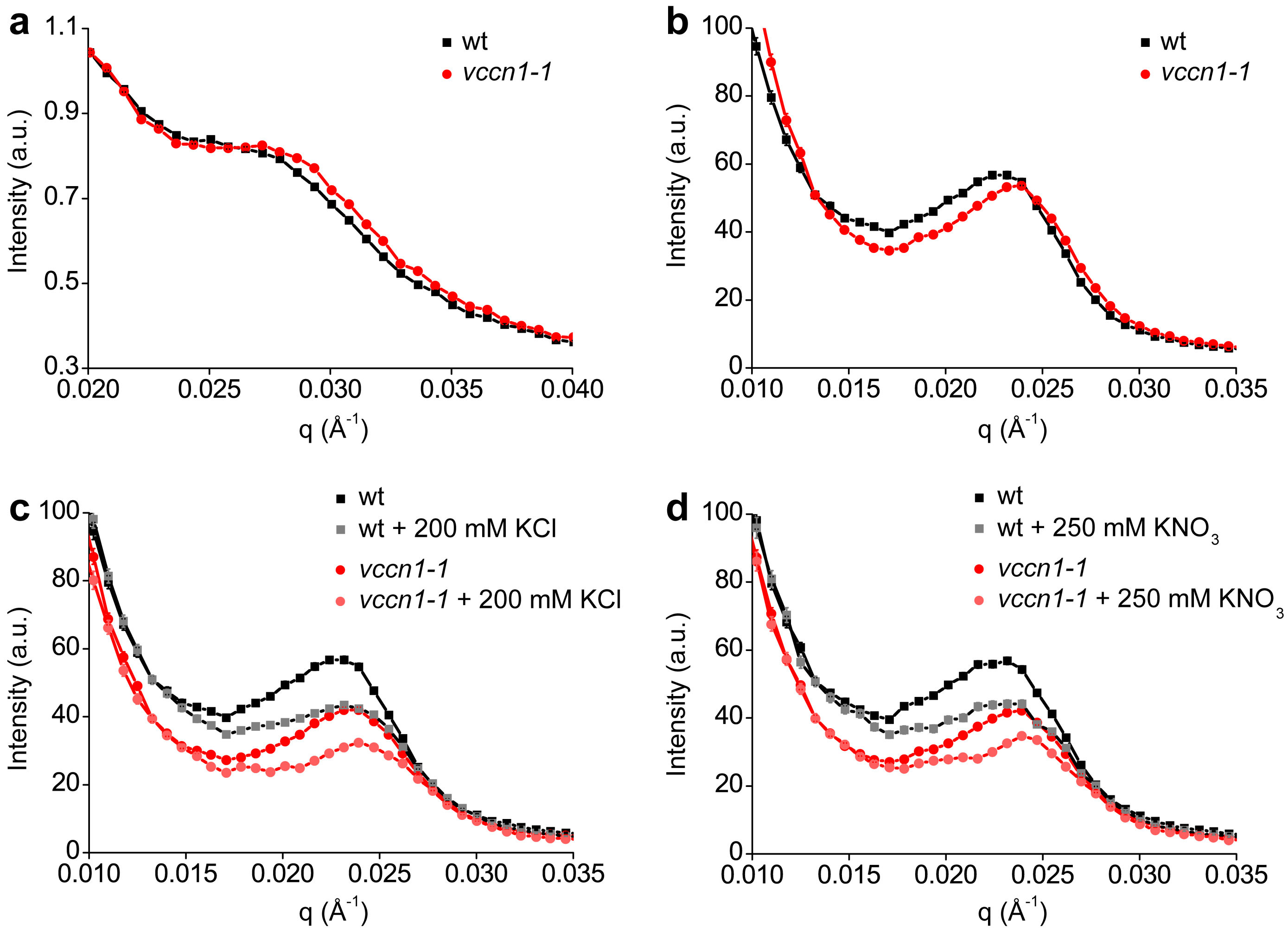

Supplementary Figure 20. Radially averaged small-angle neutron scattering (SANS) curves of D_2O-infiltrated detached leaves (a), and magnetically aligned untreated, KCl- or KNO_3-treated thylakoid membranes (b-d). The *vccn1-1* curve is normalized to the wild-type (wt) curve at q values of 0.020 $Å^{-1}$ in **a** and of 0.0133 $Å^{-1}$ in **b**. Curves for salt-treated thylakoids are normalized to the curve of the corresponding untreated sample at 0.0133 $Å^{-1}$ (**c-d**). For further details, see Methods. Possible kinetic differences in the salt-induced changes could not be resolved because steady state SANS profiles were reached in ~90–120 s, whereas salt administration took 15–30 s.

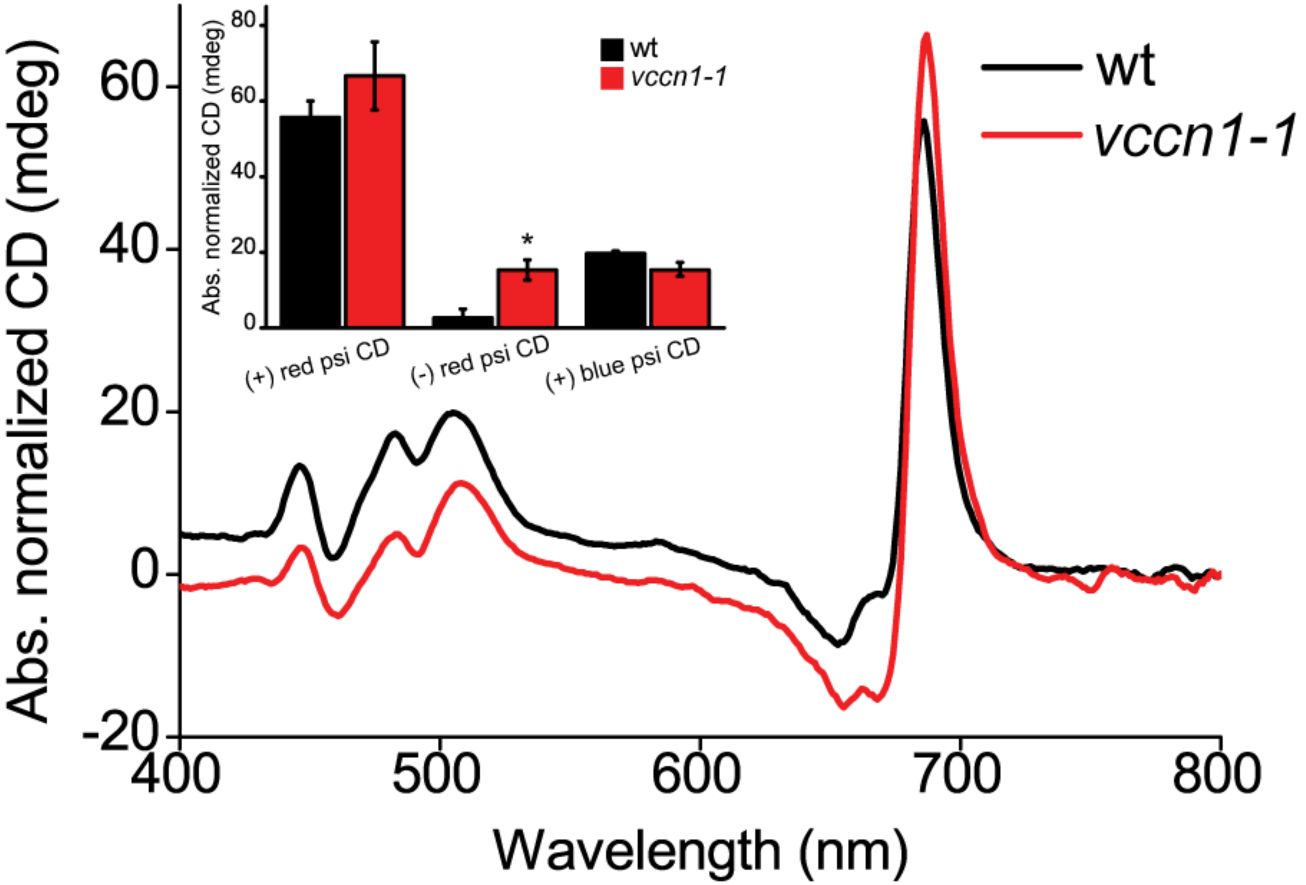

Supplementary Figure 21. Representative circular dichroism (CD) spectra of H_2O-infiltrated detached leaves. Insets, amplitudes of psi-type CD bands at around (+)505 nm (+blue), (–)675 nm (-red) and (+)690 nm (+red), with reference wavelengths at 550, 600 and 750 nm, respectively. These bands originate from a long-range-chiral organization of the pigment-protein complexes in the grana. The significantly increased amplitude of the (-)675 nm band indicates tighter stacking of the thylakoid membranes. The measurements were carried out on a Jasco J-815 spectropolarimeter; before averaging the psi-type amplitudes, the spectra were normalized to the red-most absorption peak of the samples and corrected for baseline distortions. Plotted data are means ± s.e.m. (n=3 leaves collected from different plants). Asterisks indicate significant differences between wild type (wt) and *vccn1-1* (Student t-test, P<0.05).

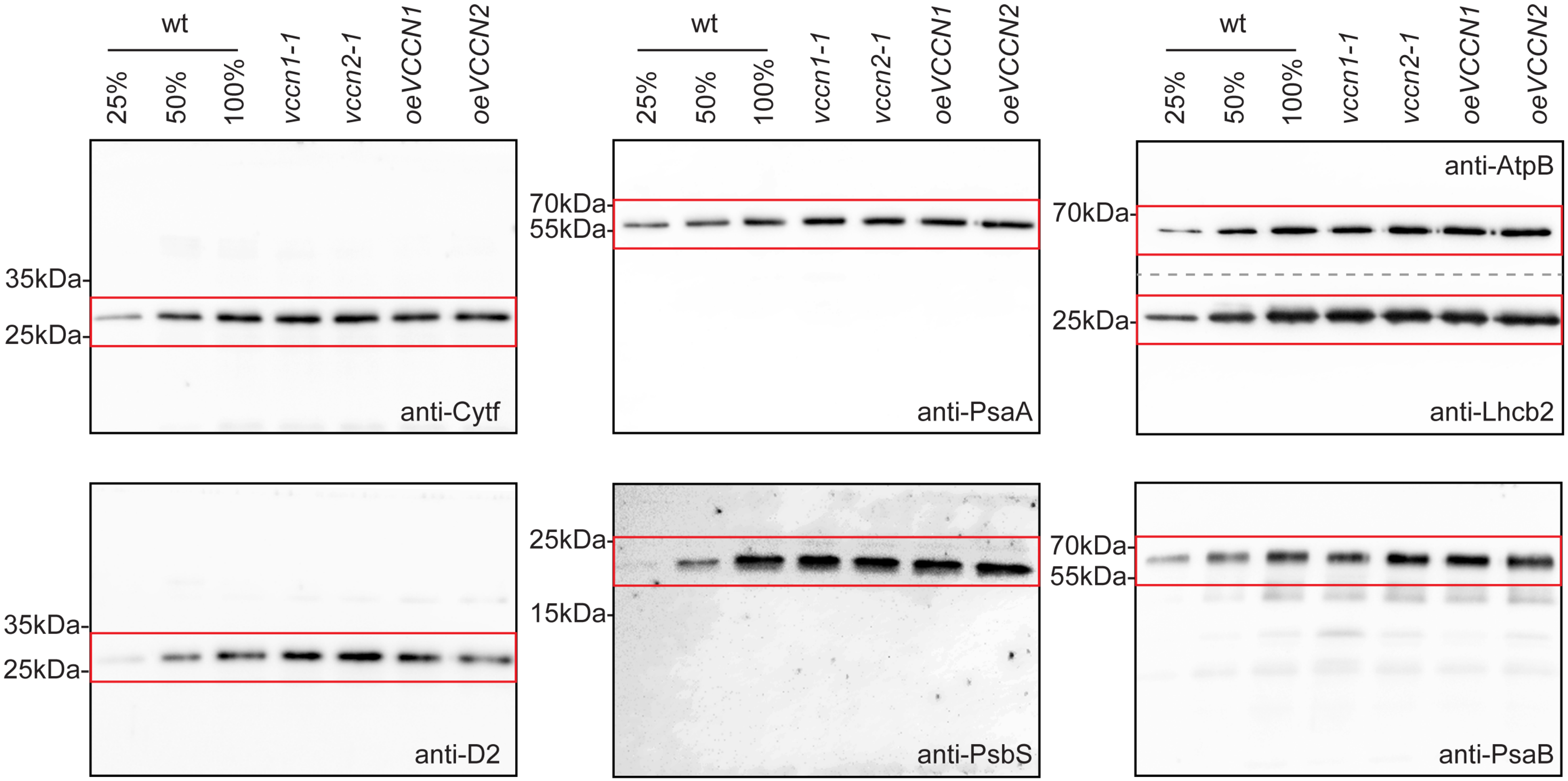

Supplementary Figure 22. Uncropped versions of the immunoblots shown in Supplementary Figure 12. The chloroplast proteins were separated by SDS-gel electrophoresis, electro-transferred to PVDF membranes and probed with antibodies against marker proteins for photosystem II (D2, Lhcb2, PsbS), photosystem I (PsaA, PsaB), cytochrome b_6f (Cytf), and ATP synthase (AtpB). Dashed grey line indicates cutting of the PVDF membrane before incubation with the indicated antibodies.

Supplementary Table 1. Shoot biomass and photosynthetic pigment content. Shoot biomass of 7–8-week-old wild-type (wt) plants and *vccn* mutants was determined in terms of fresh and dry weight. Photosynthetic pigments (chlorophylls and carotenoids) were extracted with ethanol from leaves of 16-h dark-adapted plants. Data are means ±s.e.m. (n=10 plants). There was no significant difference in any of the studied parameters between wt and the *vccn* mutants (Student t-test, P>0.05).

Parameter	wt	*vccn1-1*	*vccn1-2*	*vccn2-1*	*vccn2-2*	*vccn1vccn2*	*oeVCCN1*	*oeVCCN2*
Fresh weight (g)	3.60±0.13	3.44±0.27	3.69±0.12	3.50±0.20	3.26±0.27	3.58±0.20	3.22±0.14	3.41±0.36
Dry weight (g)	0.30±0.01	0.27±0.02	0.28±0.01	0.27±0.02	0.26±0.02	0.31±0.01	0.27±0.01	0.29±0.03
Chl *a* (µg cm^{-2})	12.72±0.42	12.65±0.30	12.95±0.44	12.99±0.16	13.85±0.26	13.74±0.55	12.32±1.42	12.85±0.29
Chl *b* (µg cm^{-2})	3.26±0.11	3.13±0.08	3.18±0.13	3.34±0.03	3.55±0.10	3.14±0.01	3.23±0.07	3.42±0.09
Chl *a/b*	3.90±0.04	4.03±0.02	4.07±0.03	3.88±0.01	3.90±0.04	4.03±0.05	3.81±0.07	3.75±0.02
Carotenoids (µg cm^{-2})	0.43±0.01	0.43±0.01	0.43±0.01	0.43±0.00	0.45±0.01	0.46±0.01	0.43±0.01	0.42±0.02

Supplementary Table 2. Granum diameter and repeat distance. Granum diameter was determined from transmission electron micrographs of wild-type (wt) and *vccn1-1* mutant leaves including those shown in Fig. 5. Data are means ±s.e.m. (n=350–450 grana from 60-90 plastids from leaves of two different plants per treatment). Values with different letters are significantly different across treatments according to Kruskal–Wallis non-parameteric ANOVA followed by the Mann–Whitney ranksum test (P<0.05). Granum periodicity expressed as repeat distance (RD) was determined by SANS in detached leaves and isolated thylakoids (Supplementary Fig. 20). Statistically averaged RD values for 25–30 leaves for each genotype are given together with error of the fitting. n.d., not determined because of the dense and uneven packing of leaves in the optical cell does not allow well-controlled homogenous illumination. For thylakoid membranes, RD was determined on freshly isolated membranes, which showed no variation during storage (~3 h); the error arises from the uncertainty of fitting.

Sample	Granum diameter (nm)	RD leaves (Å)	RD thylakoids (Å)
wt dark	406.5 ± 5.7^{a}	225±1	269±1
vccn1-1 dark	428.7 ± 5.8^{b}	220±1	263±1
wt light	380.4 ± 5.4^{c}	n.d.	n.d.
vccn1-1 light	431.7 ± 5.7^{b}	n.d.	n.d.

Supplementary Table 3. Primers used in this work.

Gene	Forward	Reverse	Experiment
AtVCCN1 (At3g61320)	CTGGTCCTGAATCCAACGAT	CAACCCATTTCTCGTGTGTG	RT-PCR
	GGAAGGAAAGCTTGGGTTGG	ATGACACTTAAGAGCAACAGGG	Quantitative PCR
	CTACATATGGGATTCCGCAAATCCGTCAA	TAGGAGCTCTCAAGATCTATGGCGACCATT	Cell-free *in vitro* expression
AtVCCN2 (At2g45870)	TGCCATGTGATTTATGGTTCA	TGTAGGAAAGTGGAATCGGAAT	RT-PCR
	CAAGGAAGGAAAGCTTGGGT	GCACTTAAGCGCCACAGG	Quantitative PCR
AtACTIN8 At1g49240	TGCAGACCGTATGAGCAAAG	CTGGAAAGTGCTGAGGGAAG	RT-PCR control
AtPEX4 (At5g25760)	CAGTCTGTGTGTAGAGCTATCATAGCAT	AGAAGATTCCCTGAGTCGCAGTT	Quantitative PCR control

References for Supplementary Information

1 Kane Dickson, V., Pedi, L. & Long, S. B. Structure and insights into the function of a Ca(2+)-activated Cl(-) channel. *Nature* **516**, 213-218 (2014).

2 Schranz, M. E. & Mitchell-Olds, T. Independent ancient polyploidy events in the sister families Brassicaceae and Cleomaceae. *Plant Cell* **18**, 1152-1165 (2006).
