# Supplementary material for: A voltage-dependent chloride channel fine-tunes photosynthesis in plants
Source: Nat Commun. 2016 May 24;7:11654. doi: 10.1038/ncomms11654 (PMC4890181; doi:10.1038/ncomms11654)
Supplement: Supplementary Data 1 — Expanded version of Supplementary Figure 2 and MAFTT alignment. [file ncomms11654-s2.docx]

**Supplementary Data 1: Expanded version of Supplementary Figure 2 and MAFTT alignment. a**, Expanded version of Supplementary Figure 2 with taxon names and sequence accession numbers. Bayesian posterior probabilities (≥0.95) are shown to the right of or above each node. Scale bar units are in substitutions/site. **b**, MAFFT alignment in FASTA format used to create Supplementary Figure 2, trimmed as described in the Methods.

**a**


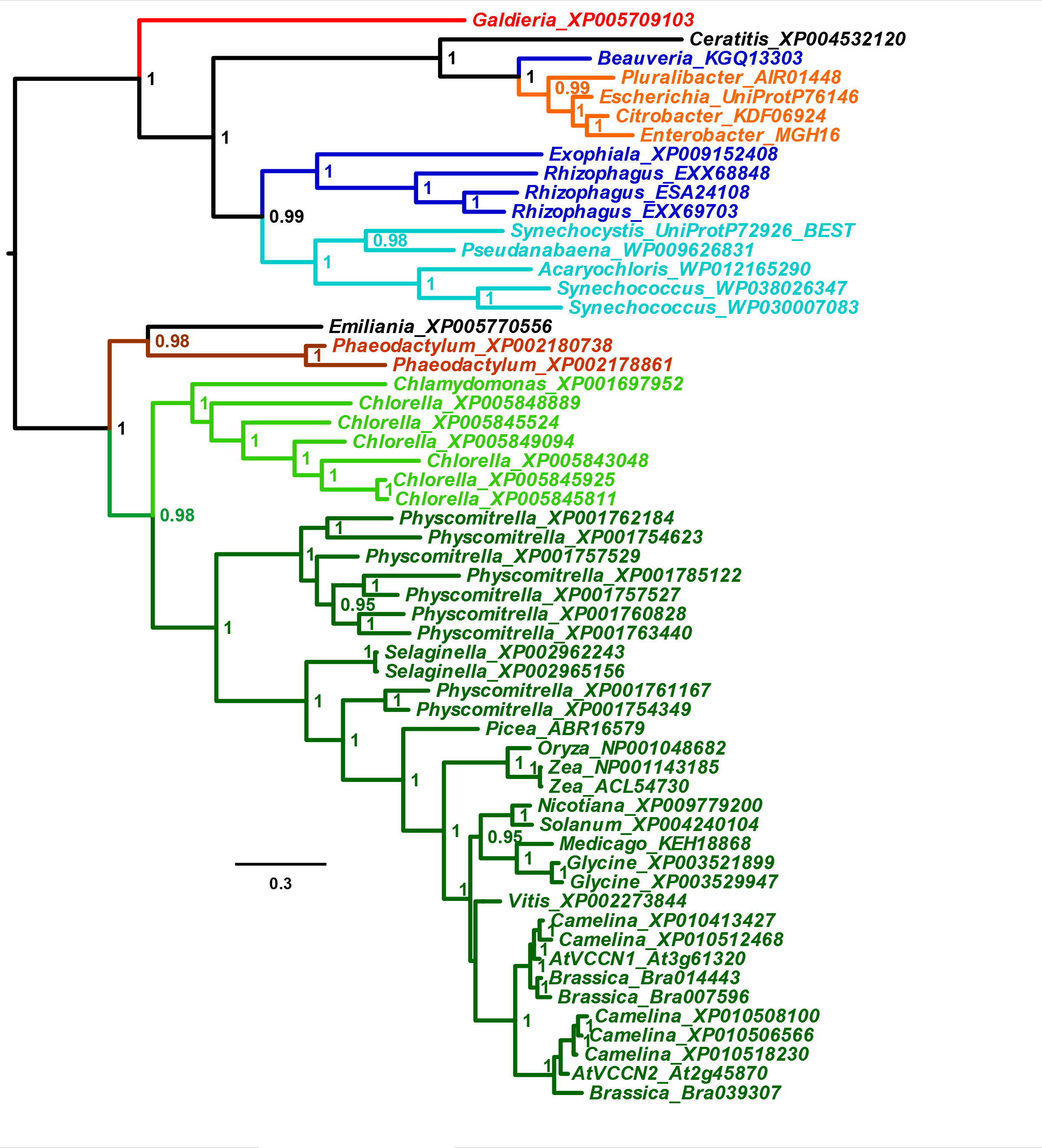


**b** 10 20 30 40 50 60 70

....|....|....|....|....|....|....|....|....|....|....|....|....|....|

**Galdieria_XP_005709103**  **RSCIGFARPGRYGYFKPASIVFGGFRLLRSHLKLNLCCSLKRAERYTSSDWSRCLL--------------**

**Ceratitis_XP_004532120**  **----------------------------------------------------------------------**

**Beauveria_KGQ13303**  **------------------------------------------MIVRPHQHWLARL---------------**

**Pluralibacter_AIR01448**  **------------------------------------------MIVRPPEHWLTRT---------------**

**Escherichia_UniProtP76146**  **------------------------------------------MIVRPQQHWLRRI---------------**

**Citrobacter_KDF06924**  **------------------------------------------MIVRPQQHWLRLI---------------**

**Enterobacter_MGH16**  **------------------------------------------MIVRPKQHWLQLI---------------**

**Exophiala_XP_009152408**  **--MDKSKDLNLAVTDFASSDGSKNSNLPSPGRNPKFTPIALRAPTRTSIDLDDYFVGPRDMNHHSKWPYF**

**Rhizophagus_ESA24108**  **-----------------------------------MSNRVDNPKVYNPYSYHRPHF--------------**

**Rhizophagus_EXX69703**  **-----------------------------------MVKR-NQPKVYDPYVYHKPHS--------------**

**Rhizophagus_EXX68848**  **---------------------------------------MEQEKVKKQLRYNPYV------------PEF**

**Synechocystis_UniProtP72926_BEST** **------------------------------------MSFFSHHPSRSPRIWTDIL---------------**

**Pseudanabaena_WP_009626831**  **--------------------------------------------MKKKDDWFRIA---------------**

**Acaryochloris_WP_012165290** **------------------------------------------MYTPTKSNWLATT---------------**

**Synechococcus_WP_030007083**  **---------------------------------------MITSDKLDKPDWLSTI---------------**

**Synechococcus_WP_038026347** **----------------------------------------MATNRFEKPDWLTTI---------------**

**Emiliania_XP_005770556**  **--------------------------------EFKEAGREFRQDVYSYNDWRWHRESGHIASAIS-----**

**Phaeodactylum_XP_002178861** **SSSYRTQAFIVPSGTSSRVSVAVRSLPPPRDIAYGEESRKYRRTVYTHDDWVNHRSPDRFWRNII-----**

**Phaeodactylum_XP_002180738** **VQHNGVRTIATPSTPLYGNTKQPPALPPIKDISYGEESRKYRRTVYSHDDWVKHRSSDRFLRNLL-----**

**Chlamydomonas_XP_001697952** **--------------------------------------------VYDFPQWQKHRSSYRFAERLF-----**

**Chlorella_XP_005843048** **PRTRSLNRGRLTQLRVAAAASSPPLPAKLSGDDLKEAELKKLRTVADFEFWKRHRSSSRYWRHVL-----**

**Chlorella_XP_005849094** **QQFPGSELDAAAKAEAAAAKLQR---YPWHSDEWKEEYRKQVRVTFDFERWRTHRSSSRFWRHMA-----**

**Chlorella_XP_005845811** **CRCNRCGRGVALRTSAVANTDKREQPHPYFTDEWKEYNRQFQRPVFDFERWKTHRSSSRYLRHVL-----**

**Chlorella_XP_005845925**  **CATSRCSR-AALRTLAVASTDKTEPEHPYFSDDWKEYNRRFQRPVFDFERWKTHRSSSRYLRHVL-----**

**Chlorella_XP_005845524** **QVAEQAVVDAVAQTAGDRATSSSAAAGSREVDPIKEGSRKYRRTVYDFENWRQHRSTKRYMRHAK-----**

**Chlorella_XP_005848889**  **--------------------------------------------VFTFERWNKHRNSMRYAKHLA-----**

**Physcomitrella_XP_001754623** **-----------------------------------------RNTVYGHDEWRRHKSSWRHARHVI-----**

**Physcomitrella_XP_001762184** **----------------------------------EEAGIMHRNSVYGHGEWKKHKSSWRHVRHVC-----**

**Physcomitrella_XP_001757529** **---------------------------------------MHRNTVYGHTEWRRHKSCWRHSRHLS-----**

**Physcomitrella_XP_001757527** **-----------------------------------------RNAVYGHDEWAKHKSCWRHGRHLK-----**

**Physcomitrella_XP_001760828** **---------------------------------------MHRNKVYGHAEWAKHKSSWRHGRHLM-----**

**Physcomitrella_XP_001763440** **------------------------------------QKIMHRNKVYGHLEWARHKSSWRHGRHIF-----**

**Physcomitrella_XP_001785122** **--------------------------------------------------MGLHKSSWRHGRHIQ-----**

**Selaginella_XP_002962243**  **-----------------------IASVPEWADAQKEQNMPNRRAFYSNNDWLRHRSSLRHARHMA-----**

**Selaginella_XP_002965156**  **-----------------------IASVPEWADAQKEQNMPNRRAFYSNNDWLRHRSSLRHARHMA-----**

**Physcomitrella_XP_001761167** **EKNKNVNGKLSAVTNFDPRRPFDLTWIGAWADEVSERGMHKRRTFYSHDDWLQHRSSTRHYRHFT-----**

**Physcomitrella_XP_001754349** **--------------------------------------MLKRRTFYSHDDWLRHRSSTRHYRHFA-----**

**Picea_ABR16579**  **FTIQCKQHEQHENETKISSVIERLNSIPEWVDGIKEGGMKKPKSLYTHIEWVRHRSSTRHIRHVV-----**

**Oryza_NP_001048682**  **FRDPPKAPDTN---SNTNPLLSLLSAVPDWADAVSERRIRDRRPLYTHADWREHRSSRRHLRHLL-----**

**Zea_ACL54730**  **FPDPPPPPASMPPIPSPNPVLSLLSAVPDWADAIQERRVRDRRPLYDHATWRHHRSSRRHLRHLL-----**

**Zea_NP_001143185**  **FPDPPPPPASMPPIPSPNPVLSLLSAVPDWADAIQERRVRDRRPLYDHATWRHHRSSRRHLRHLL-----**

**Nicotiana_XP_009779200** **CC--PQQTPE--NQNSTSALISILRIIPDWADRIQEEGMKKKRSLYKHESWVQHRSSLRHVRHLF-----**

**Solanum_XP_004240104** **CSSQPSRTPQ--NSNLISTLVKILRVVPDWADKIQEGGMRKKRSLYKHETWVQHRSSLRHVRHLF-----**

**Glycine_XP_003521899**  **SASLPPGPTS----GPAQTLISLLRSIPDWADAVQERGMQKKRALYTHQNWRDHRSSLRHLRHVF-----**

**Glycine_XP_003529947**  **LASFPPGPTS----GPAQTLISLLRSIPDWADAVQERGMQKKRALYTHQNWRDHRSSLRHLRHVF-----**

**Medicago_KEH18868**  **--------------------------------------MQKNRTLYNHTNWVTHRSSLRHVRHFF-----**

**Vitis_XP_002273844**  **SADSPPPPSS----TQNLTLISILRTVPDWADAIKERGMQQKRSLYNHETWVEHRSSRRHVRHLL-----**

**AtVCCN1_At3g61320**  **VSSGPESNDS-GHETLTDKLIHLLRAVPDWADEIKERGMQQKRSLYTHEKWVEHRSSLRHVRHLL-----**

**Brassica_Bra007596**  **VSPGPESDLS-GAKTLTENLISLLRSVPDWADEIKERGMQQKRTLYTHEKWVEHRSSLRHVHHLF-----**

**Brassica_Bra014443**  **VSSGPEP----GAKTLTENLISLLRAVPDWADEIKERGMQQKRTLYTHEKWVEHRSSLRHVRHLV-----**

**Camelina_XP_010512468**  **VSSDPNSNDSGGDKALSEHLISFLRAVPDWADEIKERGMQQKRSLYTHEKWVEHRSSLRHVRHLL-----**

**Camelina_XP_010413427**  **VSSGPDSNDS-GEKNLTEQLISLLRAVPDWADEIKERGMQQKRSLYTHEKWVEHRSSLRHVRHLL-----**

**AtVCCN2_At2g45870**  **VSSGPKSDDS----PLSEKLISLLKAVPNWSDGIKERRMQQKRSLYTHENWVRHRSSLRHLRHVS-----**

**Brassica_Bra039307**  **VSSDPNSSEN--SINLSSKLISLLKAVPNWSDGIKERRMRQKRSLYTHENWVRHRSSLRHLRHVS-----**

**Camelina_XP_010508100**  **ASSAPKSNDS----SLSDKLLSLLKAVPNWSDGIKERRMQQKRTLYSHENWVRHRSSLRHLRHVS-----**

**Camelina_XP_010518230**  **ASSAPKSDDS----SLSEKLISLLKAVPNWSDGIKERRMQQKRTLYSHENWVRHRSSLRHLRHVS-----**

**Camelina_XP_010506566**  **ASSAPKSDDS----SLSDKLISLLKAVPNWSDGIKERRMQQKRTLYSHENWVRHRSSLRHLRHVS-----**

80 90 100 110 120 130 140

....|....|....|....|....|....|....|....|....|....|....|....|....|....|

**Galdieria_XP_005709103**  **SLPKSIILSRIRSHLFWTCCVSFLVSLFDH--------------------------FFKVPPFNPIPHTL**

**Ceratitis_XP_004532120** **-------MRKISLQMLFFVGLNIAYLFILP--------------ILHLHGIV----------AKESPVYF**

**Beauveria_KGQ13303**  **FVWHGSVLEKIYSRLLLNLLLSISVIFFLP--------------WYETLGIK----------LTIAPFSI**

**Pluralibacter_AIR01448** **LVWHGSVLSKIFTRLLLNFLLSVAVIIMLP--------------WYTSLGIR----------LTVAPFSI**

**Escherichia_UniProtP76146** **FVWHGSVLSKISSRLLLNFLFSIAVIFMLP--------------WYTHLGIK----------FTLAPFSI**

**Citrobacter_KDF06924** **FVWHGSVLAKISSRLLLNFLLSIVVIVLLP--------------WYTMLGIK----------FTLAPFSI**

**Enterobacter_MGH16**  **FVWHGSVLPKIYTRLLLNFLLSIAVILMLP--------------WYTSLGIK----------FTVAPFSI**

**Exophiala_XP_009152408** **LRLHGSVLPKMILPLTFVAAWATAITCISK--------------FVYVLG------------INSVLLTI**

**Rhizophagus_ESA24108** **IRWRGSVIPKVLPSTIVVTLLAVIVCIVNL---------------ETKVKIG----------IPSTFIPV**

**Rhizophagus_EXX69703** **IRWKGSVLPRVLPSTLVVTIVAVIVTVLYE---------------KTDVKLG----------IDSTFIPI**

**Rhizophagus_EXX68848** **LRLKGTVLPHVIVQTIIVTLISTVVVILFE--------------LT-DIKLSVSKAPPT--SISNIFTQI**

**Synechocystis_UniProtP72926_BEST** **LRWQGSVIPAIASRVLVCMAFSLGVTLVDG--------------WGYKFS------------IPIQESIV**

**Pseudanabaena_WP_009626831** **LRWRGSVVPEVLPRSLLCGLFGVFIYILHI--------------SNIRVS------------LPILGSII**

**Acaryochloris_WP_012165290** **FRLDGSVAGIILPRILAFTGFTLAICSLDY--------------FDYPIYLQEI--------GDLTTNVV**

**Synechococcus_WP_030007083**  **FQVKGAVLSMILPRILFFCILTAALTFIYL--------------QGLPIYLEKL--------GDLTTNVI**

**Synechococcus_WP_038026347** **FQLKGSVILIILPRILFFCGFTTAITLLYT--------------LDFPIYFQKL--------GDLTTNVI**

**Emiliania_XP_005770556**  **SVFTSGVGKAMWRETFFVIATAAAVYLYNIGVPVLAAKTAASLPIVAALLGR--LPLLH---LSLLPLTL**

**Phaeodactylum_XP_002178861** **AMPTSGVYKNLAKECIATTAVATAIVVYNA---LVGGYTDFGGVQHAAVLQNELLPKIG---MPVSPFTV**

**Phaeodactylum_XP_002180738** **AIGSSGVYKSLAKEVLATTGVATFIVLYNC---LVGGYTDLEGIKHSALIESVWAPLMA---LPLAPFTL**

**Chlamydomonas_XP_001697952** **QLSQSHILQNALPAISWVTLVATLVASYGY--------------SYDQHMLPDVFPSISPNASCTAFISN**

**Chlorella_XP_005843048**  **GIFDSRTFSWVAAPLSYVMLLTTGVCLYYT--------------LAEAGIVPEVIPEIS--ASAAAPFGL**

**Chlorella_XP_005849094**  **GLAQSNTAQGLAQPLAYVMAVSLGVAAYHV--------------AAAAGWLPLW-PVLK--LAANAPFGL**

**Chlorella_XP_005845811**  **GMGDSKIVQGLAKPLAYVMTLATGVALYHT--------------LAEAGYLTDV-PDLK---ATNAPFGL**

**Chlorella_XP_005845925**  **GIFDSKIVQGLAKPLAYVMTLATGVALYHT--------------LAEAGYLTDV-PDLK---ATNAPFGL**

**Chlorella_XP_005845524**  **GLLGSRIFRGLASPLLYILAVSASVAVWNT--------------LVETGLAPDVLPELH--MSNNGPFGL**

**Chlorella_XP_005848889**  **HMFTSRVFRQLLGPVLAVMTIALAVGVYET--------------LVGAGALPGHWPHVT--LALGQGFNL**

**Physcomitrella_XP_001754623** **SIAASGVIAALGPPVLLSTITAVFVTVINH--------------GVQHMLVASWVPYLK---VSPIPFTF**

**Physcomitrella_XP_001762184** **TILSSGLIRAIGPPVFLCTLVSVFVAVINH--------------CVDNGVLPSWFPVLK---VATLPFTL**

**Physcomitrella_XP_001757529** **SIFSSGVIFTLGPPVILCTLVATLVSVINH--------------LVQDRRLPKWMPLLH---VASLPFTL**

**Physcomitrella_XP_001757527** **TIFASRPIVATGPPVAFCTLIAVFVVIFNH--------------SVLVGHFPVWVPVIQ---VASIPFAL**

**Physcomitrella_XP_001760828** **SILSSGVISAVGPPVLACTLLATFVTVFNY--------------FVKVGRLPNWIPILE---VSSLPFTL**

**Physcomitrella_XP_001763440** **SILSSGVIFAVFPPVLVCTLFGTFVTIFNH--------------FVQNGHLPTWMPILH---VASLPFTL**

**Physcomitrella_XP_001785122** **TAFSTGVISSIIPRVFFCTLISVLVTIFNH--------------AVMEGVLPHWVPSLR---VPTLPMSL**

**Selaginella_XP_002962243**  **STFSSRVIISLIPPVFTVTGISVLVTLYNA--------------LVESGWAPGFLPVLH---APSLPYEL**

**Selaginella_XP_002965156**  **STFSSRVIISLIPPVFTVTGISVLVTLYNA--------------LVESRWAPGFLPVLH---APSLPYEL**

**Physcomitrella_XP_001761167** **STLSSRVIVSLIPPVGTMTAISVAISIYNS--------------IVLSGCLPSFIPLLH---ASPLSYQL**

**Physcomitrella_XP_001754349** **SSFSSRAILSLIPPVGTMTAISVFVALYNT--------------VVLSGWLPSFFPIFH---ASSLSYQL**

**Picea_ABR16579**  **SSLSSRVTISLIPPVFIFTAIAVAIAAYNT--------------AVVCEWFPPFMPLLH---ASSLPYQL**

**Oryza_NP_001048682**  **SSLTSRVILSLAPPVSAFTAFAAAIATYNT-------------------LLPAY--ALT---ASSLPYQL**

**Zea_ACL54730**  **TSLSSRAILSLAPPVSAFTAFAAAIATYNT-------------------LLPAY--ALT---ASSLPYEL**

**Zea_NP_001143185**  **TSLSSRAILSLAPPVSAFTAFAAAIATYNT-------------------LLPAY--ALT---ASSLPYEL**

**Nicotiana_XP_009779200**  **SSLNSRVILSLVPPVIAFTSVAVVIASYNS--------------AVSMHWLPEFFPVLR---ASPLPYQL**

**Solanum_XP_004240104**  **SSFNSRVVLSLIPPVIAFTSFAFVIASYNS--------------AVSFHWLPEFFPILR---ASPQPYQL**

**Glycine_XP_003521899**  **SSLSSRVILSLVPPVLFFTAFSAAIAAYNE--------------ALLLHLLPEFLPLLR---TSSLPYQL**

**Glycine_XP_003529947**  **SSLSSRVILSLVPPVLFFTAFAATIAAYNE--------------ALLLHLLPDFLPLLR---ASSLPYQL**

**Medicago_KEH18868**  **SSFSSRVILSLVPPVLFFTSFAAVIAAYNS--------------AVWFHYLPEFFPVLR---ASSLPYQL**

**Vitis_XP_002273844**  **SSFSSRVILSLIPPVIAFTSVAVIVASYNS--------------AVTFHWLPEFFPLLR---ASSLPYQL**

**AtVCCN1_At3g61320**  **SSFSSRVILSLIPPVFFFTSVAVVIASYNS--------------AVALDWLPGIFPILR---SSSLPYQL**

**Brassica_Bra007596**  **SSFSSRVILSLIPPVFFFTSVAIFIASYNS--------------AVALDWLPSVFPILR---SSSLPYQL**

**Brassica_Bra014443**  **SSFSSRVILSLIPPVFFFTSVAVVIASYNS--------------AVALEWLPGIFPILR---SSSLPYQL**

**Camelina_XP_010512468**  **SSFSSRVILSLIPPVFFFTTVAVVIASYNT--------------AVALDLLPGIFPILR---SSSLPYQL**

**Camelina_XP_010413427**  **SSFSSRVILSLIPPVFFFTSVAVVIASYNT--------------AVALDWLPGIFPILR---SSSLPYQL**

**AtVCCN2_At2g45870**  **SSPSSRVILSLIPPVFFFTTVAILIAGYNS--------------AVDLDWLPDFFPVLR---ASPLPYQL**

**Brassica_Bra039307**  **SSASSRVILSLIPPVFFFTTVAVLIAGYNS--------------AVGSELLPSFFPVLR---ASPLPYQL**

**Camelina_XP_010508100**  **SSTSSRVILSLIPPVCFFTTVAVLIASYNS--------------AVGLNLLPDYFPVLR---ASPLPYQL**

**Camelina_XP_010518230**  **SSTSSRVILSLIPPVCFFTTVAILIASYNS--------------AVGLGWLPDFFPVLR---ASPLPYQL**

**Camelina_XP_010506566**  **SSTSSRVILSLIPPVCFFTTVAILIASYNS--------------AVGLGWLPDYFPVLR---ASPLPYQL**

150 160 170 180 190 200 210

....|....|....|....|....|....|....|....|....|....|....|....|....|....|

**Galdieria_XP_005709103**  **LGSAMGLLLVFRTNAAYDRFWEARKLVGVLAVQSREMTRCIH------------SYFNEEKF--------**

**Ceratitis_XP_004532120**  **IGVSISIFLGFRNNVAWGRYSEARKLWGELLIVTRSLMREFI------------NTSLAS----------**

**Beauveria_KGQ13303**  **LGVAIAIFLGFRNSACYARFNEARLLWGQLAITSRSLLREVK------------TLAGDQ----------**

**Pluralibacter_AIR01448**  **LGVAIAIFLGFRNNACFARYVEARHLWGELVIVSRTALREVK------------NTLPE-----------**

**Escherichia_UniProtP76146**  **LGVAIAIFLGFRNNAGYARYVEARKLWGQLMIASRSLLREVK------------TTLPD-----------**

**Citrobacter_KDF06924**  **LGVAIAIFLGFRNNACYSRYVEARQLWGQLMIASRSLLREVK------------TVLPD-----------**

**Enterobacter_MGH16**  **LGVAIAIFLGFRNNACYSRYVEARLLWGQLMIAARSLFLEVK------------NTLPD-----------**

**Exophiala_XP_009152408**  **TGFVVGLALSFRSTTAYERYSEGRRYWAQLVVTSRNLARLIW------------VHTSERHDISEEQGKA**

**Rhizophagus_ESA24108**  **LGFVVGLLLTYRTNTAYDRYWEGRRLWAVMVVAIRNLTRNIW------------INIKEDDGKSD-----**

**Rhizophagus_EXX69703**  **LGFVVGLLLTYRTNTAYDRYWEGRRFWSVLVVAIRNLTRTIW------------INVKEDEGTKD-----**

**Rhizophagus_EXX68848**  **IGIVVGLLLTYRTNTAYDRYWEGRKLWSTMVVQIRNFTRYMW------------IGVREDGKKKGADPHG**

**Synechocystis_UniProtP72926_BEST** **PSIVLGLLLVFRTNTAYERFWEGRKAWGTMVNTIRNLSRIIW------------VSVAEPSPQA------**

**Pseudanabaena_WP_009626831** **PNIVLGLLLVFRTNTAYERFWEGRKAWGLLVNTVRNLSRQIL------------VAILEKEPRD------**

**Acaryochloris_WP_012165290** **YNLVLGLLLVFRTNTAYDRFWDGRKAWGTLVVNSRNFARQVA------------LLWPSPKATP------**

**Synechococcus_WP_030007083**  **YNLILGLLIVFRTNTSYERFWEGRKAWGGIVVNIRNLAQEIL------------IGITTSTEKE------**

**Synechococcus_WP_038026347** **YNLILGLLVVFRTNTSYDRFWEGRKAWGVLAIDIRNLAQEIR------------VGVAERDETD------**

**Emiliania_XP_005770556**  **SSPALFLLLVFRTNNSYDRWWEARKVWGGVINASRDLARQAL------------ALVRD-----------**

**Phaeodactylum_XP_002178861** **SGSFLGFLLIFRTNSSYKRWDEARKNWGMNINHTRDLVRMGT-------------AFYDKTGVTDEQR--**

**Phaeodactylum_XP_002180738** **SSPSLGLLLVFRTNTSYQRWDEARKNWGMNINHTRDLVRMGT-------------SFYDNAAVSSEQR--**

**Chlamydomonas_XP_001697952** **TSVALSLLLVFRTNSSYGRWDEARKMWGGLLNRSRDIMRQGA------------TCFPDDQ---------**

**Chlorella_XP_005843048**  **TSFALSTLLVLRTNTSYQRWDEARKMWGLIVNRTRDISRQAV------------GYIPPH-------Q--**

**Chlorella_XP_005849094**  **TSFALSLLLVFRTNSSYGRWDEARKMWGLVVNRSRDLTRQAL------------GYIPAH-------Q--**

**Chlorella_XP_005845811**  **TSFALSLLLVFRTNTSYQRWDEARKMWGSMVNRSRDFTRQAL------------GYVPYS-------Q--**

**Chlorella_XP_005845925**  **TSFALSLLLVFRTNTSYQRWDEARKMWGSMVNRSRDFTRQAL------------GYVPES-------Q--**

**Chlorella_XP_005845524**  **TSFALSLLLV--TNASYARWLDARKAWGMLVNRSRDITRQAL------------TCFPAA-------D--**

**Chlorella_XP_005848889**  **TAFALSLLLVFRTNSSYDRWWEARKLWGGVVNRCRDIVRQVG--------PQGLVFFRDED---------**

**Physcomitrella_XP_001754623** **ISPVLAFLLVFRTNSSYQRFDEARKVWGSNVNRCRDLARQAL------------SWIKNPE------D--**

**Physcomitrella_XP_001762184** **TSPVLALLLVFRTNTSYQRFDEARKAWGSNVNRARDLARQAL------------TWIRNPG------D--**

**Physcomitrella_XP_001757529** **TAPVLALLLVFRTNASYSRFDEARKAWGSNVNRTRDLARQAL------------TWIRMPC------D--**

**Physcomitrella_XP_001757527** **TSSVLSLLLVFRTNSSYNRFDEARKAWGSNVNRTRDLARQAL------------TWIRSPA------D--**

**Physcomitrella_XP_001760828** **TSSVLSLLLVFRTNSSYNRFDEARKIWGSNVNRTRDLARQAL------------SWIRSPA------D--**

**Physcomitrella_XP_001763440** **TSSVLSLLLVFRTNSSYNRFEEARKFWGSNVNRTRDLVRQSL------------TWISQPG------D--**

**Physcomitrella_XP_001785122** **TAPVLSLLLVFRTNSSYNRLDEARKAWGSNVNRTRDVSRQAL------------SWICDPD------D--**

**Selaginella_XP_002962243**  **TAPALALLLVFRTDTSYSRYDEARKTWTEVISSTKNLARLTE------------AWIHNDD---------**

**Selaginella_XP_002965156**  **TAPALALLLVFRTDTSYSRYDEARKTWTEVISSTKNLARLTE------------AWIHNDD---------**

**Physcomitrella_XP_001761167** **TAPALALLLVFRTEASYSRYDEARKTWTKVISSSKDMVRQSM------------TWAQRPD------D--**

**Physcomitrella_XP_001754349** **TAPALALLLVFRTEASYSRYDEARKTWTEVISSSKDMARQAL------------AWSQHPA------D--**

**Picea_ABR16579**  **TAPALALLLVFRTEASYSRYDEGRKAWTKVISDAKDFARQSI------------TWIRGAD------N--**

**Oryza_NP_001048682**  **TAPALALLLVFRTEASYARFDEGRKAWMRVIAAAADLAGMAMRHH----------------------N--**

**Zea_ACL54730**  **TAPALALLLVFRTEASYARFDEGRKAWMRVLASAADLAGMLMRHPFPNNLHTGGAPNPGPGGRQA-DD--**

**Zea_NP_001143185**  **TAPALALLLVFRTEASYARFDEGRKAWMRVLASAADLAGMLMRHPPPNNLHTGGAPNPGPGGRQA-DD--**

**Nicotiana_XP_009779200**  **TAPALALLLVFRTEASYSRFETGKKAWTKVIAGTNDFARQVI------------ACVDKSD---------**

**Solanum_XP_004240104**  **TAPALALLLVFRTEASYSRFEAGKKAWTKVIAGTNDFARQVI------------ACVDKRD---------**

**Glycine_XP_003521899**  **TAPALALLLVFRTEASYSRFVEGKKAWTNVIAGTHDFARQVA------------AIVDDGGGGGN--N--**

**Glycine_XP_003529947**  **TAPALALLLVFRTEASYSRFVEGKKAWTIVIAGTHDFARQVA------------AVVVDDDGG----N--**

**Medicago_KEH18868**  **TAPALALLLVFRTEASYSRFVEGKKAWTAVIAAASDFARLVM------------ATVDVSGKG----D--**

**Vitis_XP_002273844**  **TAPALALLLVFRTEASYSRFEEGRKAWTKIIAGTNDFARQVV------------AGVESSG------D--**

**AtVCCN1_At3g61320**  **TAPALALLLVFRTEASYSRYEEGRKAWVGIIAGTNDLARQVI------------CSVDSSGD-----E--**

**Brassica_Bra007596**  **TAPALALLLVFRTEASYSRYEEGRKAWVGIIAGTDDLARQVI------------CSVDGSGD-----E--**

**Brassica_Bra014443**  **TAPALALLLVFRTEASYSRYEEGRKAWVRIIAGTDDLARQVI------------CSVDGSGD-----E--**

**Camelina_XP_010512468**  **TAPALALLLVFRTEASYSRYEEGRKAWVGIIAGTNDLARQVI------------CSVDSSGD-----E--**

**Camelina_XP_010413427**  **TAPALALLLVFRTEASYSRYEEGRKAWVGIIAGTNDLARQVI------------CSVDSSGD-----E--**

**AtVCCN2_At2g45870**  **TAPALALLLVFRTEASYSRFEQGRKAWVKIISGTNDLARLVI------------SSV-HGSG-D---E--**

**Brassica_Bra039307**  **TAPALALLLVFRTEASYSRFEQGRKAWVKIITGTNDLARQVV------------SSVNGPSGDD---E--**

**Camelina_XP_010508100**  **TAPALALLLVFRTEASYSRFEQGRKAWAKIINGTNDLARLVV------------SSVRHGSA-D---E--**

**Camelina_XP_010518230**  **TAPALALLLVFRTEASYSRFEQGRKAWAKIINGTNDLARLVI------------SSVRDGSG-D---E--**

**Camelina_XP_010506566**  **TAPALALLLVFRTEASYSRFEQGRKAWAKIINGTNDLARLVI------------SSVRHGSA-D---E--**

220 230 240 250 260 270 280

....|....|....|....|....|....|....|....|....|....|....|....|....|....|

**Galdieria_XP_005709103**  **------QVIKIRLVMLLKLFLVAFLQHV-QGTADVVS-FRKMIRS-NPEYSDSSSHFVDIFADEIVHSP-**

**Ceratitis_XP_004532120**  **------YEEKKAVVDLLIAYATVLKDTL-RHEP-----AAAAMC-------EDR-------LVTGAGVM-**

**Beauveria_KGQ13303**  **------PEAVQRFVNLQVAFCNSLRMTL-RKKP-----QQDTLSR-YLS--PQD-------LAWVSESH-**

**Pluralibacter_AIR01448**  **------GADAAAFARLQIAFVHSLRMQL-RGLP-----QRETLAR-YLD--EAQ-------LEEVLAVQ-**

**Escherichia_UniProtP76146**  **------SASVREFARLQIAFAHCLRMTL-RKQP-----QAEVLAH-YLK--TED-------LQRVLASN-**

**Citrobacter_KDF06924**  **------DRELGQFVRLQIAFAHCLRMTL-RRKP-----QAEPLAK-YLG--TAD-------LQRVFASH-**

**Enterobacter_MGH16**  **------DKHLGEFVRLQIAFANCLRMTL-RREL-----NAEQLSR-YLA--AED-------LRKVMDAN-**

**Exophiala_XP_009152408**  **D-----LLAKLTALNLINAFAVALKHRL-RFEP---AVDYPDLQP-LIGH-----------LQTLASQA-**

**Rhizophagus_ESA24108**  **------LLEKKTAINLLIGFAVATKHYL-REED---GLLHDDLKP-LISNIKSN-------LPGFKPFN-**

**Rhizophagus_EXX69703**  **------ILEKKTAINLLLGFAVATKHYL-REEE---GSNHEDLKY-LISNIKSS-------LPGFAPIE-**

**Rhizophagus_EXX68848**  **QAKPEIVIEKRTALNLLLGFTVAVKHYL-REEP---GYHYKDLEN-LISNIRSS-------LPEFSSANN**

**Synechocystis_UniProtP72926_BEST** **------HQDKIKILHLLVAFAVATKLHL-RSQP-----LNEEIWA-LLP--ESG-------YRKLEDLN-**

**Pseudanabaena_WP_009626831** **------RQAKIAAVKMLPAFAIALKLHL-RSES-----INSELAA-NLS--PEQ-------FERLKTMN-**

**Acaryochloris_WP_012165290** **------STDRDTLLNLLVAIALATKLHL-RSEP-----IGDTLQD-LIT--PEQ-------AQILADAQ-**

**Synechococcus_WP_030007083**  **------IQGKKQALNLLLAFAIATKLHL-RGDQ-----VNDRLEA-LVE--PEQ-------VAQLKDSK-**

**Synechococcus_WP_038026347** **------FLEKQSVMRLLSAFAIATKLHL-RGEA-----VNDELKA-LLT--PAR-------AEQLEASS-**

**Emiliania_XP_005770556**  **------AELKKLMVSQIASYARVLKYHLGPPTPEARDLLRNELVDNRLP--ADQ-------VRVIMEAK-**

**Phaeodactylum_XP_002178861** **------KKDLQALSLATWSFVRAMKRHL-SPEQEDEQDFRRELHE-RLP--PRQ-------AQAIIDAA-**

**Phaeodactylum_XP_002180738** **------AKDLKALSLATWSFVRAMKRHL-SPESEDEQDFRRELFE-RLP--APQ-------AQAIIDAA-**

**Chlamydomonas_XP_001697952** **------VEAKKALARWTVAFSRALRIHF-QPEV---T-IESELQN-ILT--PAE-------LQMLAKSQ-**

**Chlorella_XP_005843048**  **------AELQDMFCRWLVAYCRSLMCHL-RAGE---D-LEAELKG-KLT--DIE-------LKALLAST-**

**Chlorella_XP_005849094**  **------AELQSMLCRWVVAYSRCLMCHL-REGE---D-LEAELRG-VLL--PEE-------VAELLAAE-**

**Chlorella_XP_005845811**  **------PELRSMLVRWSIAYPRALMCHL-RPGE---N-IEEEVKD-ILK--PEE-------VKALAAST-**

**Chlorella_XP_005845925**  **------PELRSMLVRWSIAYPRALMCHL-RPGE---N-IEEEVKD-ILK--PEE-------VKALAAST-**

**Chlorella_XP_005845524**  **------RPLLDMLCRWTAAYSRALMCHV-REDS---D-LEAELRK-VLP--AHE-------VEAVVLAK-**

**Chlorella_XP_005848889**  **------AHLKELLARWTMAFPRVLMCHL-REDM---D-VGKEVAH-ILT--AHE-------VAVMCAAA-**

**Physcomitrella_XP_001754623** **------AARLECLLRFLKAYPYYLKLHL-TQEGPSSS-TTSEIKD-ILK--DEE-------FHKVSLVQ-**

**Physcomitrella_XP_001762184** **------SKKLQCLLRYTKAYSFCLMHHL-REEG---C-LRKELEATIVN--EEE-------VECVMNSK-**

**Physcomitrella_XP_001757529** **------APKLHCLLRHIKAYSLCLKDHM-TEDN---T-LREELTA-VLE--PSE-------VDCAMSSQ-**

**Physcomitrella_XP_001757527** **------LPKLHCLLRHIKAYSYCLKDHL-TQDN---T-LREELAK-VLE--PTE-------LELVLSSK-**

**Physcomitrella_XP_001760828** **------AYKLSCLLRHIKAYPFSLKDHL-TEDF---I-LKDELDQ-ILE--PQE-------LEALMATK-**

**Physcomitrella_XP_001763440** **------SLILLSLLRHIKAYSFCLKDHL-TEDE---T-LRDDLVG-IVE--PHE-------LESILSSP-**

**Physcomitrella_XP_001785122** **------ADKLQSLLRHIKAFSYCLKDHL-TQEN---L-LQEELAR-VLE--PRE-------VELVLKSS-**

**Selaginella_XP_002962243**  **------------LMRYIVAFPLALKCHL-IMGS---D-MEADLRK-VLD--ERD-------LAFVLNAK-**

**Selaginella_XP_002965156**  **------------LMRYIVAFPLALKCHL-IMGS---D-MEAELRK-VLD--ERD-------LAFVLNAK-**

**Physcomitrella_XP_001761167** **------NRRKKLLLDYILAFSVALKCHL-LYNS---D-IEEELSE-ILE--KDD-------LALVLSAE-**

**Physcomitrella_XP_001754349** **------HRKKKLLLDYILAFPVALKCHL-LYDS---D-IAEELRE-ILE--EDD-------LALVLKAE-**

**Picea_ABR16579**  **------VHLKARLLQYIMAFPVVLKCHI-THGS---D-MRHDLGT-LLH--EDD-------LETVLSSQ-**

**Oryza_NP_001048682**  **------PPATRALLNYILAFPLALKCHI-ICHS---D-IKRDLQG-LLS--EDD-------LNVVLRSK-**

**Zea_ACL54730**  **------EPLRRALVNYVLAFPVALKCHI-ICDS---D-VKGDLEG-LLG--EDD-------LSVVLASK-**

**Zea_NP_001143185**  **------EPLRRALVNYVLAFPVALKCHI-ICDS---D-VKGDLEG-LLG--EDD-------LSVVLASK-**

**Nicotiana_XP_009779200**  **------AVLKEALLQYIMAFPVALKCHI-TYGS---D-IASDLKN-LLE--ADD-------LALVLSSK-**

**Solanum_XP_004240104**  **------DVLKEALLQYIMAFPVALKCHI-VYDS---D-IASDLKN-LLE--ADD-------LAVVLSSK-**

**Glycine_XP_003521899**  **------FAIKHALLHYIIAFPIALKCHV-LYGS---D-VRRDLQH-LLE--VDD-------LVVVMNSE-**

**Glycine_XP_003529947**  **------FAIKHALLHYIIAFPIALKCHV-LYGS---D-VRSDLQH-LLE--VDD-------LAVVMNSK-**

**Medicago_KEH18868**  **------FQVKKELLNYIIAFPIVLKCHV-LYGS---D-VERDLQH-LLE--VDD-------IALIMKSN-**

**Vitis_XP_002273844**  **------ALLKKALLQYIMAFPVALKCHV-IYGS---D-IRQDLQN-LLE--VDD-------LAVVLSSK-**

**AtVCCN1_At3g61320**  **------LIIKDLLLRYIAAFPVALKCHV-IYGS---D-IARDLRN-LIE--ADD-------LSLILQAK-**

**Brassica_Bra007596**  **------LVIKDLLLRYVAAFPVALKCHV-TYGS---D-VARDLRN-LIE--ADD-------LSLIIESK-**

**Brassica_Bra014443**  **------LVIKDLLLRYIAAFPVALKCHV-IYGS---D-IARDLRN-LIE--ADD-------LSLILESK-**

**Camelina_XP_010512468**  **------LVIKDLLLRYVAAFPVALKCHV-IYGS---D-VGRDLRN-LIE--ADD-------LSLILQSK-**

**Camelina_XP_010413427**  **------LIIKDLLLRYVAAFPVALKCHV-IYGS---D-IARDLRN-LIE--ADD-------LSLILQSK-**

**AtVCCN2_At2g45870**  **------LIIRDALLRYIVAFPVALKCHV-IYGS---D-IASDLKN-VIE--VDD-------LSLILQSK-**

**Brassica_Bra039307**  **------VIIRDALLRYIAAFPVALKCHV-IYGS---D-IADDLRN-VVE--EDD-------LSLILKSK-**

**Camelina_XP_010508100**  **------LIIKDSLLRYIAAFPVALKCHV-IYGS---D-IASDLQN-VID--ADD-------LSLILESK-**

**Camelina_XP_010518230**  **------LIIKDSLLRYIAAFPVALKCHV-IYGS---D-IASDLQN-VID--EDD-------LSLILQSK-**

**Camelina_XP_010506566**  **------LLIKDSLLRYIAAFPVALKCHV-IYGS---D-IASDLQN-VID--ADD-------LSLILQSK-**

290 300 310 320 330 340 350

....|....|....|....|....|....|....|....|....|....|....|....|....|....|

**Galdieria_XP_005709103**  **--------------------------------------------NPPLFVLFQLSMQIKAAFRETTNVSD**

**Ceratitis_XP_004532120**  **--------------------------------------------TPCNALLQEIGLYLYHNRPDTRV---**

**Beauveria_KGQ13303**  **--------------------------------------------APCNRILMLMGSWLNQQREKGVISDV**

**Pluralibacter_AIR01448**  **--------------------------------------------SPANRILQLMAGWLAAHRRRGALSDI**

**Escherichia_UniProtP76146**  **--------------------------------------------SPANRILLIMGEWLAVQRRNGQLSDI**

**Citrobacter_KDF06924**  **--------------------------------------------SPANRILLIMGEWLATRRRDGQLSDI**

**Enterobacter_MGH16**  **--------------------------------------------SPANRILLIMGEWLAVRRRNGQLSDI**

**Exophiala_XP_009152408**  **---DQTPLKPKQYSRWKAVGEYLGISFAESNPRKLIKRSRDNLGNLPLEILTYLSAYMDEVMENGQLKMP**

**Rhizophagus_ESA24108**  **-EMDPSSE---------------------------------VNHNLPLEITLYLCSYIDNKRQNNKVDVP**

**Rhizophagus_EXX69703**  **-DQDLTENKIRENLKLGRQSSLKMFKPKVKPHQRRKGTPIPVSHNLPLEITLYLSSYIDTLAQKKKTDVP**

**Rhizophagus_EXX68848**  **YESDITHA-------QKSGWYTRLFRRKLKPHEREKEDHSLAEQNLPLDITLYLSSYIDTQVKNSSIDVP**

**Synechocystis_UniProtP72926_BEST** **--------------------------------------------NPPLEIAFWISNYLQREYDQNNINAY**

**Pseudanabaena_WP_009626831** **--------------------------------------------HPPLEIAFWLSSYLQEQTQQGKLDRY**

**Acaryochloris_WP_012165290** **--------------------------------------------HPPLQITFWIGMHLQQALQQGYIDSN**

**Synechococcus_WP_030007083**  **--------------------------------------------NRPLDIQFWLRTYLHQQLKLKNFGDA**

**Synechococcus_WP_038026347** **--------------------------------------------NRPFDIIFWIRCYLQKVLSKGEITES**

**Emiliania_XP_005770556**  **--------------------------------------------HKPMALLGLVSASLHD-SGRTGLDTV**

**Phaeodactylum_XP_002178861** **--------------------------------------------HRPNRALFDLSVAIEN-LPMHFMRKN**

**Phaeodactylum_XP_002180738** **--------------------------------------------HRPNRALFDLSVAIEN-LPMHFLRKN**

**Chlamydomonas_XP_001697952** **--------------------------------------------HRPVRAIHAISQIIQS-VPMSSIHQQ**

**Chlorella_XP_005843048**  **--------------------------------------------HRPNYTCQVLTAIIRA-AQ---LPGG**

**Chlorella_XP_005849094**  **--------------------------------------------HRPNYCCQVLTEVLRE-AQ---LPAA**

**Chlorella_XP_005845811**  **--------------------------------------------HRPNYCMQVLTACIKQ-AQ---LPAA**

**Chlorella_XP_005845925**  **--------------------------------------------HRPNYCMQVLTACLKQ-AQ---LPAA**

**Chlorella_XP_005845524**  **--------------------------------------------HRPNYCLQVMSEIVHS-AHLSALPLP**

**Chlorella_XP_005848889**  **--------------------------------------------HRPNFVLQVMAETVRA-ARPNELCRM**

**Physcomitrella_XP_001754623** **--------------------------------------------NQPIYVLQVISEIISQ-CH---IPNW**

**Physcomitrella_XP_001762184** **--------------------------------------------NRPIWVLQVISDIINE-CQ---ITPW**

**Physcomitrella_XP_001757529** **--------------------------------------------HRPNYILQVMSELISQ-CQ---ISQW**

**Physcomitrella_XP_001757527** **--------------------------------------------HRPNYVMQVMSELIKQ-CK---VSEW**

**Physcomitrella_XP_001760828** **--------------------------------------------HRPNYILQVLSELVDK-CN---LSEW**

**Physcomitrella_XP_001763440** **--------------------------------------------HRPNYILQVLSELINQ-CH---ISQW**

**Physcomitrella_XP_001785122** **--------------------------------------------HRPNYVLHVMSDTIKH-CR---ISKW**

**Selaginella_XP_002962243**  **--------------------------------------------HRPNCLLQMIFQIIDG-LS---LGET**

**Selaginella_XP_002965156**  **--------------------------------------------HRPNCLLQMIFQIIDG-LS---LGET**

**Physcomitrella_XP_001761167** **--------------------------------------------HRPNCLIQLITHSLKC-IQ---LEDG**

**Physcomitrella_XP_001754349** **--------------------------------------------HRPNCLIQLMTLSLKS-IK---FEDG**

**Picea_ABR16579**  **--------------------------------------------HPPFCIIQFMSECLER-VH---LADS**

**Oryza_NP_001048682**  **--------------------------------------------HRPRCIIEFISQSLYM-LD---FDEN**

**Zea_ACL54730**  **--------------------------------------------HRPRCIIEFIAQSLQM-LD---LDEQ**

**Zea_NP_001143185**  **--------------------------------------------HRPRCIIEFIAQSLQM-LD---LDEQ**

**Nicotiana_XP_009779200**  **--------------------------------------------HRPRCVIGFISQCLQS-LN---LEGT**

**Solanum_XP_004240104**  **--------------------------------------------HRPRCIIGFIAQSLQS-LN---LEGT**

**Glycine_XP_003521899**  **--------------------------------------------HRPRCIIEFISQSIRL-LK---LEES**

**Glycine_XP_003529947**  **--------------------------------------------HRPRCIIEFISQSIRL-LK---LEES**

**Medicago_KEH18868**  **--------------------------------------------HRPRCTIEFISQSIRL-LK---LEDS**

**Vitis_XP_002273844**  **--------------------------------------------HRPRCIIEFISQSLQL-LN---LDDA**

**AtVCCN1_At3g61320**  **--------------------------------------------HRPRCVIEFISQSIQL-LK---LDDA**

**Brassica_Bra007596**  **--------------------------------------------HRPRCVIEFISQSLQL-LK---LDDT**

**Brassica_Bra014443**  **--------------------------------------------HRPRCVIEFISQSLQL-LK---LDDT**

**Camelina_XP_010512468**  **--------------------------------------------HRPRCVIEFISQSIQL-LK---LDDS**

**Camelina_XP_010413427**  **--------------------------------------------HRPRCVIEFISQSIQL-LE---LDDS**

**AtVCCN2_At2g45870**  **--------------------------------------------HRPRCVIQFISQSLQL-LN---LDST**

**Brassica_Bra039307**  **--------------------------------------------HRPRCVIQFISQSLQL-LN---LDST**

**Camelina_XP_010508100**  **--------------------------------------------HRPRCVIQFISQSLQL-LN---LDST**

**Camelina_XP_010518230**  **--------------------------------------------HRPRCVIQFISQSLQL-LN---LDST**

**Camelina_XP_010506566**  **--------------------------------------------HRPRCIIQFISQSLQL-LN---LDSS**

360 370 380 390 400 410 420

....|....|....|....|....|....|....|....|....|....|....|....|....|....|

**Galdieria_XP_005709103**  **WVVQRA-------------------KMEWQLSSLMEVLSGCERIITTPVPLGYSRHTSRFLSLWCFTFPL**

**Ceratitis_XP_004532120**  **--YQ---------------------NYNHYLNQIGMIQGGCERISNTPIPFAYSLLLHRTVAIFCAIYPL**

**Beauveria_KGQ13303**  **-LWR---------------------SLDNHLNELSAVSGGCERIISTPMPFAYSLIQHRTVYIFCIMLPF**

**Pluralibacter_AIR01448**  **-LFS---------------------SLSNRLAEMSAVQAGCERIANTPLPFAYSLILHRTVYLFCIMLPF**

**Escherichia_UniProtP76146**  **-LFI---------------------SLNDRLNDISAVLAGCERIAYTPIPFAYTLILHRTVYLFCIMLPF**

**Citrobacter_KDF06924**  **-LFH---------------------SLNNRLNDMSAVLAGCERIANTPVPFAYTLILHRTVYLFCIMLPF**

**Enterobacter_MGH16**  **-LFH---------------------SLNNRLNDMSIVLSGCERIATTPVPFAYTLILHRTVYLFCIMLPF**

**Exophiala_XP_009152408**  **VHQT---------------------HAMNNITALADVLTGTERILTTPVPIAYSISISQITWVYILVLPF**

**Rhizophagus_ESA24108**  **-TTN---------------------SMYGALNTLIDCLTQFERILRSPIPLAYSIHLTQTVWIYCLSLPF**

**Rhizophagus_EXX69703**  **-TTN---------------------SMYAALNTMVDCLTQFERILRSPIPLAYSIHLKQTVWIYCLSLPF**

**Rhizophagus_EXX68848**  **-ITN---------------------QLLASLNGLCDCLSQFERILRSPIPLAYSIHLSQTVWIYCLSLPF**

**Synechocystis_UniProtP72926_BEST** **-QLT---------------------AMLRLVDTMVDVLGSCERILKTPIPLAYAIHLRQLIFLYCFITPF**

**Pseudanabaena_WP_009626831** **-QLN---------------------DMIQLLHQMVDVVGICERILRTPIPLAYAIHLKQLLMIYCLSLPF**

**Acaryochloris_WP_012165290** **-QAS---------------------NLDQALGKMIEGISSCERIRSTPLPIAYRIYLKRLILIYCVGLPF**

**Synechococcus_WP_030007083**  **-QLN---------------------MTTGMLNNLTDSVSGCERILTTPIPITYRVYLKRLILIYCFGLPF**

**Synechococcus_WP_038026347** **-KVG---------------------AIDNMLNQLTGGVSGCERIITTPIPITYRVYLKRLILIYCIGLPF**

**Emiliania_XP_005770556**  **-QAS---------------------KLDQTLSLLTDYLGKCERIVKTPLPLVYTRHTARFLSWWLLFLPV**

**Phaeodactylum_XP_002178861** **-------------------------EIHNAATIFEDNLGSSERLLTSPIPLFYARHTARFLGVWLLLMPF**

**Phaeodactylum_XP_002180738** **-------------------------QVHQAVTIFEDNLGSSERLLTSPVPLFYSRHTARFLSFWLLLLPF**

**Chlamydomonas_XP_001697952** **-------------------------QMSNNLTFFHDVLGGCERLLRAPIPVSYTRHTARFLFAWLTLLPF**

**Chlorella_XP_005843048**  **-KVDMNDSFANVKASAA-------FRMDENLTQYADVTGGCERILRTPVPLSYSRHNSRFLIIWLTLLPF**

**Chlorella_XP_005849094**  **-VTSPTDSTGCVPAGAA-------YRMDENLTVFEDVTGGCERLLRTPIPLAYTRHTSRFMMAWLTILPF**

**Chlorella_XP_005845811**  **-VTSNRDSYGAVPAGAA-------YRMDENLTVYSDVTGGCERILRTPVPLSYTRHTSRFMMIWLTLLPF**

**Chlorella_XP_005845925**  **-VTSNRDSYGAVPAGAA-------YRMDENLTVYSDVTGGCERILRTPVPLSYTRHTSRFMMIWLTLLPF**

**Chlorella_XP_005845524**  **-VARGAVVASGAPALAAAGWEGTRYRMDENLTAMEDILGACERILRAPIPLSYTRHTSRFMMIWLTLLPF**

**Chlorella_XP_005848889**  **-------------------------RMDDNLTFFEDAMGSCERILRTPIPLSYTRHTSRFLLVWLILLPF**

**Physcomitrella_XP_001754623** **-EKI---------------------CMDANLTQFHDNVGACERIFKTPIPIAYTRMTSRMLIMWHLALPY**

**Physcomitrella_XP_001762184** **-ERI---------------------AMDKNITQFHDNVGACERIFKTPIPVAYTRLTSRVLTLWHLVLPF**

**Physcomitrella_XP_001757529** **-EKI---------------------TMDKNITAFHDNVGACERIFKTPIPLAYTRLTSRMLMFWHLALPV**

**Physcomitrella_XP_001757527** **-ESM---------------------SMDRNLTQFHDNVGACERLFKTPIPVAYTRLTSRVLSLWHISLPF**

**Physcomitrella_XP_001760828** **-EKM---------------------AMDENITTFHDNVGACERILKTPIPLAYTLVTSRFLILWHLVLPF**

**Physcomitrella_XP_001763440** **-EKM---------------------SMDENITTFHDNVGACERILKTPIPIAYTLVTSRFLILWHSALPL**

**Physcomitrella_XP_001785122** **-ESK---------------------SMDRNITQFHDNVGACERLFKTPIPVAYTRMISRFLSIWHFLLPL**

**Selaginella_XP_002962243**  **-QQV---------------------LLHENISTYNRSVSVCERLIRTPIPLSYTRLTSRFLILWHLGLPI**

**Selaginella_XP_002965156**  **-QQV---------------------LLHENISTYNRSVSVCERLIRTPIPLSYTRLTSRFLILWHLGLPI**

**Physcomitrella_XP_001761167** **-ERS---------------------LLDANISQFNESISICERIIRTPIPLAYTRLTSRILVLWHLALPI**

**Physcomitrella_XP_001754349** **-EGM---------------------QLDANISQFNESISVCERLIRTPIPLAYTRLTSRILVLWHLSLPV**

**Picea_ABR16579**  **-KQN---------------------ILDSHISQFNDSISVCERLIGIPIPLSYTRLTSRFLVLWHLTLPI**

**Oryza_NP_001048682**  **-KRN---------------------IMESKLSCFLEGISVCEQLIGIPIPLSYTRLTSRFLVLWHLTLPV**

**Zea_ACL54730**  **-KRS---------------------IMVSKLSCFLEGIGVCEQLMGIPIPLAYTRLTSRFLVLWHLTLPI**

**Zea_NP_001143185**  **-KRS---------------------IMVSKLSCFLEGIGVCEQLMGIPIPLAYTRLTSRFLVLWHLTLPI**

**Nicotiana_XP_009779200**  **-KLT---------------------QLESKISCFHEGIGVCEQLAGIPIPLSYTRLTSRFLVLWHLTLPI**

**Solanum_XP_004240104**  **-ILS---------------------QLESKISCFHEGIGVCEQLLGIPIPLSYTRLTSRFLVLWHLTLPI**

**Glycine_XP_003521899**  **-RRN---------------------VLESKITCFHEGIGICDQLMGIPIPLAYTRLTSRFLVLWHLTLPI**

**Glycine_XP_003529947**  **-RRN---------------------ALESKMTCFHEGIGICDQLMGIPIPLAYTRLTSRFLVLWHLTLPI**

**Medicago_KEH18868**  **-RRS---------------------ILESKITCFHEGIGLCEQLLGIPIPLSYTRLTSRFLVLWHLTLPI**

**Vitis_XP_002273844**  **-KRH---------------------VLESKLSCFHEGIGVCEQLMGIPIPLSYTRLTSRFLVLWHLTLPI**

**AtVCCN1_At3g61320**  **-KRD---------------------LLESKMLHLHEGIGVCEQLMGIPIPLSYTRLTSRFLVFWHLTLPI**

**Brassica_Bra007596**  **-KRD---------------------LLESKMLHLHEGIGVCEQLMGIPIPLAYTRLTSRFLVFWHLTLPI**

**Brassica_Bra014443**  **-KRD---------------------LLESKMLHLHEGIGVCEQLMGIPIPLSYTRLTSRFLVFWHLTLPI**

**Camelina_XP_010512468**  **-KRD---------------------LLESKMLHLHEGIGVCEQLMGIPIPLSYTRLTSRFLVFWHLTLPI**

**Camelina_XP_010413427**  **-KRD---------------------LLESKMLHLHEGIGVCEQLMGIPIPLSYTRLTSRFLVFWHLTLPI**

**AtVCCN2_At2g45870**  **-KID---------------------MLETKMMQLQEGIGVCEQLMGIPIPLSYTRLTSRFLVLWHLTLPV**

**Brassica_Bra039307**  **-KID---------------------TL-------------------------------------------**

**Camelina_XP_010508100**  **-KID---------------------MLESKMMQLQEGIGVCEQLMGIPIPLSYTRLTSRFLVLWHLTLPV**

**Camelina_XP_010518230**  **-KID---------------------MLESKMMQLQEGIGVCEQLMGIPIPLSYTRLTSRFLVLWHLTLPV**

**Camelina_XP_010506566**  **-KID---------------------MLESKMMQLQEGIGVCEQLMGIPIPLSYTRLTSRFLVLWHLTLPV**

430 440 450 460 470

....|....|....|....|....|....|....|....|....|....|

**Galdieria_XP_005709103**  **LVVSHF--KF---LTVPVTAFVCWSLFAIEEIGHVIEDPFLEGTQKLPIK**

**Ceratitis_XP_004532120**  **LLISNF--GV---LTLLFSLFTTYALLALDAIATELEDPFGHEDNDLPLG**

**Beauveria_KGQ13303**  **ALVTDL--HY---MTPFVSVFVSYTFISLDSLAEELEDPFGTEDNDLPLD**

**Pluralibacter_AIR01448**  **ALVSDL--HY---MTPFVSVLISYTFISLDALAEELEEPFGFENNDLPLD**

**Escherichia_UniProtP76146**  **ALVVDL--HY---MTPFISVLISYTFISLDCLAEELEDPFGTENNDLPLD**

**Citrobacter_KDF06924**  **ALVVDL--HY---MTPFISVLISYTFISLDALAEELEDPFGTENNDLPLD**

**Enterobacter_MGH16**  **ALVVDL--HY---MTPFVSALISYTFISLDTLAEELEDPFGTEDNDLPLD**

**Exophiala_XP_009152408**  **QLYNSL--GW---VSIFGTVLAAYIILGLAAIGYEIENPFGNDVNDLPLD**

**Rhizophagus_ESA24108**  **QLVDSL--KY---ITIPIVFLASFILIGILHIGGEIENPFGYDENDLDLD**

**Rhizophagus_EXX69703**  **QLIKNL--HY---ITIPVVFLASMILMGIELIGGEIENPFGYDENDLELD**

**Rhizophagus_EXX68848**  **FLVSGT--RW---ATIIVVFFVALILFGLERIGAEIENPFGYDANDLDLD**

**Synechocystis_UniProtP72926_BEST** **QIVNTL--HW---ATAFVVGIIAFTVFGIEEIGVEIENPFGHDANDLPLD**

**Pseudanabaena_WP_009626831** **QMVDQL--EW---MTAPIVALISFTLLGIEEIGIQIEDPFGHDTNDLPLD**

**Acaryochloris_WP_012165290** **RWVPEI--HG---WAVPMVAVVSFILLGLEEVGRELDNPFGQDANDLPID**

**Synechococcus_WP_030007083**  **RLVPEM--TW---WAIPIVAVVSFLLLGVEEVARELENPFGFDVNDLPLD**

**Synechococcus_WP_038026347** **KTIPEL--TW---WSLPIVAVVSFLLLGVEEVARELENPFGYNVNDLPLD**

**Emiliania_XP_005770556**  **CLYNQLRANW---MIVPVSGLIGFFLVGIEDLGNQIEEPFSI----LPLT**

**Phaeodactylum_XP_002178861** **CLYDPFAGSWNHVGMIPATALISIFLFGIEELATSMEEPFTI----LPMQ**

**Phaeodactylum_XP_002180738** **ALWDPFAGTWNHVGMIPATAVISIFLFGIEELATQMEEPFTI----LPMQ**

**Chlamydomonas_XP_001697952** **ALYPTT--GW---GVVPVCTGIAAVLCGIEEIGVQCEEPFGI----LPLD**

**Chlorella_XP_005843048**  **TLWDQC--HW---FTLPVTGLVAFLLLGIKEIGVVVEEPFSI----LPLE**

**Chlorella_XP_005849094**  **ALWDTC--GW---AMLPVMAIVAFVLLGIEEIGVSIEEPFSI----LPLE**

**Chlorella_XP_005845811**  **TLWDNC--GW---AMLPITFIVSFLLLGIEEIGVSIEEPFTI----LPLE**

**Chlorella_XP_005845925**  **TLWDNC--GW---AMLPITFIVSFLLLGIEEIGVSIEEPFTI----LPLE**

**Chlorella_XP_005845524**  **SLWDNC--GW---ASVPLCGIIAFLLLGIEEIGVSIEEPFSI----LPLE**

**Chlorella_XP_005848889**  **TLWAAY--SW---FSILLSGIFAFLMFGIDEIGVQIEEPFGW----VAAT**

**Physcomitrella_XP_001754623** **GLWNDC--RW---LTIPATFMSSAALFYIEQVGVVIEEPFCI----LALD**

**Physcomitrella_XP_001762184** **ALWETC--GW---HTITVSFVSSAALFYIEEVGVMIEEPFSI----LALS**

**Physcomitrella_XP_001757529** **GLWNTC--GW---LTIPVSFMSAAALFYIEEVGVLIEEPFWI----LPLL**

**Physcomitrella_XP_001757527** **ALWNSC--HW---LTIPATFFSSAALFYIEEVGVLIEEPFWI----LALM**

**Physcomitrella_XP_001760828** **ALWATC--HW---LTIPVTFLTATALFYIEEVGVLIEEPFWI----LPLL**

**Physcomitrella_XP_001763440** **ALWNDC--GW---LTIPATFLTGMALFYIEEVGVVIEEPFWI----LPLG**

**Physcomitrella_XP_001785122** **ALWNSC--RW---LTIPVTFVSGVGLFCIEEVGVLIEDPSTS----L---**

**Selaginella_XP_002962243**  **ALWDTC--NW---LVIPSTFFSSAALFCIEEVGVLIEEPFPM----LALD**

**Selaginella_XP_002965156**  **ALWDTC--NW---LVIPSTFFSSAALFCIEEVGVLIEEPFPM----LALD**

**Physcomitrella_XP_001761167** **VLWDDC--QW---VVVPATFISAASLFCIEEVGVLIEEPFPI----LALD**

**Physcomitrella_XP_001754349** **VLWDDC--HW---VVVPATFISAASLFCIEEVGVLIEEPFPI----LALD**

**Picea_ABR16579**  **ILWDEC--EW---IVVPATFVSAASLFCIEEVGVLIEEPFPM----LALE**

**Oryza_NP_001048682**  **ILWDEC--KW---IVVPATFISAASLFCIEEVGVLIEEPFPM----LALD**

**Zea_ACL54730**  **ILWEEC--KW---IVVPATFISAASLFCIEEVGVLIEEPFPM----LALD**

**Zea_NP_001143185**  **ILWEEC--KW---IVVPATFISAASLFCIEEVGVLIEEPFPM----LALD**

**Nicotiana_XP_009779200**  **ILWDDC--HW---IVVPATFISAASLFCIEEVGVLIEEPFPM----LALD**

**Solanum_XP_004240104**  **ILWDDC--HW---IVVPATFISAASLFCIEEVGVLIEEPFPM----LALD**

**Glycine_XP_003521899**  **ILWDDC--HW---IVVPATFISAASLFCIEEVGVLIEEPFAT----LALD**

**Glycine_XP_003529947**  **ILWDDC--HW---IVVPATFISAASLFCIEEVGVLIEEPFAT----LALD**

**Medicago_KEH18868**  **ILWDDC--HW---IVVPATFISAASLFCIEEVGVLIEDPFPM----LALN**

**Vitis_XP_002273844**  **ILWEDC--NW---IVVPATFISAASLFCIEEVGVLIEEPFPM----LALD**

**AtVCCN1_At3g61320**  **ILWDEC--HW---IVVPATFISAASLFCIEEVGVLIEEPFPM----LALD**

**Brassica_Bra007596**  **ILWDEC--HW---IVVPATFISAASLFCIEEVGVLIEEPFPM----LALD**

**Brassica_Bra014443**  **ILWDEC--HW---IVVPATFISAASLFCIEEVGVLIEEPFPM----LALD**

**Camelina_XP_010512468**  **ILWDEC--HW---IVVPATFISAASLFCIEEVGVLIEEPFPM----LALD**

**Camelina_XP_010413427**  **ILWDEC--HW---IVVPATFISAASLFCIEEVGVLIEEPFPM----LALD**

**AtVCCN2_At2g45870**  **ILWDDC--HW---NVVPATFISAASLFCIEEVGVLIEEPFSM----LALD**

**Brassica_Bra039307**  **-------------------------------VGVLIEEPFSM----LALD**

**Camelina_XP_010508100**  **ILWDDC--HW---NVVPATFISAASLFCIEEVGVLIEEPFSM----LALD**

**Camelina_XP_010518230**  **ILWDDC--RW---NVVPATFISAASLFCIEEVGVLIEEPFSM----LALD**

**Camelina_XP_010506566**  **ILWDDC--HW---NVVPATFISAASLFCIEEVGVLIEEPFSM----LALD**
